# Computational Pipeline Reveals Nature’s Untapped Reservoir of Halogenating Enzymes

**DOI:** 10.1021/acsomega.6c02136

**Published:** 2026-08-11

**Authors:** Judit Szenei, Ashleigh Burke, Anne Liong, Aleksandra Korenskaia, April L. Lukowski, Nadine Ziemert, Pablo I. Nikel, Pedro N. Leão, Bradley S. Moore, Tilmann Weber, Kai Blin

**Affiliations:** † The Novo Nordisk Foundation Centre for Biosustainability, 5205 Technical University of Denmark, Kgs. Lyngby 2800, Denmark; ‡ Department of Bioengineering and Biomedicine, Technical University of Denmark, Kgs. Lyngby 2800, Denmark; § Center for Marine Biotechnology and Biomedicine, Scripps Institution of Oceanography, 8784 University of California San Diego, La Jolla, California 92093, United States; ∥ Interdisciplinary Centre of Marine and Environmental Research (CIIMAR), 26706 University of Porto, Terminal de Cruzeiros do Porto de Leixões, Avenida General Norton de Matos, S/N, Matosinhos 4450-208, Portugal; ⊥ ICBAS − School of Medicine and Biomedical Sciences, University of Porto, Porto 4050-313, Portugal; # Translational Genome Mining for Natural Products, Interfaculty Institute of Microbiology and Infection Medicine Tübingen (IMIT), Interfaculty Institute for Biomedical Informatics (IBMI), 9188 University of Tübingen, Tübingen 72074, Germany; ∇ Department of Pharmaceutical Sciences, Skaggs School of Pharmacy and Pharmaceutical Sciences, University of California San Diego, La Jolla, California 92093, United States; ○ German Center for Infection Research (DZIF), Partner Site Tübingen, Tübingen 72076, Germany; ◆ The Novo Nordisk Foundation Biotechnology Research Institute for the Green Transition (BRIGHT), Technical University of Denmark, Kgs. Lyngby 2800, Denmark

## Abstract

Microbial halogenated natural products (hNPs) hold ecological, agricultural, and biomedical relevance. The hNP-producing potential of an organism can be assessed by the precise prediction of halogenating enzymes, yet detailed annotations of halogenases are often missing from genomic and metagenomic data. We created a manually curated database (https://halogenases.secondarymetabolites.org/) containing information on the halide specificity, role, and position of verified catalytic residues and the results of mutagenesis studies of more than 120 experimentally validated or *in silico* inferred halogenases. The collection of experimental data supports a computational pipeline that allows family-, substrate-, and halide-scope-level annotation of halogenating enzymes by relying on functionally important residues, conserved motifs, and profile hidden Markov models (pHMMs). Our analysis with sequence similarity networks (SSNs) highlighted several underexplored clusters in the UniRef50 database. We further investigated a cluster of vanadium-dependent haloperoxidases because a halogenase from *Rhodopirellula baltica* (*Rhoba*VHPO), previously labeled as a hypothetical chloroperoxidase, clustered apart from the known chloroperoxidases and bromoperoxidases. The monochlorodimedone assay confirmed the chlorination activity of *Rhoba*VHPO and showed its preference for bromide. Our database and workflow provide extensive and scalable solutions for the systematic and precise annotation of halogenating enzymes in genomic and metagenomic data sets. The in-depth categorization of halogenases will improve the chemical structure prediction of microbial hNPs, supporting ecological assessments and natural product discovery.

## Graphical abstract

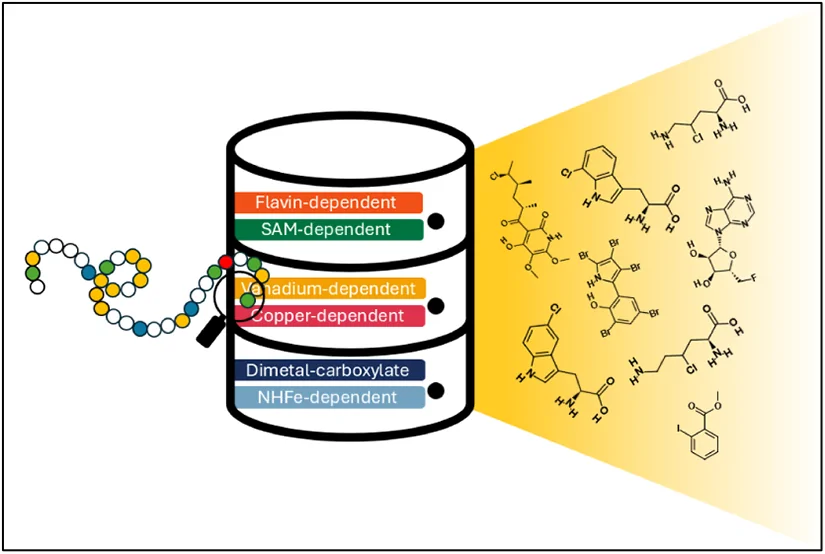

## Introduction

Microbes possess a large array of halogenating enzymes, making them important contributors to halogen cycling in marine and terrestrial environments.^1,2^ The variety of halogenated natural products (hNPs) that enter the biogeochemical cycles can be predicted by the precise annotation of halogenating enzymes. Identifying functionally important amino acids within the enzyme sequence can aid in the in-depth classification of halogenases. However, the diversity of catalytic residues, halide scope, substrate scope, and cofactor requirements makes it difficult to annotate halogenases with a systematic approach.

The reaction mechanisms of halogenases span across electrophilic, nucleophilic, and radical substitution^3,4^ with the utilization of organic and metal cofactors (Figure 1) to yield a chlorinated, brominated, iodinated, or fluorinated product.^3^ The wide variety of halide scope, substrate scope, and cofactors that halogenases showcase leads to distinct catalytic cycles, which are recognizable based on the functionally important amino acids. Relying on catalytic residues provides support for detailed predictions, such as inferring the substrate scope, halide scope, and selectivity.

**Figure 1:**
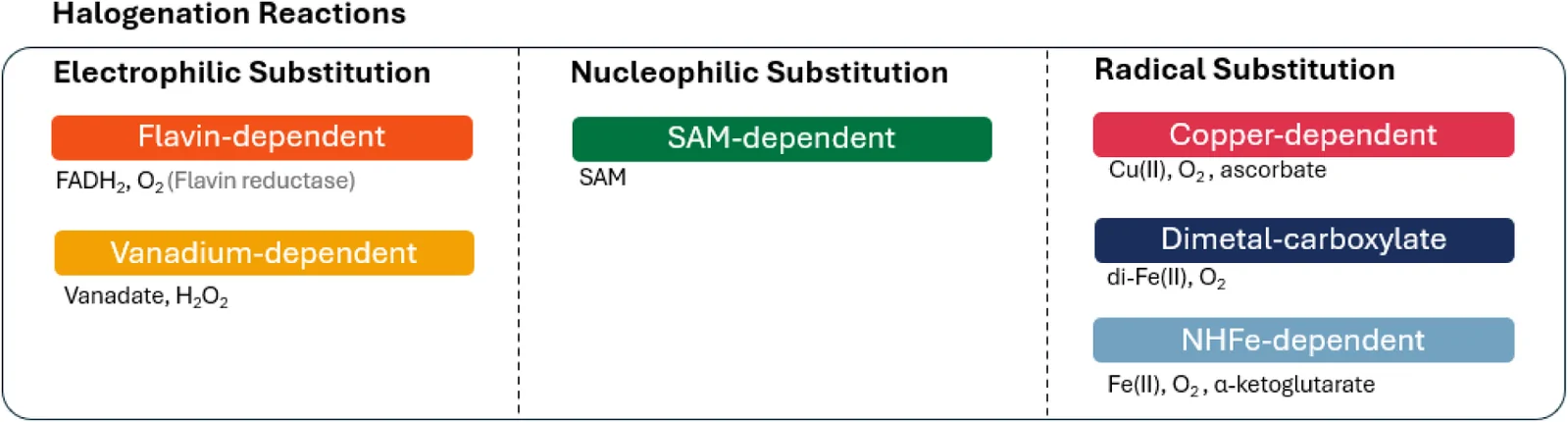
Cofactor requirements of halogenases. Halogenation occurs through electrophilic substitution, catalyzed by flavin- and vanadium-dependent enzymes^3^; nucleophilic substitution, catalyzed by SAM-dependent halogenases^3^; or radical substitution, catalyzed by copper,^19^ diiron,^23^ or monoiron halogenases^30^ ^,^.^43^ Halogenases comprise a diverse set of enzymes with inorganic and organic cofactor requirements (Flavin reductase coenzyme is written in gray, as there are FDHs active without the reductase partner^8^).

Halogenation through electrophilic substitution occurs with the help of a heme-oxo-Fe­(IV) species, reduced flavin, or a vanadate center by heme-dependent, flavin-dependent, or vanadium-dependent enzymes^3^ (Figure 1), respectively. The heme-dependent family provided the first described halogenase, produced by *Caldariomyces fumago*.^4^ The repertoire of halogenating enzymes was later expanded with flavin-dependent halogenases (FDHs), which are widespread across Fungi, Bacteria, and Archaea, with crystal structures available for more than 20 enzymes in the Protein Data Bank (RCSB.org).^5^ Two-component FDHs are the most common in sequence and structure databases,^6^ requiring a flavin-reductase partner. However, recent studies have revealed single-component members of the family that function without a reductase coenzyme^7^ ^,^.^8^ The substrate scope of FDHs extends to tryptophan, phenol, pyrrole, and terminal alkynes^7,9,10^ which can be free-standing (variant A enzymes) or tethered to an acyl carrier protein (ACP) (variant B enzymes).^3,11^ Only a few FDHs accept aliphatic substrates,^12,13^ but whether this is enabled through electrophilic substitution or a radical mechanism^14^ is debated. In contrast to flavin-dependent halogenases that are known for their substrate and regioselectivity,^10,14,15,16^ vanadium-dependent haloperoxidases (VHPOs) were often labeled as promiscuous. However, examples of VHPOs with both enantio- and regioselectivity^17,18^ have been reported. In summary, the substrate scope of FDHs and VHPOs mostly covers aromatic structures.^3^

In contrast to enzymes showcasing electrophilic halogenation, the group of radical halogenases contains several families that act on unactivated C atoms. Within this group, we find multiple iron-dependent and a recently discovered copper-dependent enzyme^19^ (Figure 1). Nonheme iron alpha-ketoglutarate (NHFe)-dependent members are the most studied, including both variant A (with free-standing substrates)^20,21^ and variant B (with substrates tethered to an ACP) biocatalysts.^22^ By contrast, the group of dimetal-carboxylate halogenases^23^ has gained attention only in recent years, primarily from cyanotoxin biosynthetic pathways.^24^ It is also the group most abundant in fatty acid-halogenating enzymes.^25^ Within the NHFe-dependent family, only HctB from hectachlorin biosynthesis is known for such substrate scope,^26,27^ joined recently by a copper-dependent halogenase discovered in atpenin biosynthesis.^28^

Enzymes catalyzing electrophilic, radical, and nucleophilic halogenation include chloride^3^-, bromide-,^29,30,31^ and iodide^19,32^ -utilizing proteins. However, S-Adenosyl-l-methionine (SAM)-dependent enzymes, the sole representatives of nucleophilic halogenases^3^, stand out for their fluorinating members.^33^ The adenine-binding region at the C-terminal with the RNAA motif sets the fluorinases apart from their homologues, which do not accept fluoride.^34^ It is a halide scope mostly seen in Actinomycetes,^28,33^ but *in silico* mining yielded an additional fluorinase from Archaea with a YYGG motif at the C-terminal.^35^ We can recognize nonfluorinating halogenases by an LNQG pattern in the same positions as the RNAA/YYGG motif in the alignment, where Lys could be replaced by Arg, or Val; Gln could be substituted by Arg; and Gly could be replaced by Ser or Asp residues.^34^

Several computational tools for identifying halogenase sequences exist, including the biocatalyst mining tool EnzymeMiner,^36^ which allows the specification of functionally important residues; annotation tools like BLAST^37^ and profile Hidden Markov Model (pHMM)-based^38^ solutions, such as HHPred^39^ and InterPro profiles,^40^ that rely only on homology. Although homology-guided approaches can indicate substrate scope, e.g., for flavin-dependent tryptophan halogenases,^41^ pHMMs integrated into InterPro are often too broad for inferring substrate scope or selectivity. Despite the abundant information on catalytic residues and conserved motifs from experimental studies, there was no tool that integrated this knowledge across several halogenase families, and neglecting catalytic information can lead to ambiguous or incorrect annotation.

We aimed to close the gap in the differing annotation tool availability for underexplored halogenase families and improve the annotation by collecting the functionally important residues and motifs and constructing activity-specific pHMMs. We demonstrated the potential of the computational pipeline by applying it to UniRef50.^42^ Furthermore, a haloperoxidase from *Rhodopirellula baltica* (*Rhoba*VHPO) was chosen for experimental study due to its clustering with VHPOs that possess characteristic iodoperoxidase catalytic residues. Monochlorodimedone (MCD) assay confirmed its annotation as a chloroperoxidase and uncovered its bromide preference, which was 5-fold greater than that of chloride. The developed computational pipeline and database support the in-depth annotation of halogenating enzymes across six enzyme families, enabling a more accurate prediction of the chemical structure of hNPs and the mining of novel biocatalysts.

## Results and Discussion

### Halogenase DB

To address the annotation gap among halogenating enzymes, we manually curated the Halogenase DB (Figure 2), which contains information about experimentally validated or *in silico*-inferred halogenases. The first layer of the DB contains the accession, producing organism, protein name, and halide scope. The second layer of the database includes the residues relevant to the structure or catalytic cycle of the enzyme. Furthermore, the second layer also reports the positions of these residues in the sequence as well as in the corresponding pHMM.

**Figure 2:**
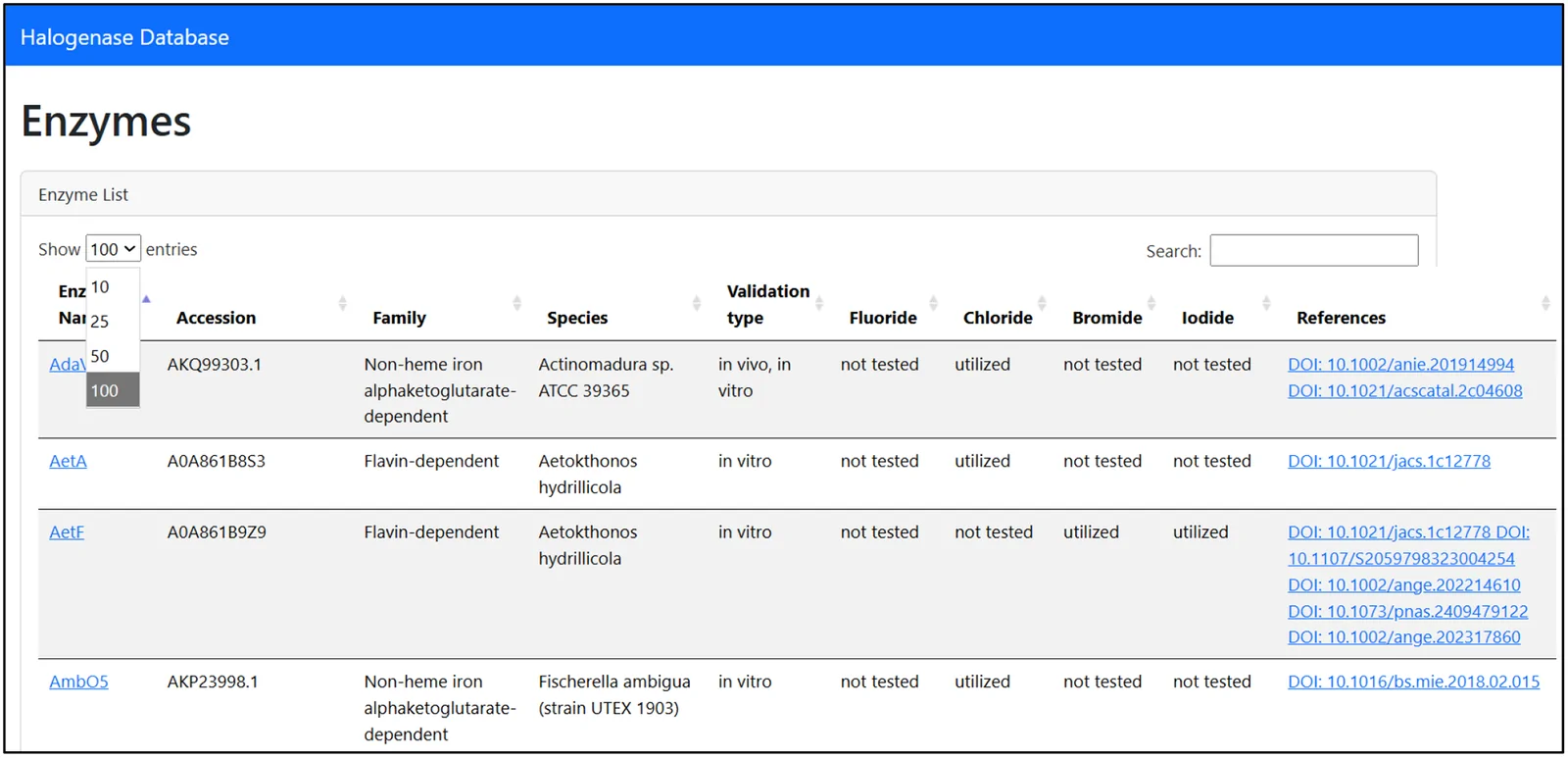
Halogenase DB. The first page of the database contains information on the name, accession (which can be RefSeq ID, UniProt ID, or PDB ID), the family the enzyme belongs to, the producing organism, its halide acceptance and references to studies of experimental validation, structure analysis, and discovery.

The data is stored in JSON format, which allows easy access to the database and scalability for new entries, as novel halogenases are being discovered and studied.

The DB (https://halogenases.secondarymetabolites.org) currently consists of 124 entries, more than double the halogenase entries in SwissProt. Copper-dependent (1 sequence), dimetal-carboxylate (7 sequences), SAM-dependent (7 sequences), NHFe-dependent (22 sequences), FDHs (65 sequences), and VHPOs (22 sequences) are represented in different distributions depending on the number and extent of published studies. Validation details are available for 94 enzymes, and mutagenesis or structure analysis studies are available for 50 proteins.

The Halogenase DB serves as the primary source of information for protein expression or enzymatic assays and highlights the pitfalls that arose in previous experimental processes or could result from misleading labeling. Misleading annotation can be exemplified by halogenases from *Acaryochloris marina* (*Am*VBPO) and from *Ascophyllum nodosum* (*An*VBPO). While VHPOs follow the naming convention of indicating the most electronegative halide the enzyme accepts, the two VHPOs labeled as bromoperoxidase actually utilize chloride.^44,45^ The chlorination activity differs between the two species.^44,45^ *An*VBPO tetrachlorinates phenolsulfonephthalein,^44^ while *Am*VBPO required such high chloride concentrations in the MCD assay that the kinetic studies could not be completed.^45^

Therefore, if available, the database includes information about the tested and preferred halides. The structured data on halide utilization also highlights the bias in halide testing, as out of 124 entries, only 11 enzymes were tested for fluoride. Future studies should systematically test all four halides to reduce this bias.

### Collection of Catalytic Residues and Conserved Motifs for Annotation

The computational pipeline integrates experimental information into a homology-based framework to enable in-depth halogenase annotation. In the case of FDHs and dimetal-carboxylate enzymes, the conserved motifs and essential residues for activity, inferred through *in silico* analysis and experimental validation, support the family-level categorization (SI Guide for the Residue and Motif Collection Chapter). The annotation of flavin-dependent enzymes is based on WxWxI (Table 1),^46,47^ a motif that prevents monooxygenation, and Fx.PxSx:G (Table 1),^32^ a motif that marks the tunnel in the protein structure.^3^ For the dimetal-carboxylate family, the annotation is based on the ExxQExxH and HxxDExxH motifs (Table 1).^23^ For certain subgroups, a more detailed activity prediction is possible with a few catalytic residues. The prediction of the number of halogenation events for FDHs is supported for pyrrole halogenases (Supporting Information (SI) Guide for the Residue and Motif Collection Chapter) through a pHMM. For VHPOs, the prediction of potential iodination activity and the selectivity of the enzyme rely on the residue collection (SI Guide for the Residue and Motif Collection Chapter ).

**Table 1:** Conserved Motifs and Catalytic Residues for Annotation

Enzyme family	Motif/Catalytic residue	Role (SI Guide for the residue and motif collection chapter)
Flavin-dependent	GxGxxG	Flavin-binding site
Flavin-dependent	GxGxxA	Conserved motif in unconventional FDHs
Flavin-dependent	WxWxI	Prevents monooxygenation activity
Flavin-dependent	Fx.Px.Sx.G	Key residues in the tunnel between the flavin binding site and the site of halogenation both in two-and single-component FDHs
NHFe-dependent	HxG/A	Catalytic triad
Dimetal-carboxylate	ExxQExxH	Contains the catalytic Gln specific to halogenases
Dimetal-carboxylate	HxxDExxH	Contains the putative active site
SAM-dependent	RNAA	C-terminal motif in bacterial fluorinases
SAM-dependent	YYGG	C-terminal motif in archaeal fluorinase
Vanadium-dependent	K324; S427 (in NapH1)	Characteristic to regio/stereoselective chloroperoxidases
Copper-dependent	HxxHC; HxxHC	The two motifs indicate the metal-binding site

In contrast to flavin-dependent and dimetal-carboxylate proteins, the family-level categorization of NHFe-dependent, SAM-dependent, and vanadium-dependent halogenases is strongly homology-based, with an optional step for the user to search for catalytic residues in validated enzymes. While conserved motifs and functionally important amino acids do support further categorization among these groups, the level of annotation differs across families, reflecting the availability of experimental data. A C-terminal motif enables the identification of potential fluorinases both in bacteria and archaea in the SAM-dependent family (Table 1).^35^ There is no halide-scope prediction step in the workflow for NHFe-dependent enzymes, but a smaller motif marking the catalytic triad (Table 1) helps differentiate halogenases from dioxygenase/hydroxylase homologues (Table 1).^48^ For VHPOs, the presence of a Lys and a Ser residue indicates the stereo/regioselectivity of the enzyme of interest (Table 1).^17^

As the catalytic residues responsible for halogenation activity among the copper-dependent enzymes remain uncharacterized, we cannot distinguish them from the hydroxylase homologues; however, family-level annotation is possible by searching for two HxxHC motifs.^19^ The two Cys residues (Cys173 and Cys200) are responsible for creating one of the two intramolecular disulfide bridges in the structure.^19^

While for FDHs, VHPOs, SAM- and iron-dependent enzymes, the workflow detects halogenases among homologous sequences with nonhalogenation activity, for copper-dependent enzymes, we suggest using the categorization only for family-level annotation of halogenating enzymes with validated activity (SI Guide for Residue/Motif-based Categorization Chapter).

### Curated pHMMs

The collection of sequences for pHMM curation relied on an extensive review covering several halogenase families from Crowe and Molyneux et al.^3^, a family-specific review article for vanadium-dependent haloperoxidases (Winter and Moore et al.),^49^ and a research article discussing the diversity and distribution of dimetal-carboxylate enzymes (Eusebio, Regio, Glasser, Castelo–Branco, Balskus, and Leão).^50^ Starting from there, we used the names of the enzyme families and the keyword “halogenase” to focus primarily on research articles (Figure 3). Further querying was done in the local version of MIBiG 4.0 ^51^ for halogenated compounds, and on validated and putative halogenases in SwissProt and UniProt (Figure 3).^52^

**Figure 3:**
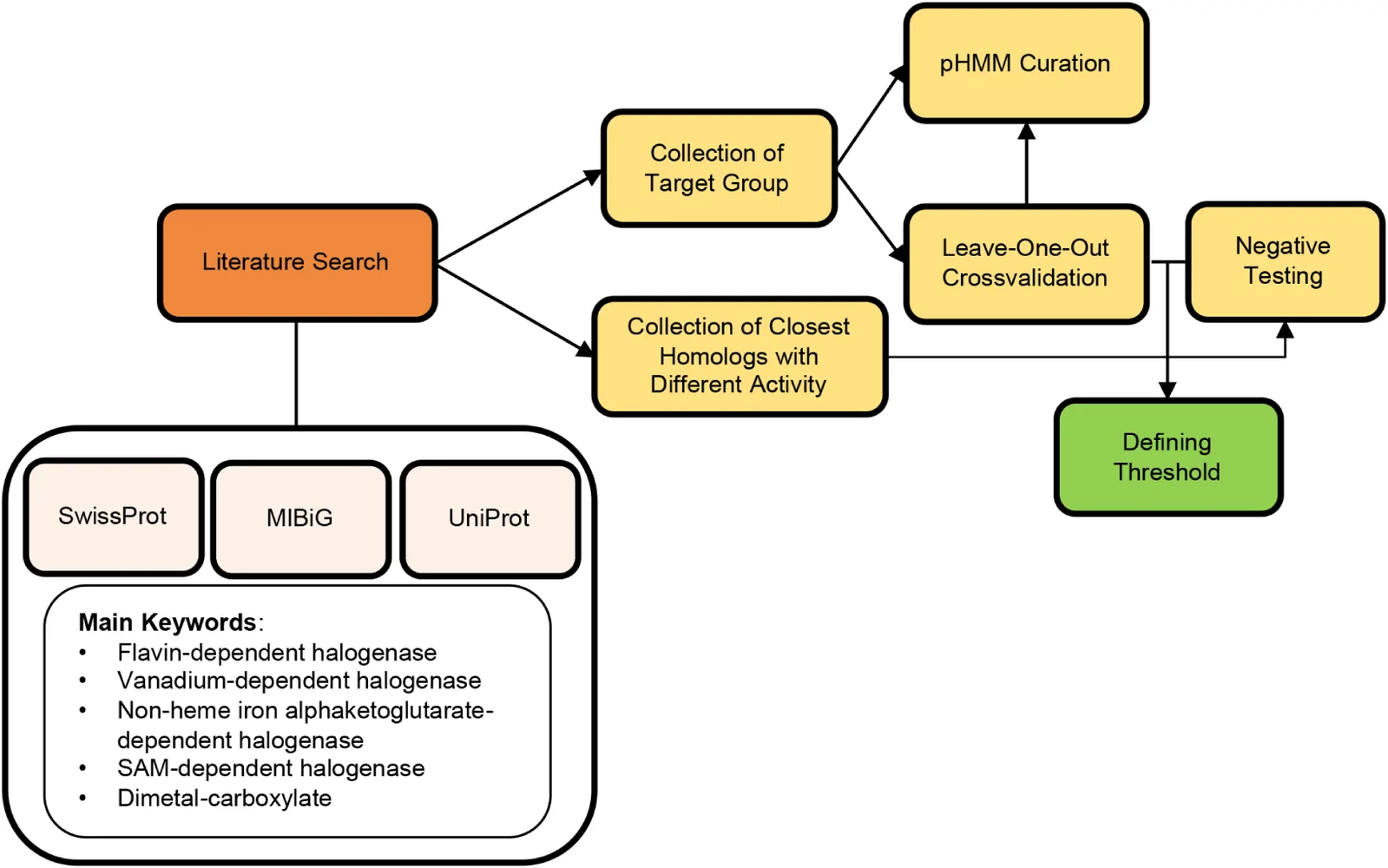
Process of sequence collection and pHMM curation. Literature search relied mainly on the name of the enzyme family and the word “halogenase” as keywords. The search was further aided by browsing both UniProt and the validated sequences in SwissProt^52^ as well as recovering halogenated hNPs from MIBiG.^51^ The sequences were organized into FASTA files according to their activity, and the closest homologues with different activity served as negative tests when testing the pHMMs to curate family-, substrate-, and halide-scope-specific models. Both negative testing and leave-one-out cross-validation helped us recommend thresholds for the bitscore to indicate confidence in the prediction.

After collecting the sequences and generating MSAs for each of the targeted halogenase families, we curated the pHMMs. The number of pHMMs differs for each group of halogenases, depending on the available sequence data and the similarity between enzymes with distinct substrate and halide scopes (Table 2). We performed leave-one-out cross-validation to define bitscore thresholds based on the left-out sequence, which had the lowest score to the pHMM, and based on the scores of the sequences from the negative group (Figure 3). These thresholds serve as guiding points to exclude enzymes with different activities compared to the target group of the pHMM (Table 2).

**Table 2:** Recommended pHMM Cutoffs

Enzyme Family	pHMM	Recommended bitscore cutoff	MSA (for building the pHMM)
**SAM-dependent**	Nonfluorinases	250	Whole sequence
	Fluorinases	195	Whole sequence
**NHFe-dependent**	Indole alkaloid (variant A)	600	Whole sequence
	Nucleotide (variant A)	200	Cut
	Small amino acids (variant A)	300	Whole sequence
	Variant B	200	Whole sequence
**Dimetal-carboxylate**	General	No cutoff	Whole sequence
**Flavin-dependent**	General	No cutoff	Cut
	Unconventional	No cutoff	Whole sequence
	Tryptophan-5	350 (for mibH-like sequences); 850	Whole sequence
	Tryptophan-6/7	750	Whole sequence
	Orsellinic acid-like	500	Whole sequence
	Tyrosine/Hpg-like	305	Cut
	Pyrrolic	400	Cut
**Vanadium-dependent**	General		Whole sequence
	Selective chloroperoxidases	600	Cut
	Nonselective chloroperoxidases	700	Cut
	Bromoperoxidases	500	Whole sequence
	Iodoperoxidases	700	Whole sequence
**Copper-dependent**	General	No cutoff	Whole sequence

The computational pipeline offers tailored ways for the analysis of the covered enzyme families. For the SAM-dependent family, the pHMMs predict fluorination and nonfluorination activity. Nonfluorinating enzymes are often labeled as chlorinases, stemming from the fact that they are not routinely tested for bromide or iodide. To our knowledge, the only known example of a SAM-dependent halogenase tested for all four halides is SaIL, a halogenase from *Salinispora tropica*, which accepts all halides, except fluoride.^20^ Using the labels “fluorinase” or “non-fluorinase” helps to describe halide utilization more clearly.

While the prediction of halide utilization is not included in the workflow for NHFe-dependent halogenases, the pipeline can indicate whether the substrate is free-standing or if an ACP is required for the halogenation to take place. For variant A enzymes, the workflow includes substrate scope predictions by relying on nucleotide-, indole alkaloid-, and small amino acid-specific pHMMs. The pHMM-based substrate prediction is feasible for variant A enzymes, as they cluster based on substrate scope on the phylogenetic tree (SI). The workflow does not consider catalytic residues or conserved motifs, and we recommend using the features of the pipeline where the user can compare the sequences to validated enzymes in which the functionally important amino acids were inferred. As the residues and structural motifs are unknown for deciding between variant A and variant B groups, the pHMMs provide a new layer of categorization.

Because of the limited number of experimental studies, the in-depth annotation of dimetal-carboxylate halogenases is not possible. Therefore, the workflow for diiron halogenases relies on a general pHMM built from the MSA of the currently known dimetal-carboxylate enzymes and the two motifs containing the catalytic Asp and Glu residues. As the ExxQExxH motif is missing in AurF [EC 1.14.99.68] (4-aminobenzoate N-oxygenase) and its homologues, the workflow can provide an indication of oxygenation activity as well. AurF did not match the halogenase pHMM during cross-validation; therefore, a cutoff is not necessary to differentiate it from the oxygenase homologues. However, searching for the conserved motif containing the active site can strengthen confidence in the categorization.

While a pHMM from copper-dependent ApnU homologues has been curated, we recommend relying on the two HXXHC motifs rather than homology-based annotation. The workflow can predict copper-dependent enzymes, but it is not suitable for distinguishing between halogenases and hydroxylases (SI). Due to the lack of experimental data about ApnU and the close homology with nonhalogenase members, only family-level annotation is recommended for this group. Moreover, while the two identical motifs have been reported in the halogenase sequence, motif similarity does not inherently mean identical function. Whether the HXXHC motifs directly contribute to the halogenation function needs further experimental studies in the copper-dependent family. Therefore, the application of this workflow is recommended if the goal is to identify copper-dependent enzymes or if we have a set of sequences for which we are aware of the halogenation function and want to assign the family to the enzymes.

The pipeline for FDHs starts with general family-level categorization as well. Known flavin-dependent monooxygenases and a dialkyldecalin synthase hit the general profile for FDHs with low bit scores. A phenol brominase, Bmp5 [EC 1.14.19.55], from pentabromopseudilin biosynthesis^53^ was not picked up during cross-validation, as this was the only representative of a single-component FDH with a GxGxxG motif but without the conventional WxWxI and Fx.PxSx:G motifs when building the general family-specific FDH model. However, the final model does include Bmp5 and therefore detects homologous sequences. The general model can also detect other single-component FDHs (e.g., VemK,^54^ PezW^55^ with the GxGxxG motif). Sequences with the GxGxxA motif did not hit the model, even though we trimmed the flavin-binding site from the MSA that we used to build the general FDHs pHMM. For the general FDH profile, we suggest relying on the conserved motifs instead of the bit score. The general model is suitable for identifying conventional members of the family with the Fx.PxSx:G motif and with or without the WxWxI Motif

Furthermore, there are substrate-specific models for tryptophan-, pyrrolic-, and phenol-acting halogenases. In the case of tryptophan-halogenases, the models differentiate between tryptophan-5 [EC 1.14.19.58] and tryptophan-6/7 halogenases [1.14.19.59/1.14.19.9]. While the tryptophan-5 profile does pick up MibH, a RiPP halogenase from the microbisporicin BGC,^56^ MibH-like sequences were underrepresented in the built model. Two separate pHMMs serve to differentiate between enzymes acting on tyrosine or orsellinic acid-like compounds. While these profiles work for the known sequences and their close homologues with the application of a bitscore cutoff (SI Evaluation Metric Chapter), the consideration of catalytic residues and conserved motifs is strongly recommended to make confident predictions. We recommend using the substrate-specific profile in combination with the sequence comparison feature, which allows users to determine whether catalytic residues from validated sequences are conserved in the query enzymes.

Similar to the previous groups, the VHPO workflow starts with a general model and offers halide-acceptance-specific categorization in the subsequent steps. These models follow the previously described naming conventions and do not predict halogen preference. While the profiles set apart known or canonical sequences, the sequence similarity networks (SSNs) (SI) showed unexplored spaces where a sequence hits multiple pHMMs. Furthermore, we try to give information about the selectivity of the enzyme if it matches the selective chloroperoxidase profile, by searching for the catalytic Lys and Ser residues described in NapH1 [1.11.1.10] from napyradiomycin biosynthesis.^17^ It also helps filter out NapH3- or MarH2-like sequences, which are close nonhalogenase VHPO homologues.^17^

Using pHMMs with the recommended cutoffs (Table 2) helps with strict categorization but is only suitable for canonical enzymes and might exclude unique sequences that still show halogenation activity or the same substrate specificity that the profiles, e.g., for tryptophan or pyrrole FDHs, indicate.

For large families, such as the FDHs and VHPOs, cutoffs help narrow down the pool of candidates for experimental work.

### Workflow of the Computational Pipeline

The computational workflow categorizes the sequence(s) of interest across the six mentioned enzyme families (Figure S4). The part of the pipeline for FDHs is integrated into antiSMASH 8.0 ^57^ while the complete analysis covering all six enzyme families is set up as a literate programming tool, implemented in a Python notebook, which enables the user to upload their sequence data in FASTA format, and guides them through the general categorization workflow (Figure 4; SI, Figures S3-S5).

**Figure 4:**
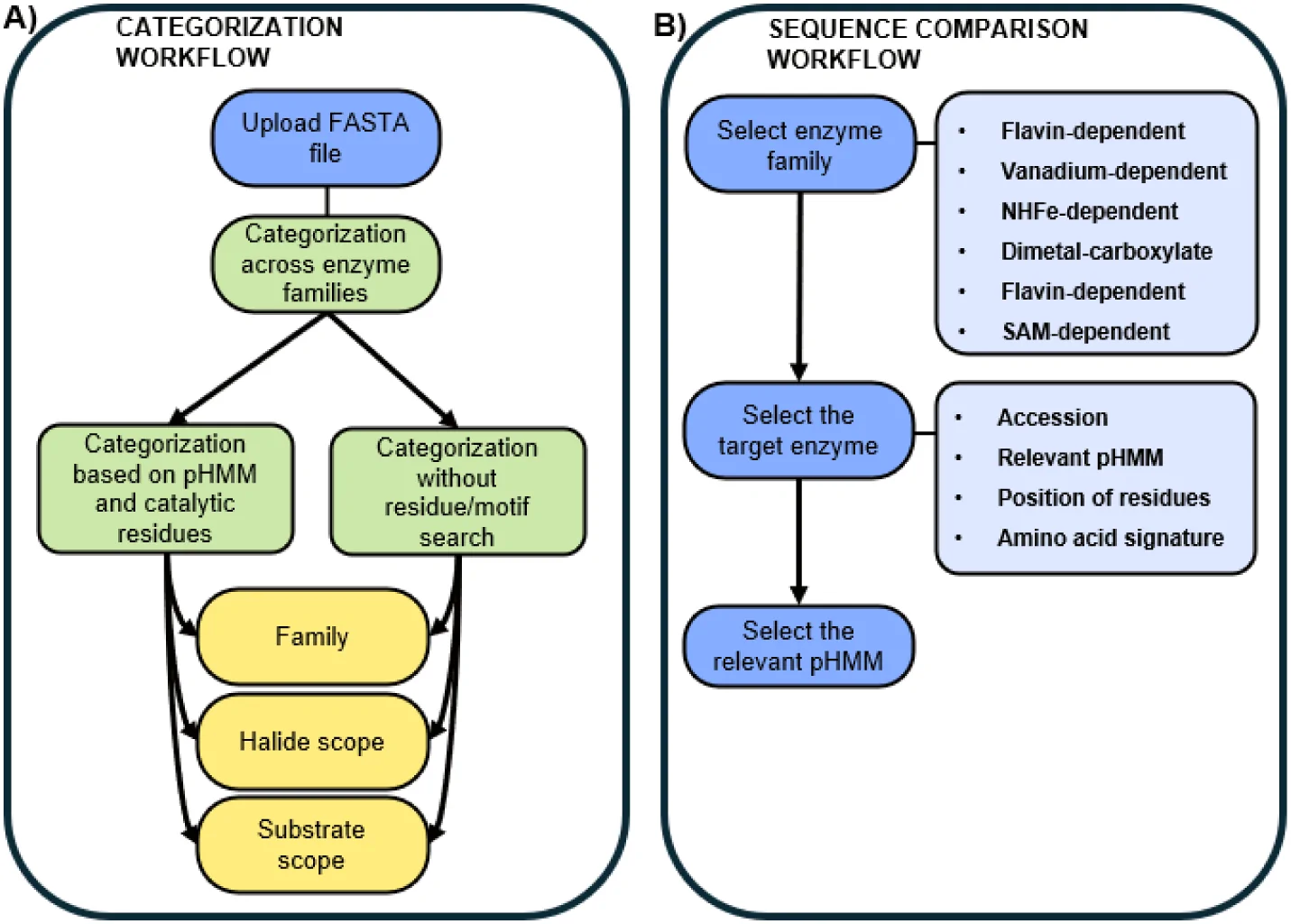
Process *of* categorization and sequence comparison. (A) Categorization of sequences with or without considering catalytic residues or conserved motifs. (B) Comparison of sequence(s) of interest to known target enzymes. The user needs to choose the halogenase family of interest, then select the experimentally validated halogenase that is used for comparison. Further details about the validated sequence can be viewed in the workflow.

Following the upload of the protein sequence(s) in a FASTA file, the user specifies the name of the file in a text field to initiate the analysis (SI, Figure S4). After filling out the text box and running the cell, the general categorization workflow can be executed (Figure S5). The output displays family-specific categorization results for each of the query sequences.

The categorization for each family branches into two paths (Figure 4A; SI, Figures S6–S11). It can be based on the presence of essential residues for the activity and conserved motifs or rely solely on the hit to certain pHMMs and the bitscore. The notebook showcases these two features. We indicate recommended bitscore cutoffs (Table 2), which were determined based on the sequence that had the lowest score when it was left out from building the model during the leave-one-out cross-validation. These cutoffs can be found in the notebook but are not automatically applied during the workflow. The code will return all hits (the names of proteins within the FASTA file) to the specific profile, and the user decides whether they consider the threshold.

Besides the categorization with or without considering functionally important amino acids, the workflow allows the comparison of sequence(s) to known, experimentally studied enzymes (Figure 4B). The target enzyme can be chosen from a dropdown list in the literate programming notebook (SI, Figure S13). Each target enzyme has a pHMM assigned, where the positions of catalytic residues are determined (SI, Figure S14). The information deposited in the Halogenase DB is leveraged in the section of the notebook where the user can search for catalytic residues and compare target sequences to known enzymes.

### Computational Workflow Highlights VHPO Diversity

We applied our computational pipeline, consisting of curated pHMMs, to UniRef50. Although the pHMMs were often dominated by specific taxa due to the available experimentally validated sequences (SF), analysis of UniRef50 showed that the models identified homologues across a wide range of phyla (SF) and revealed several unexplored groups across the covered halogenase families (SI UniRef50 Analysis Chapter). A cluster without validated sequences in the VHPO analysis was compelling for closer inspection. Among the VHPOs, the known selective chloroperoxidases formed their own cluster, which included several sequences with the catalytic Lys and functionally important Ser residues (Figure 5). Unconnected to this cluster, smaller groups were revealed by the selective VCPO profile, where the sequences also possess the conserved residues. Two other clusters without these amino acids fall between the clusters of known VBPOs and VCPOs. Close to them lies the VIPO cluster with the validated enzymes and several undescribed members containing the same catalytic residues as those of the iodoperoxidase from *Zobellia galactanivorans*. While they cluster tightly with the known sequences, they are connected to a separate part of the cluster that has no validated proteins yet. These catalytic residues are also present in two sequences in an unconnected group, which contains a VHPO from *Rhodopirellula (Rhoba*VHPO) (UniProt accession: Q7UVW2), previously described as a hypothetical VCPO, which clusters with VIPOs on the phylogenetic tree.^49^ The distance of *Rhoba*VHPO from the known VCPOs and the presence of putative VIPOs in the cluster called for experimental validation to confirm the halide scope.

**Figure 5:**
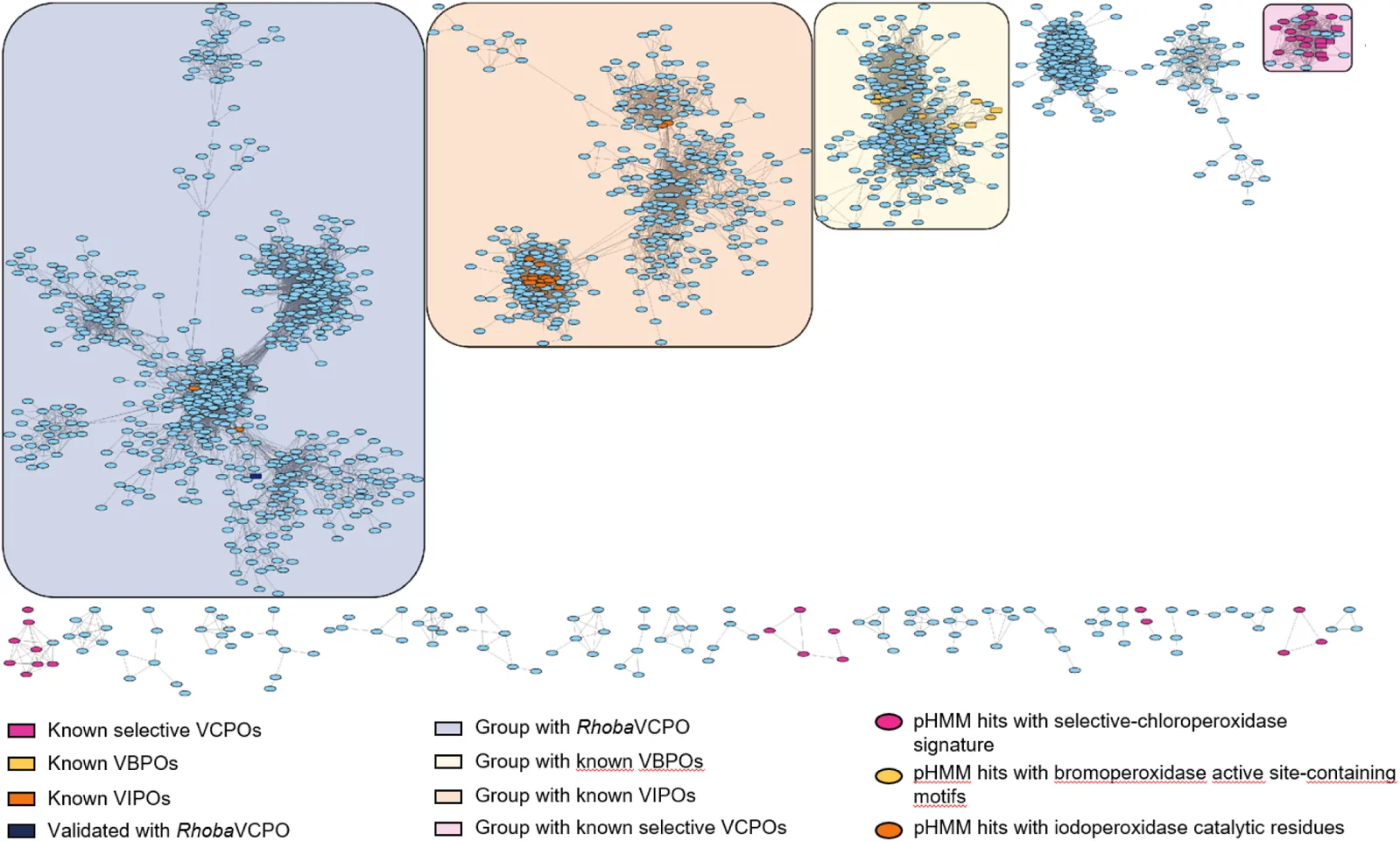
SSN of VHPOs. The built SSN contains hits from the selective and nonselective chloroperoxidase pHMMs, as well as from the bromoperoxidase and iodoperoxidase pHMMs, and reveals the space of VHPOs.

### VHPO from *Rhodopirellula* is a Chlorinase with Bromine Preference

While *Rhoba*VHPO was previously annotated as a hypothetical chloroperoxidase, no experimental study had confirmed this categorization or explored its halide scope.

We conducted the monochlorodimedone (MCD) assay (SI)^58^ to explore its chlorination and bromination potentials (Figure 6).

**Figure 6:**
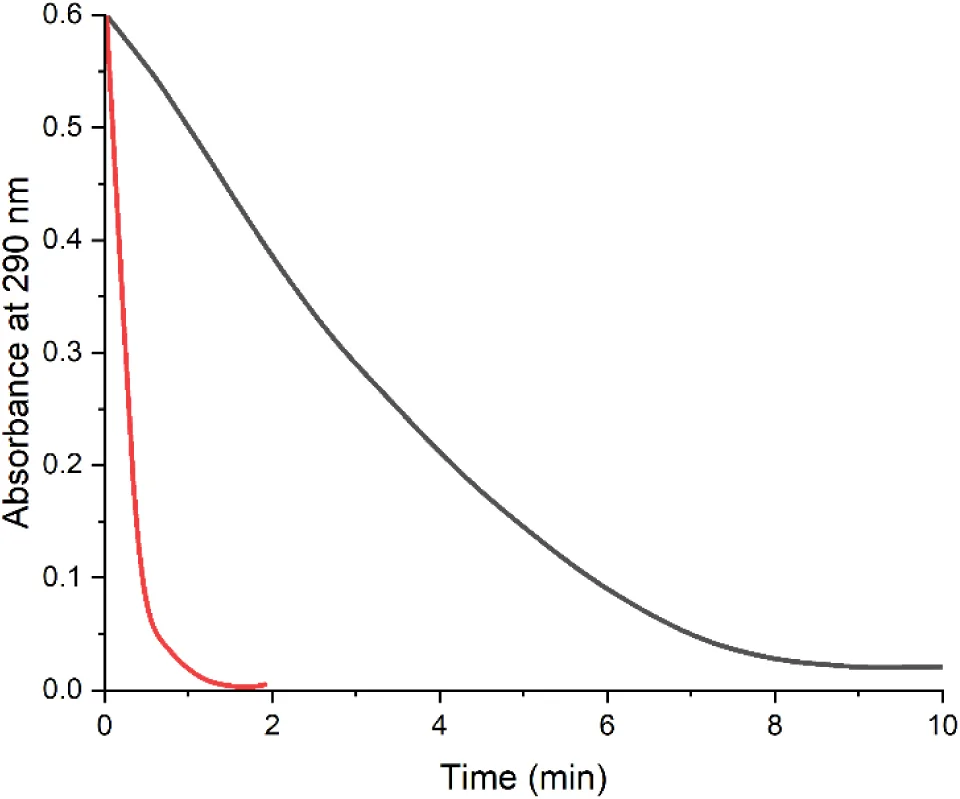
Absorbance trajectory of RhobaVHPO at 290 nm in the presence of KBr (red) and KCl (black).

Time course of MCD (μM) halogenation catalyzed by *Rhoba*VHPO (0.5 μM) in the presence of KBr (200 mM, red) and KCl (200 mM, black), Na_3_VO_4_ (10 μM) in 50 mM MES buffer, pH 6. The reactions were initiated by the addition of H_2_O_2_ (1 mM). Reactions were monitored by a decrease in absorbance at 290 nm. The rate of reaction was faster in the presence of KBr than KCl (Figure 6).

The MCD assay confirmed the chlorination activity and showed approximately a 5-fold greater preference for bromide over chloride under the assay conditions tested.

## Conclusion

This study provides a systematic framework to detect, categorize, and mine for novel halogenases across the SAM-dependent, NHFe-dependent, dimetal-carboxylate, copper-dependent, flavin-dependent, and vanadium-dependent families. Currently, known enzymes are collected in a database with information on halide acceptance, mutagenesis studies, and structural analysis, as well as the positions of catalytic residues in the corresponding pHMMs.

The collected data on functionally important residues is leveraged in a pHMM-based computational pipeline to detect halogenases from the above-listed families and to provide an indication of their halide and/or substrate scope. While sequence homology tools, such as BLAST,^37^ HHPred,^39^ or InterPro,^40^ have been used for decades for enzyme annotation, our developed resource contains, to our knowledge, the first pipeline that specifically targets halogenating enzymes across six enzyme families and searches for essential amino acid motifs. These motifs can be used in the comparison part of the pipeline to gain further confidence about halogenation activity and substrate specificity. In the case of FDHs, WxWxI­(P) indicates the lack of monooxygenation activity. However, the pipeline targets predicting halogenation function, and while the summary in the Python notebook and in the Guide for the Residue and Motif Collection in the SI informs us which activities could be present or could be missing from the enzyme’s repertoire, the developed computational resource does not address multifunctional proteins. An example of this is the NHFe-dependent halogenase group, where the catalytic triad indicates halogenation activity, but the enzyme could also showcase dioxygenation or hydroxylation. Moreover, the level of prediction greatly depends on the available experimental information. While e.g., for example, in the case of FDHs, the important residues for tetra-or di/monohalogenation were established through experiments, such studies do not exist for every halogenase group.

Although catalytic residues strengthen the annotation, searching for small motifs (e.g., HXA/G in NHFe-dependent homologues) can result in false positives. This problem is mitigated by restricting the search area in the pHMM alignment; however, it raises another issue. Sequences with deletions or insertions around the searched sequence regions may be discarded by the workflow. When the goal of annotation is to find novelty, it is recommended that one search a larger region in the sequence to avoid missed hits. Whereas, if the aim is confident classification, the trade-off of a narrowed search area and missed hits might be worth taking. In either case, using recommended cutoffs is optional, and the user can view hits with and without requiring the functionally important residues.

Moreover, running the general workflow on UniRef50 helped us detect several promising candidates for halogenation, supported by the presence of conserved motifs and catalytic amino acids. The SSNs show underexplored clusters across all of the discussed enzyme families containing putative halogenases (SI). We emphasized the VHPO SSN due to an unexplored cluster closely located near iodoperoxidases. We confirmed the chlorinating activity of a member of this cluster from *Rhodopirellula baltica*, previously predicted as a chloroperoxidase, which allowed us to curate a pHMM specific to this cluster, with indications of activity in the computational pipeline. The curated model facilitates the discovery of further members of the cluster and the exploration of novelty among VHPOs.

The goal of the computational resources was to maximize the accessibility and transparency of both the underlying information and the prediction. The currently most accessible medium for protein analysis is the sequences, as the crystal structures are not always available. While the prediction of protein structure has been made easier through web tools, its scalability is still limited by computational hardware. Relying on protein sequences and the positional independence that pHMM assumes misses important structural and functional information. As structure prediction tools become more accurate and accessible, structure-aware models might complement or replace solely sequence-based annotation in the future.

## Materials and Methods

### Halogenase DB

The literature search relied on a thorough review paper covering the FDHs, SAM-dependent enzymes, and metalloenzyme families.^3^ The study of the review was followed by a primary focus on research papers and rigorous manual curation of experimental information across the SAM-dependent, NHFe-dependent, dimetal carboxylate, FDH, and VHPO families. The curation was further aided by the search for validated enzymes across the six families in SwissProt, which accelerated the search process and allowed us to focus on the results of substrate testing or structural analysis.

A PostgreSQL database was developed to store the results of the literature search, which was later converted to JSON for easier accessibility. The database stores data about enzyme name, accession/availability, producing organism, type of validation, halide acceptance, and references. If information on mutagenesis and structure analysis studies was available, it was deposited in a table specific to that enzyme. Minimal information about the protocol was also noted.

We collected the so-far experimentally validated or *in silico*-inferred catalytic residues in the database to aid in proper annotation. The database includes the catalytic residues and their roles, as well as how the mutation of those residues affected enzyme activity in mutagenesis studies.

HMMEditor^59^ was used to determine the index of the catalytic residue in the given pHMM.

### pHMM Curation and Testing

pHMMs were built from multiple sequence alignments (MSA), and in the case of general, tyrosine/Hpg-like, and pyrrolic FDH profiles for NHFe-dependent nucleotide pHMM and VCPO pHMM, the MSAs were trimmed to exclude highly variable terminal regions. For FDHs, the sequences were often trimmed at the N- and C-terminal regions if they showed high variability. As the low-homology regions at the terminals were excluded, MUSCLE was suitable for MSA generation. In the case of the SAM-dependent and metal-dependent families, the pHMMs were often built from whole sequence alignments (Table 2). Therefore, we used MAFFT for SAM-dependent, NHFe-dependent, dimetal-carboxylate halogenases, and VHPOs, an alignment algorithm that is more suitable to handle low-homology N-terminal or C-terminal extensions.^60,61^

Sequences were retrieved from the study that described them, UniProt, or NCBI.^62^

### SSN Curation

UniRef50 was searched with HMMER^63^ against the curated pHMMs without thresholds. We added the known enzymes used for building the pHMMs to each set of sequences. The resulting FASTA files were the input for the Enzyme Function Initiative-Enzyme Similarity Tool (EFI-EST)^64,65^ to generate the SSNs. We visualized the generated SSN with Cytoscape 3.10.1 ^66^ using the default E-value for edge calculation (negative log of 5). The sequence alignment threshold parameter in EFI-EST^64,65^ was modified with consideration of the relationship between the value of the sequence alignment score and the percent identity. The cutoff was chosen at a value where enzymes with similar halide or substrate scope clustered together and were separated from their closest homologues with different activity.

Geneious Prime 2024.03.25 was used to retrieve the domain composition data for the sequences.

### MCD Assay of VHPO *Rhodopirellula baltica*

All chemicals and biological materials were obtained from commercial suppliers (SI). The assay followed the protocol described by McKinnie, Miles, and Moore.^58^

### Construction of pET28a­(+)_*Rhoba* VHPO

The *E. coli*-optimized gene (synthesized by Twist) for VHPOs from *Rhodopirellula baltica* was cloned into pET28a­(+) using *NdeI* and *XhoI* restriction sites to yield pET28a­(+)_VHPO_Rhoba.

### Protein Production and Purification

For the expression of *Rhoba*VHPO, chemically competent *E. coli* BL21 (DE3) cells were transformed with the relevant pET28a­(+)_VHPO construct. Single colonies of freshly transformed cells were cultured for 18 h in 5 mL LB medium containing 50 μg mL^–1^ kanamycin. Starter cultures (5 mL) were used to inoculate 500 mL TB medium supplemented with 50 μg mL^–1^ kanamycin. Cultures were grown at 37 °C and 200 rpm to an optical density at 600 nm (OD600) of around 0.5. Protein expression was induced with the addition of IPTG to a final concentration of 0.1 mM. Induced cultures were incubated for 20 h at 18 °C, and the cells were subsequently collected by centrifugation (4,000 rpm for 30 min). Pelleted cells were resuspended in a lysis buffer (50 mM HEPES, 300 mM NaCl, pH 7.5, containing 20 mM imidazole) and lysed by sonication. Cell lysates were cleared by centrifugation (10,000 rpm for 30 min), and the supernatants were subjected to affinity chromatography using Ni-NTA Agarose (Qiagen). Purified protein was eluted using 50 mM HEPES, 300 mM NaCl, pH 7.5, containing 250 mM imidazole. The protein was desalted using 10DG desalting columns (Bio-Rad) with 50 mM MES, pH 6, and analyzed by SDS-PAGE. The protein was aliquoted, flash-frozen in liquid nitrogen, and stored at −80 °C. Protein concentration was determined by measuring the absorbance at 280 nm and assuming an extinction coefficient of 76,320 M^–1^ cm^–1^.

### Monochlorodimedone (MCD) Assay

Assays were performed in a quartz cuvette using a Cary 50 Bio UV–vis spectrophotometer, measuring absorbance at 290 nm. MCD (50 μM), KCl or KBr (200 mM), Na_3_VO_4_ (10 μM) and purified *Rhoba*VHPO (0.5 μM) in 50 mM MES, pH 6, were mixed in a cuvette and measured for 1 min. After 1 min, the reaction was initiated by the addition of H_2_O_2_ (1 mM). The total reaction volume was 1 mL. Data were processed and plotted in OriginPro 2025.

## Supplementary Material





## Data Availability

Notebook: https://colab.research.google.com/drive/1imau-B7BDzHzTjVrzo6vXCOxtDUcKnq7?usp=sharing. Hal-miner GitHub: https://github.com/JudSze/hal_miner. Halogenase DB: https://halogenases.secondarymetabolites.org/. Github of pHMM curation and testing: https://github.com/JudSze/hal_miner_curation. SSNs: 10.5281/zenodo.18377887

## Notes

SSNs and summary for the covered enzyme families and NHFe-dependent family phylogeny. Supplementary figures : Parametrization and analysis of SSNs for the covered enzyme families and NHFe-dependent family phylogeny (PDF)
Monochlorodimedone assay; Sequence similarity networks (PDF)
