# Supplementary material for: Computational Pipeline Reveals Nature’s Untapped Reservoir of Halogenating Enzymes
Source: ACS Omega. 2026 Aug 11;11(33):49420–31. doi: 10.1021/acsomega.6c02136 (PMC13519999; doi:10.1021/acsomega.6c02136)
Supplement: Supplementary file 2 [file ao6c02136_si_002.pdf]

Monochlorodimedone (MCD) assay

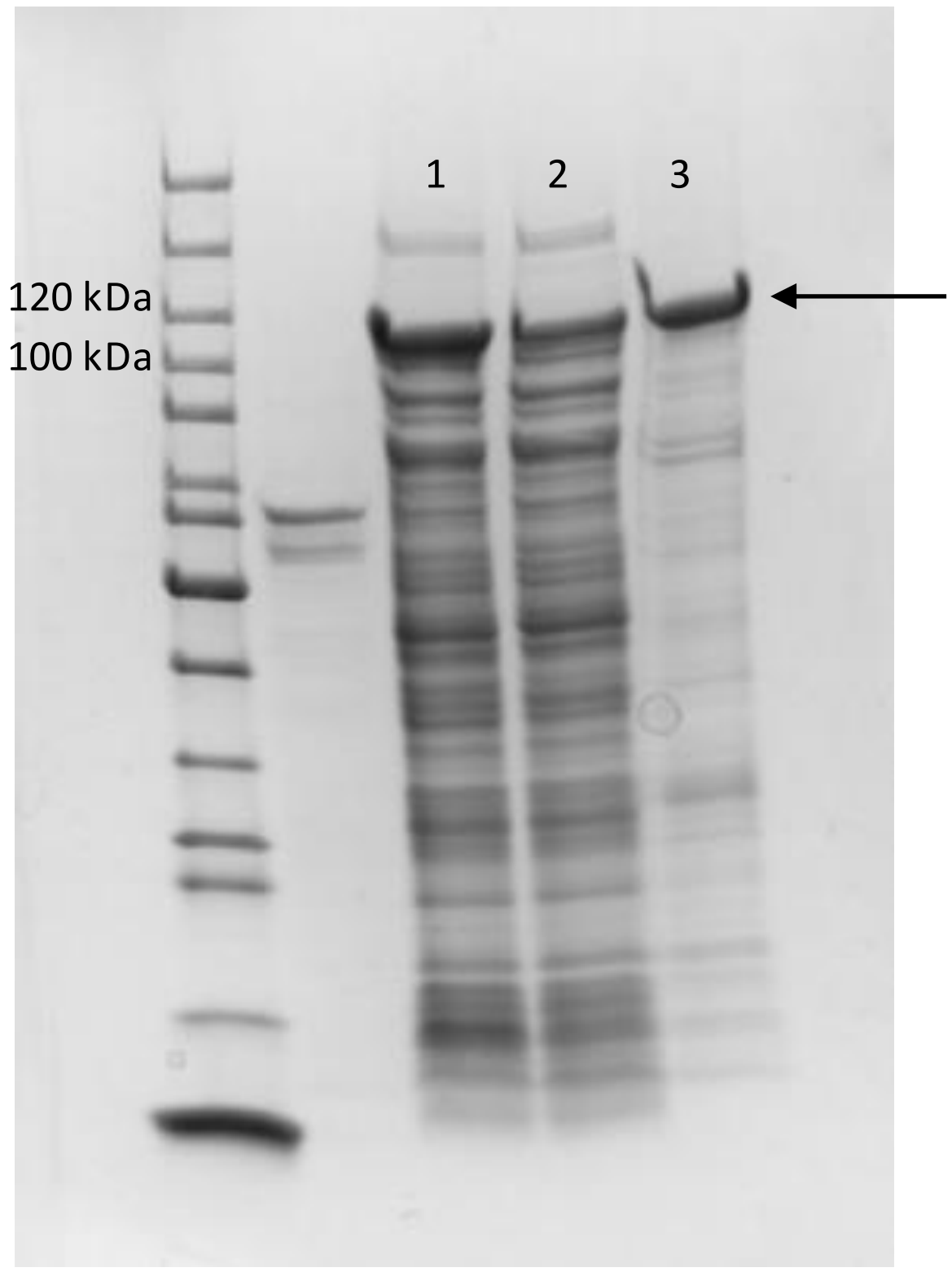

1- Total proteins
2- Soluble proteins
3- Purified fraction

Expected size of his-tagged protein 78.7 kDa

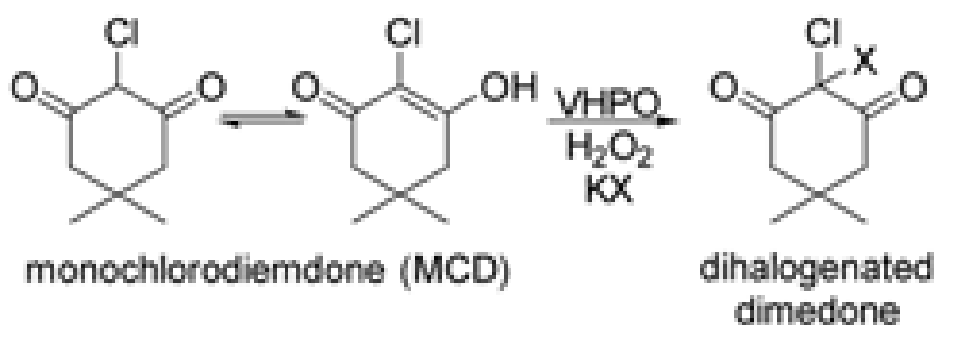

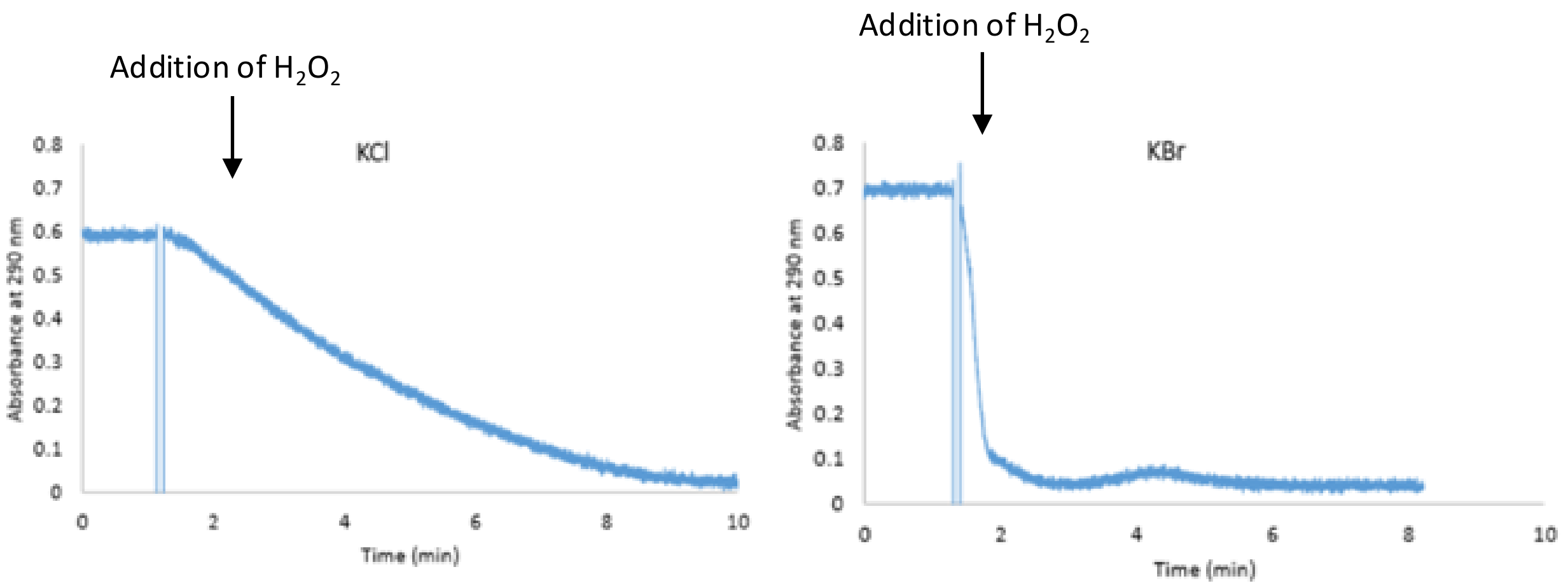

Assay conditions: 50 uM MCD, 200 mM KBr or KCl, 10 uM Na_3VO_4, 0.5 uM Enzyme, Buffer: 50 mM MES pH=6. Initiate reaction with 1 mM H_2O_2.

Sequence Similarity Networks

Vanadium-dependent

Job Number	156836
Time Started -- Finished	7/2 06:54 AM -- 7/2 07:00 AM
Database Version	UniProt: 2025-02 / InterPro: 105
Input Option	FASTA (Option C), no FASTA header reading
Job Name	VHPOs
Input Sequence Source	UniRef50
E-Value for SSN Edge Calculation	5
Uploaded FASTA File	ssn.fasta
Number of Sequences in Uploaded File	3,654
Exclude Fragments	No
Total Number of Sequences in Dataset	3,654
Total Number of Edges	1,640,171
Number of Unique Sequences	3,650
Convergence Ratio?	0.246

Alignment Score threshold: 80

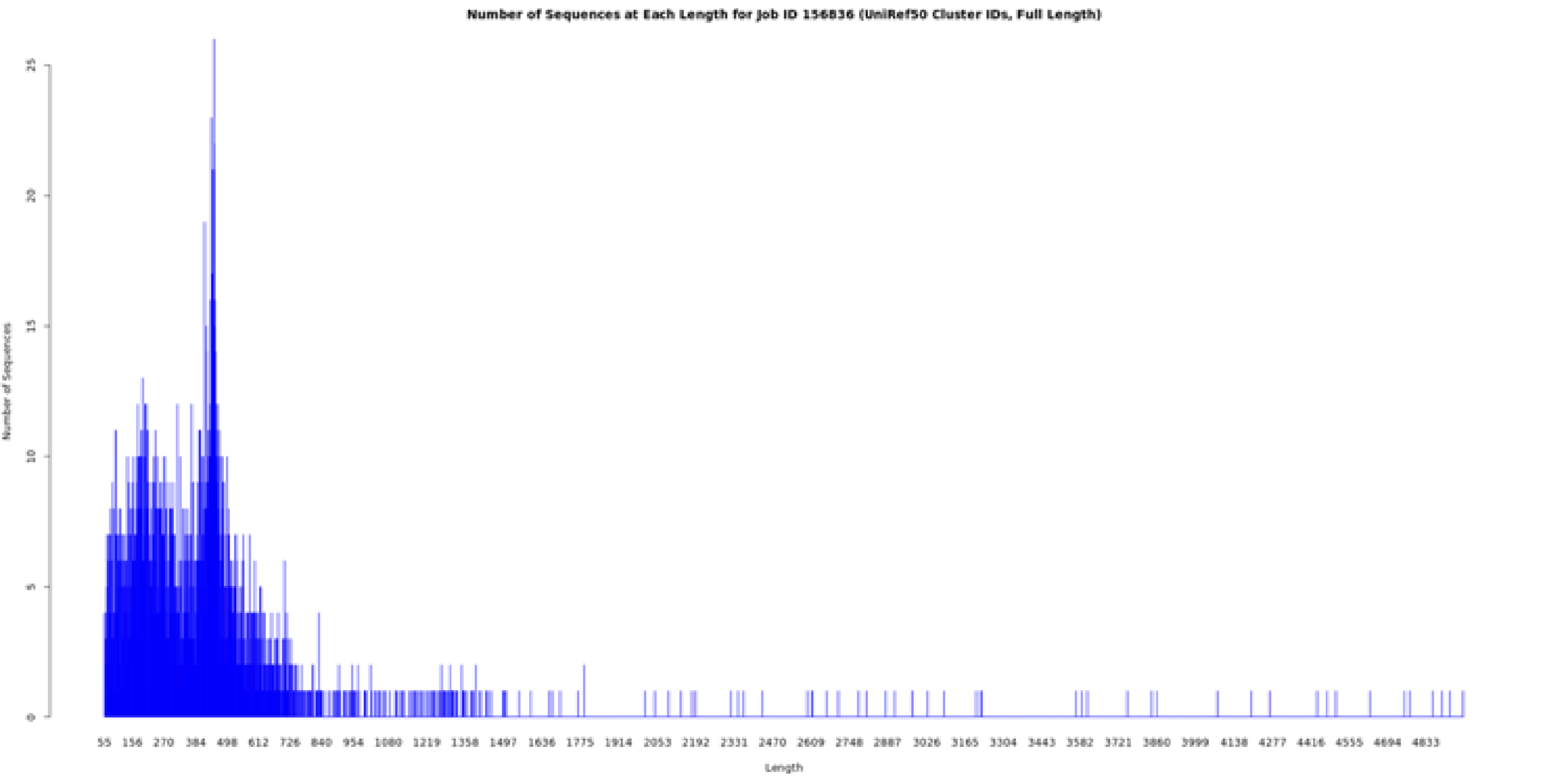

Number of Sequences at Each Length for Job ID 156836 (UniRef50 Cluster IDs, Full Length)
Number of Sequences
0
5
10
15
20
25
55 156 270 384 498 612 726 840 954 1080 1219 1358 1497 1636 1775 1914 2053 2192 2331 2470 2609 2748 2887 3026 3165 3304 3443 3582 3721 3860 3999 4138 4277 4416 4555 4694 4833
Length

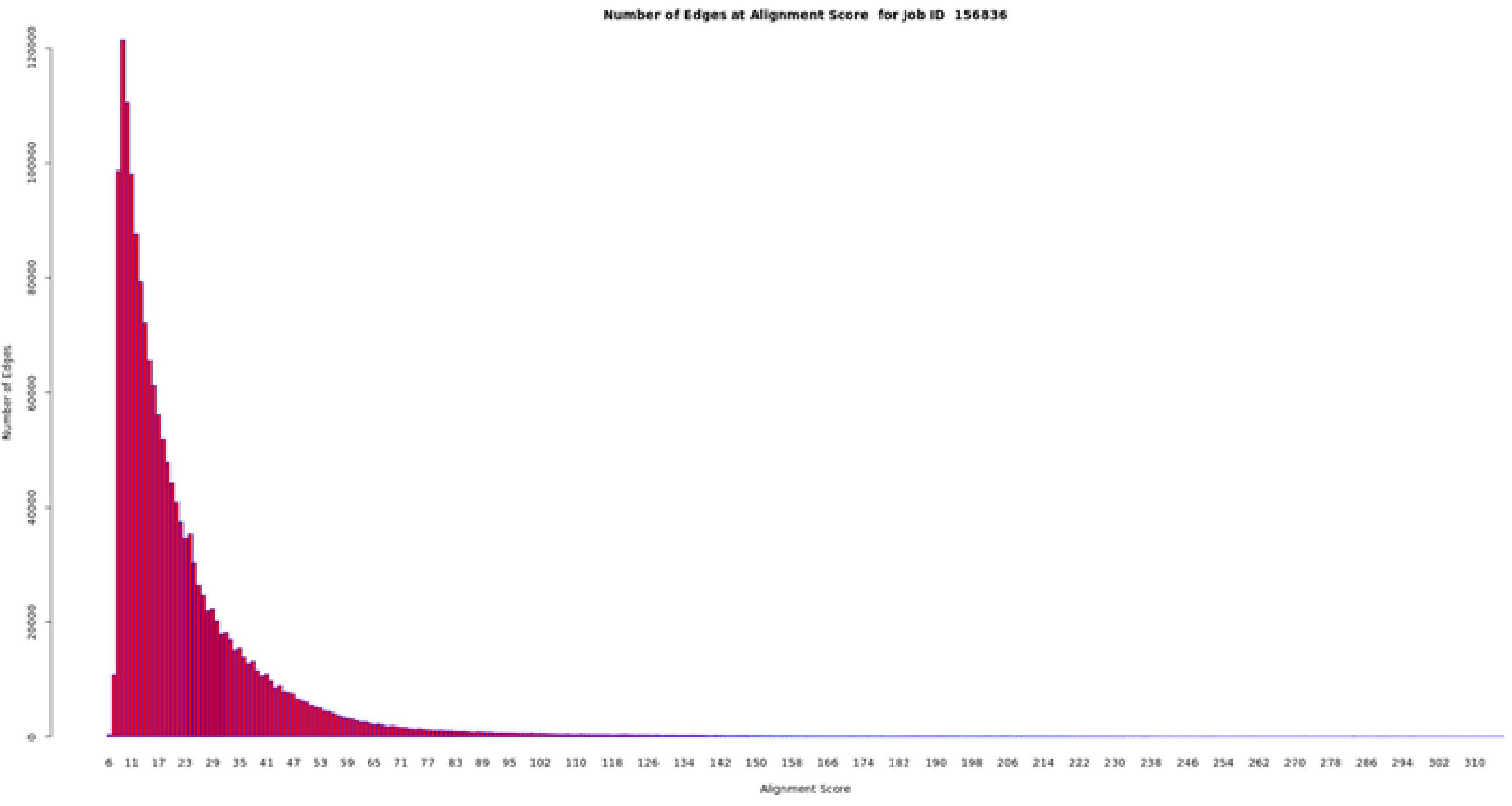
Number of Edges at Alignment Score for Job ID 156836
Number of Edges
Alignment Score
0
20000
40000
60000
80000
100000
120000
6
11
17
23
29
35
41
47
53
59
65
71
77
83
89
95
102
110
118
126
134
142
150
158
166
174
182
190
198
206
214
222
230
238
246
254
262
270
278
286
294
302
310

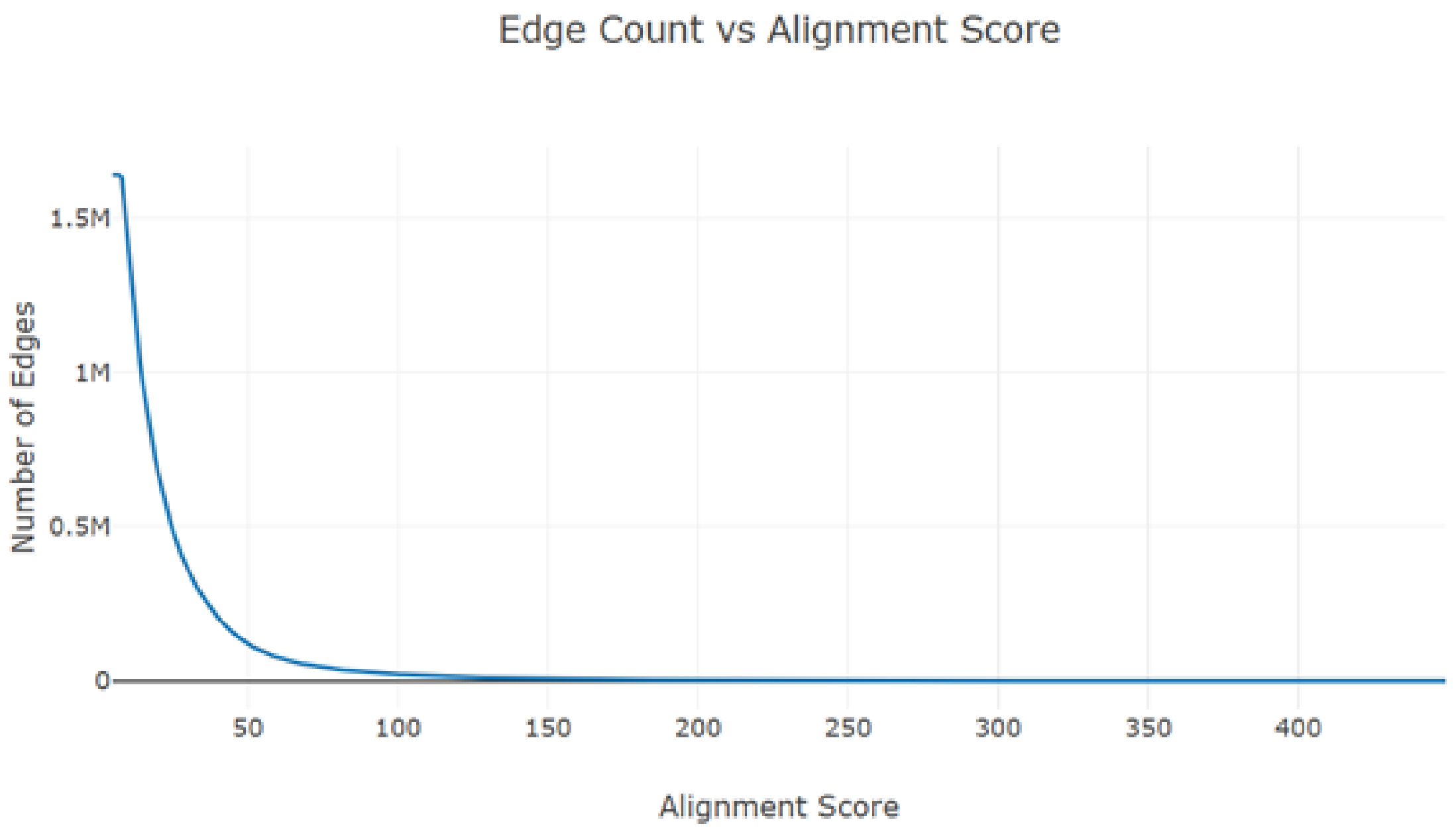
Edge Count vs Alignment Score
Number of Edges
1.5M
1M
0.5M
0
50
100
150
200
250
300
350
400
Alignment Score

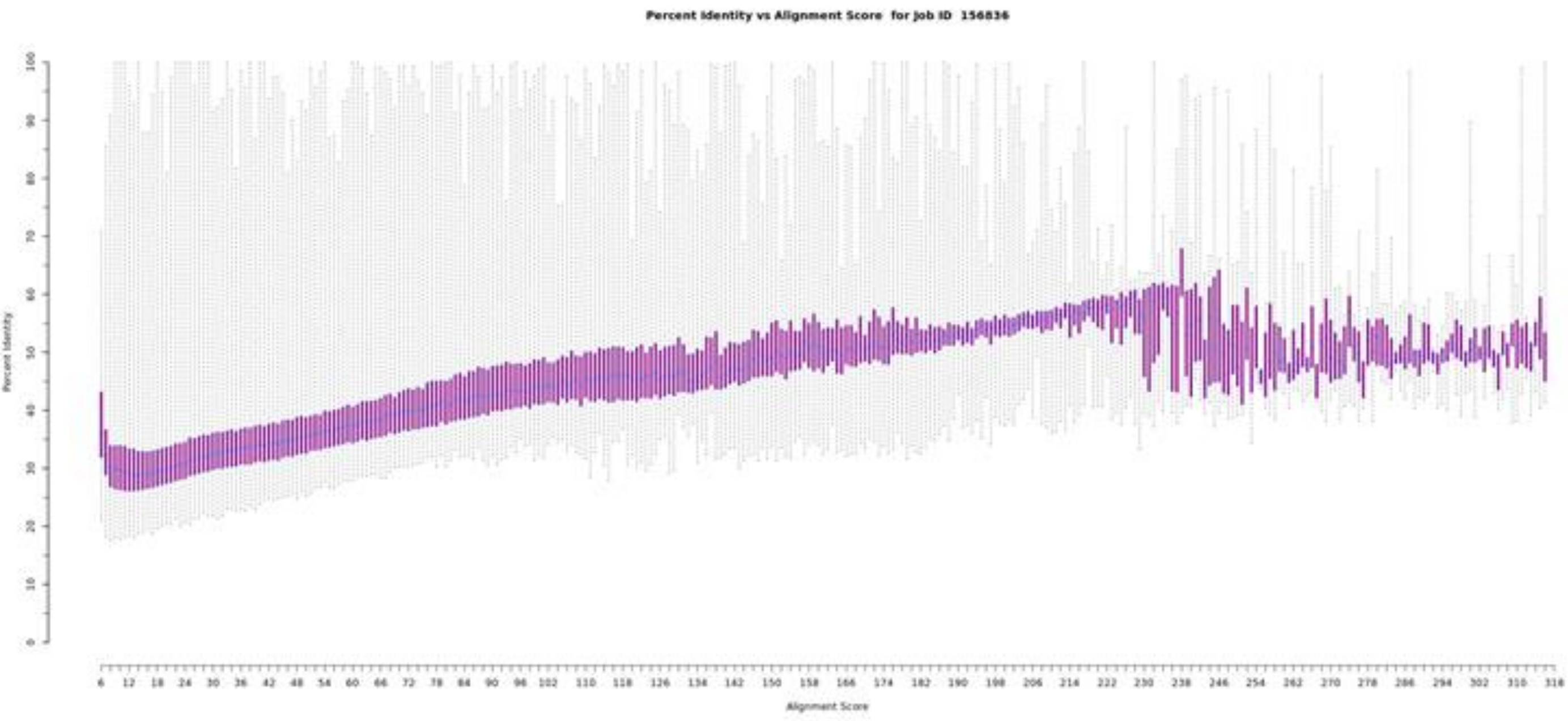
Percent Identity vs Alignment Score for Job ID 156836
Percent Identity
Alignment Score

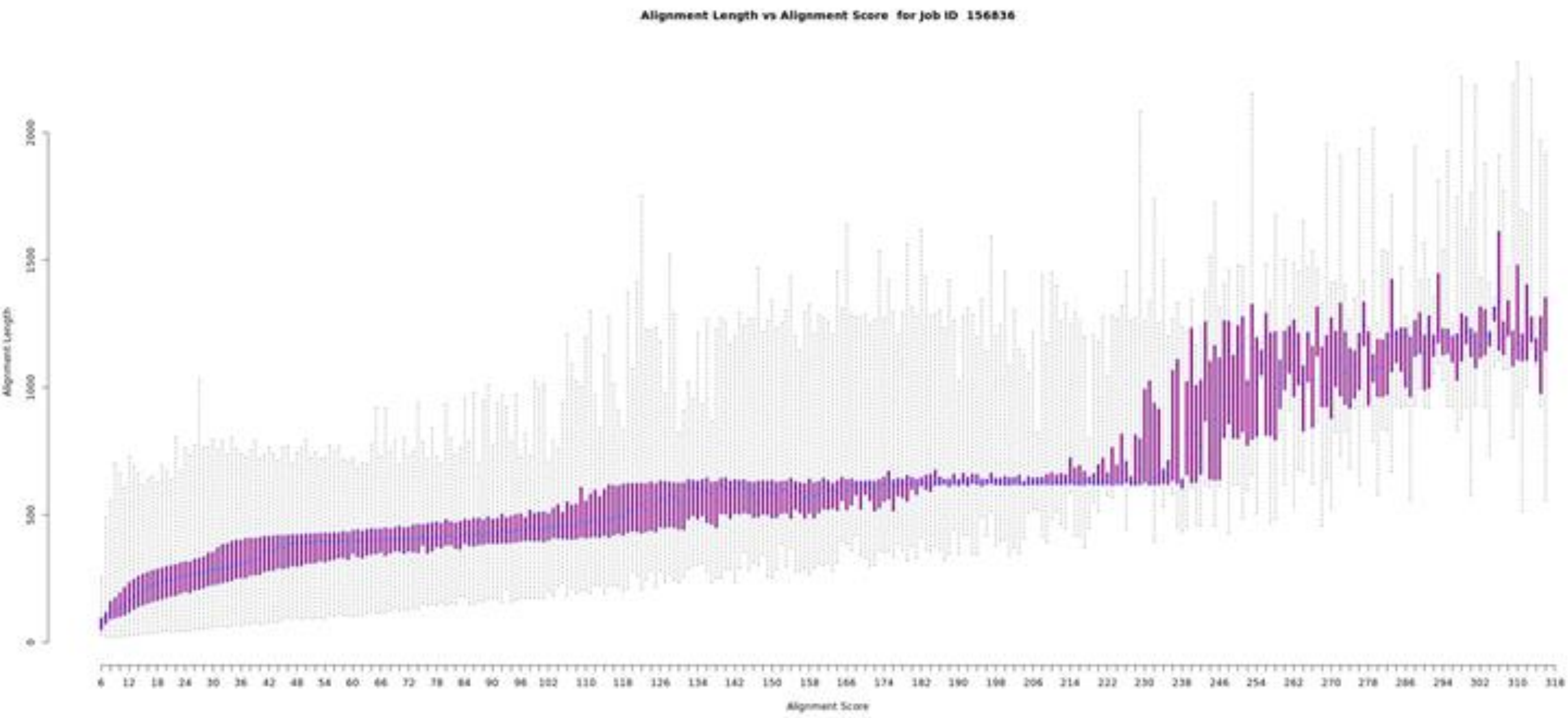
Alignment Length vs Alignment Score for Job ID 156836
Alignment Length
Alignment Score
0
500
1000
1500
2000
6
12
18
24
30
36
42
48
54
60
66
72
78
84
90
96
102
110
118
126
134
142
150
158
166
174
182
190
198
206
214
222
230
238
246
254
262
270
278
286
294
302
310
318

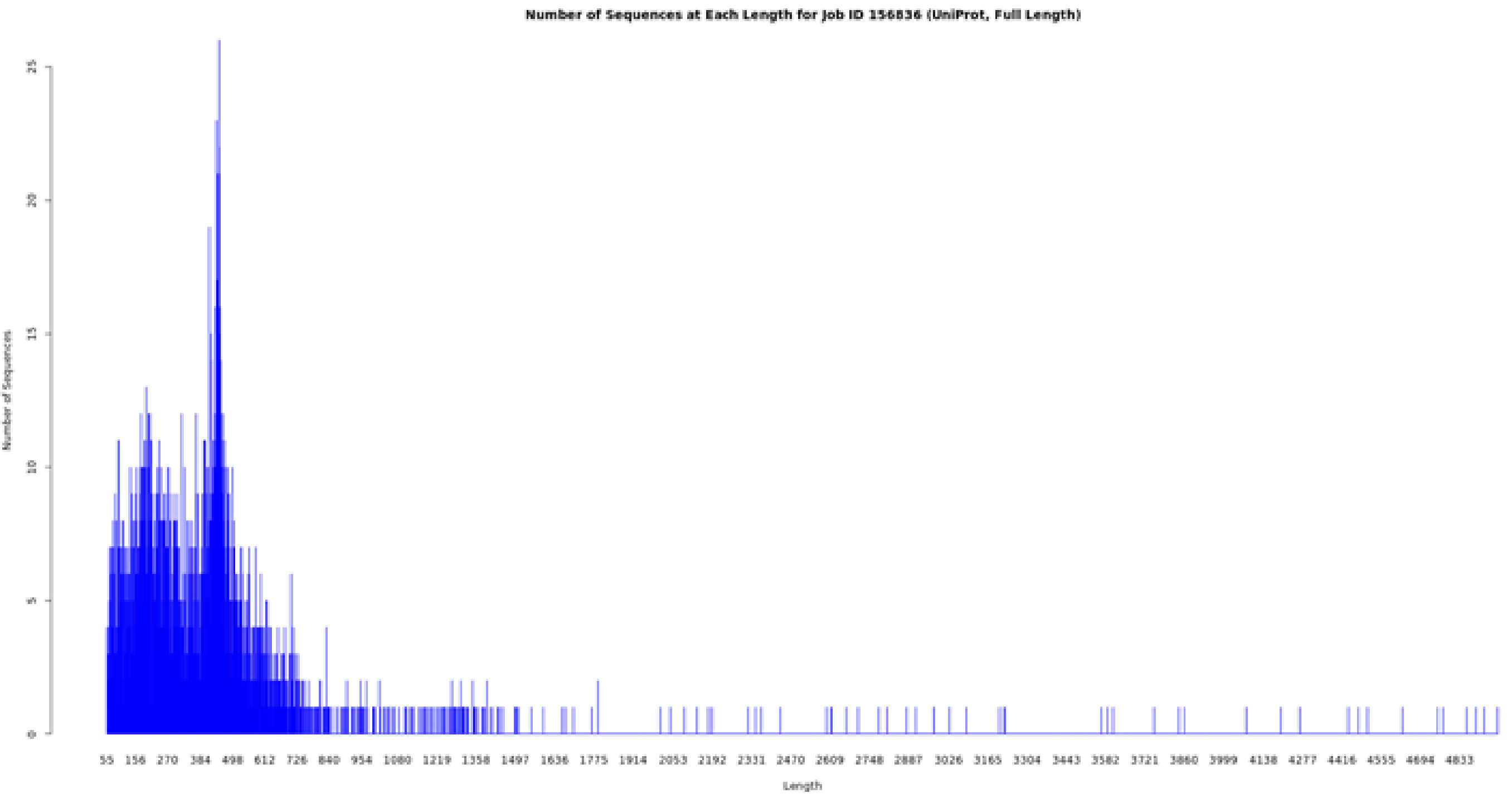

Number of Sequences at Each Length for Job ID 156836 (UniProt, Full Length)
Number of Sequences
0
5
10
15
20
25
55 156 270 384 498 612 726 840 954 1080 1219 1358 1497 1636 1775 1914 2053 2192 2331 2470 2609 2748 2887 3026 3165 3304 3443 3582 3721 3860 3999 4138 4277 4416 4555 4694 4833
Length

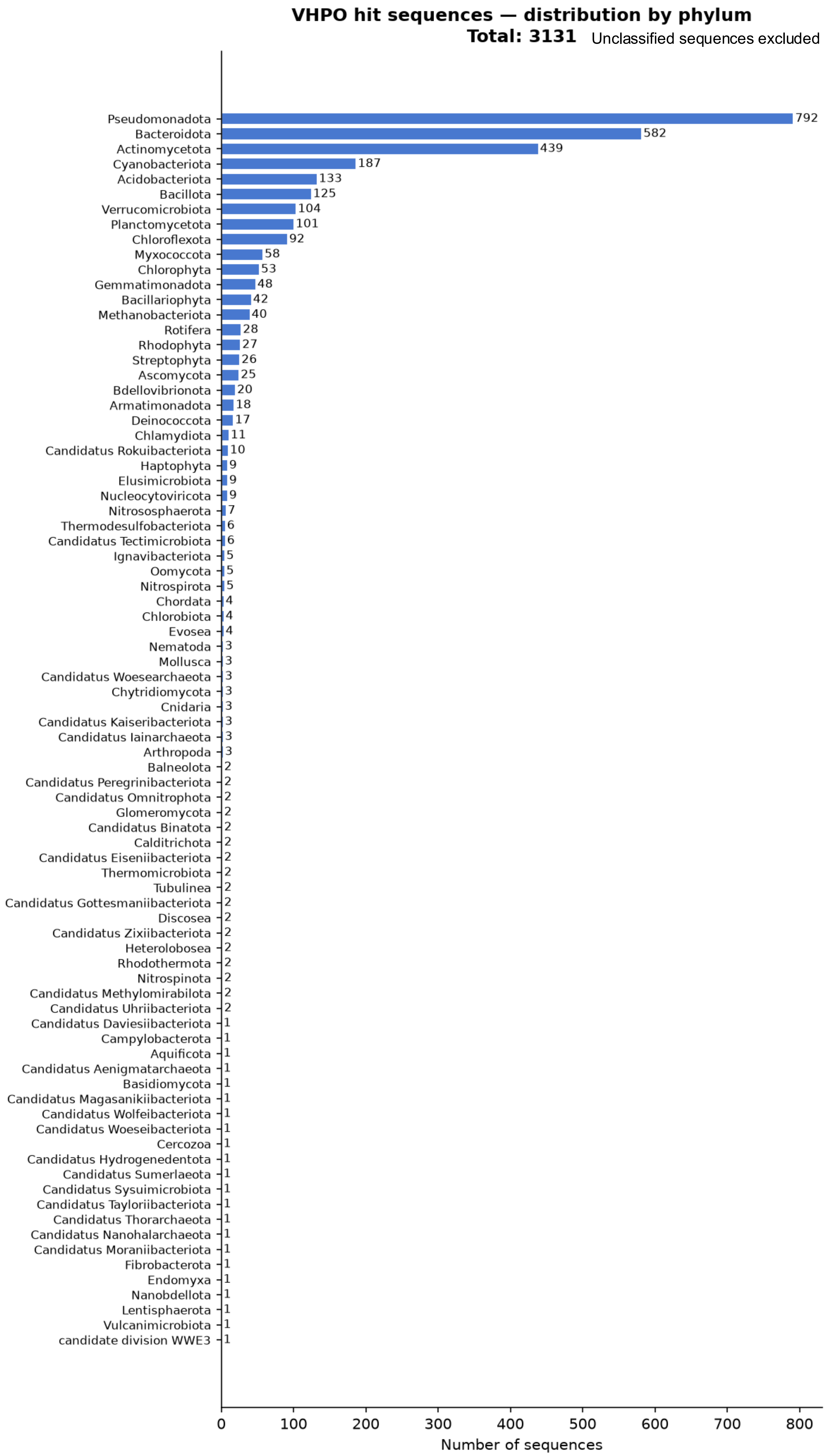
VHPO hit sequences — distribution by phylum
Total: 3131
Unclassified sequences excluded
Pseudomonadota 792
Bacteroidota 582
Actinomycetota 439
Cyanobacteriota 187
Acidobacteriota 133
Bacillota 125
Verrucomicrobiota 104
Planctomycetota 101
Chloroflexota 92
Myxococcota 58
Chlorophyta 53
Gemmatimonadota 48
Bacillariophyta 42
Methanobacteriota 40
Rotifera 28
Rhodophyta 27
Streptophyta 26
Ascomycota 25
Bdellovibrionota 20
Armatimonadota 18
Deinococcota 17
Chlamydiota 11
Candidatus Rokuibacteriota 10
Haptophyta 9
Elusimicrobiota 9
Nucleocytoviricota 9
Nitrososphaerota 7
Thermodesulfobacteriota 6
Candidatus Tectimicrobiota 6
Ignavibacteriota 5
Oomycota 5
Nitrospirota 5
Chordata 4
Chlorobiota 4
Evosea 4
Nematoda 3
Mollusca 3
Candidatus Woesearchaeota 3
Chytridiomycota 3
Cnidaria 3
Candidatus Kaiseribacteriota 3
Candidatus Iainarchaeota 3
Arthropoda 3
Balneolota 2
Candidatus Peregrinibacteriota 2
Candidatus Omnitrophota 2
Glomeromycota 2
Candidatus Binatota 2
Calditrichota 2
Candidatus Eiseniibacteriota 2
Thermomicrobiota 2
Tubulinea 2
Candidatus Gottesmaniibacteriota 2
Discosea 2
Candidatus Zixiibacteriota 2
Heterolobosea 2
Rhodothermota 2
Nitrospinota 2
Candidatus Methylomirabilota 2
Candidatus Uhriibacteriota 2
Candidatus Daviesiibacteriota 1
Campylobacterota 1
Aquificota 1
Candidatus Aenigmatarchaeota 1
Basidiomycota 1
Candidatus Magasanikiibacteriota 1
Candidatus Wolfeibacteriota 1
Candidatus Woeseibacteriota 1
Cercozoa 1
Candidatus Hydrogenedentota 1
Candidatus Sumerlaeota 1
Candidatus Sysuimicrobiota 1
Candidatus Tayloriibacteriota 1
Candidatus Thorarchaeota 1
Candidatus Nanohalarchaeota 1
Candidatus Moraniibacteriota 1
Fibrobacterota 1
Endomyxa 1
Nanobdellota 1
Lentisphaerota 1
Vulcanimicrobiota 1
candidate division WWE3 1
0
100
200
300
400
500
600
700
800
Number of sequences

Dimetal-carboxylate

Job Number	156847
Time Started -- Finished	7/2 10:00 AM -- 7/2 10:03 AM
Database Version	UniProt: 2025-02 / InterPro: 105
Input Option	FASTA (Option C), no FASTA header reading
Job Name	5-dimetal-carboxylate
E-Value for SSN Edge Calculation	5
Uploaded FASTA File	dimetal.fasta
Number of Sequences in Uploaded File	88
Exclude Fragments	No
Total Number of Sequences in Dataset	88
Total Number of Edges	969
Number of Unique Sequences	88
Convergence Ratio?	0.253

Alignment Score threshold: 51

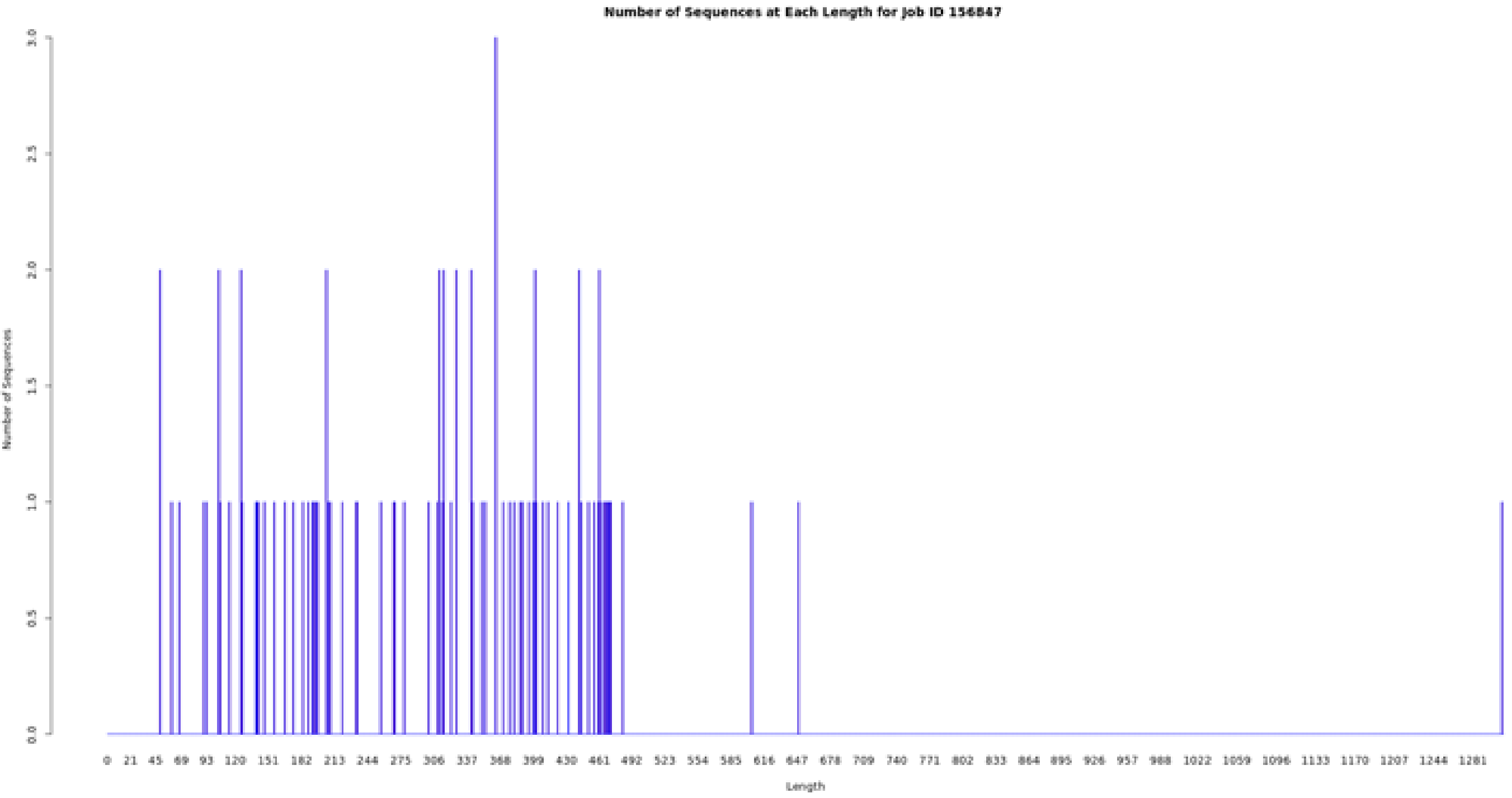
Number of Sequences at Each Length for Job ID 156847
Number of Sequences
Length
0.0
0.5
1.0
1.5
2.0
2.5
3.0
0 21 45 69 93 120 151 182 213 244 275 306 337 368 399 430 461 492 523 554 585 616 647 678 709 740 771 802 833 864 895 926 957 988 1022 1059 1096 1133 1170 1207 1244 1281

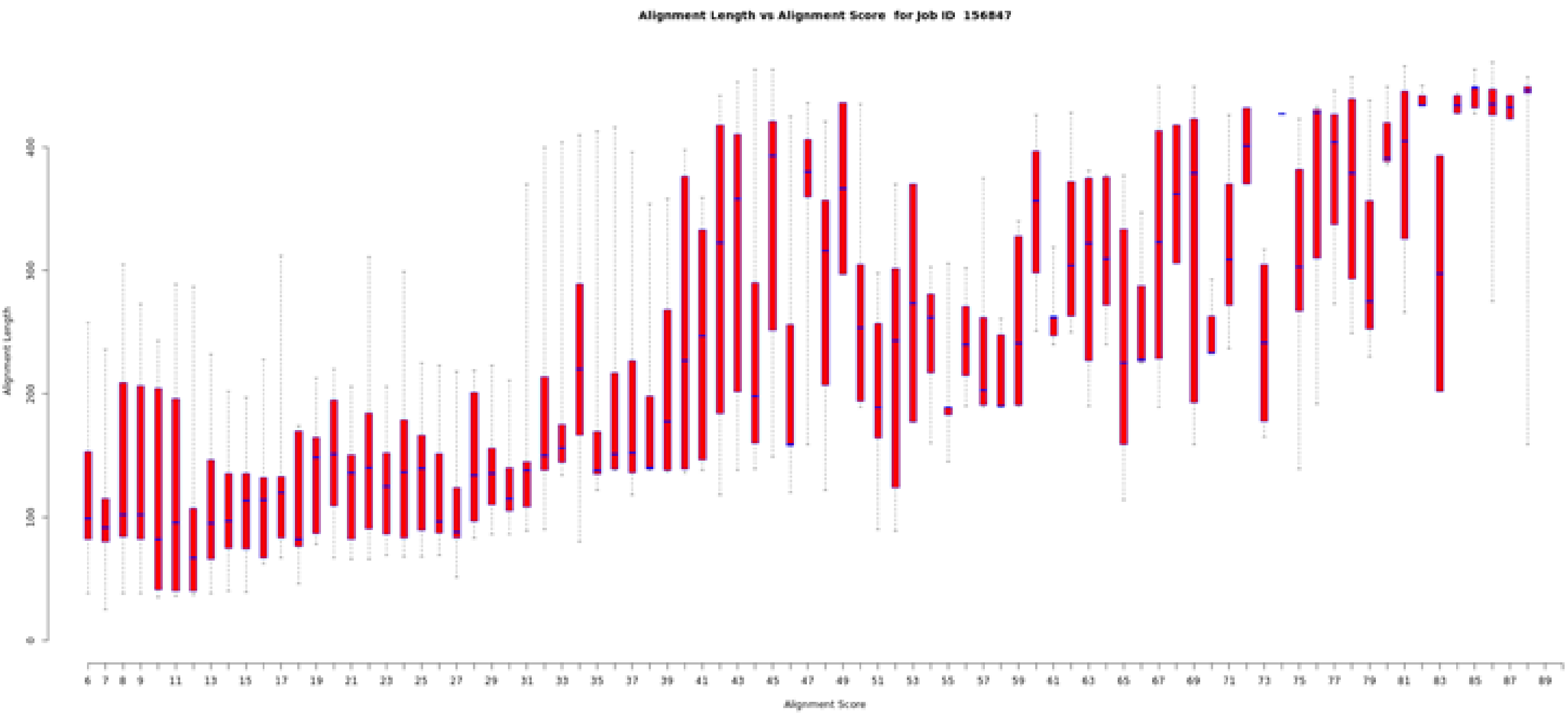

Alignment Length vs Alignment Score for Job ID 156847
Alignment Length
Alignment Score
0
100
200
300
400
6 7 8 9 11 13 15 17 19 21 23 25 27 29 31 33 35 37 39 41 43 45 47 49 51 53 55 57 59 61 63 65 67 69 71 73 75 77 79 81 83 85 87 89

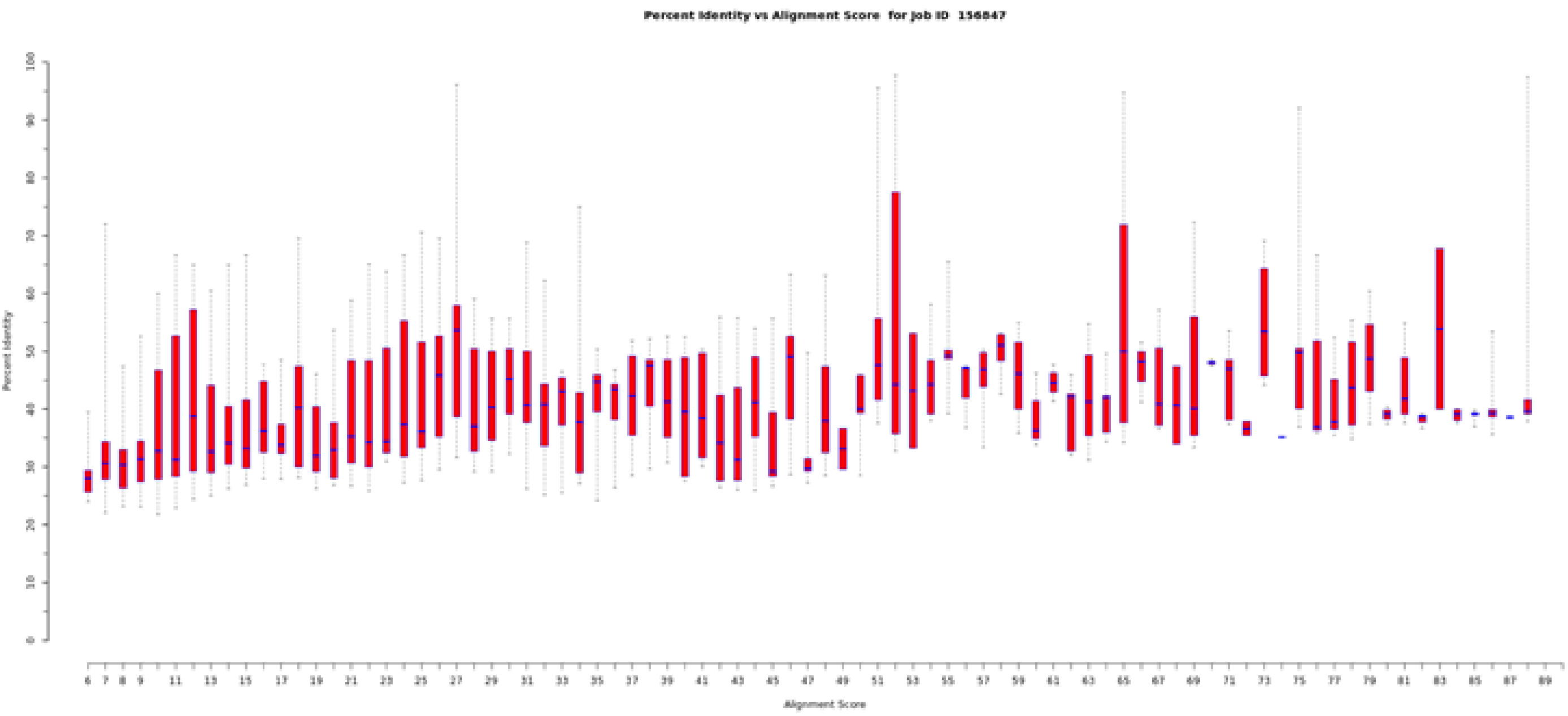

Percent Identity vs Alignment Score for Job ID 156847
Percent Identity
0
10
20
30
40
50
60
70
80
90
100
6
7
8
9
11
13
15
17
19
21
23
25
27
29
31
33
35
37
39
41
43
45
47
49
51
53
55
57
59
61
63
65
67
69
71
73
75
77
79
81
83
85
87
89
Alignment Score

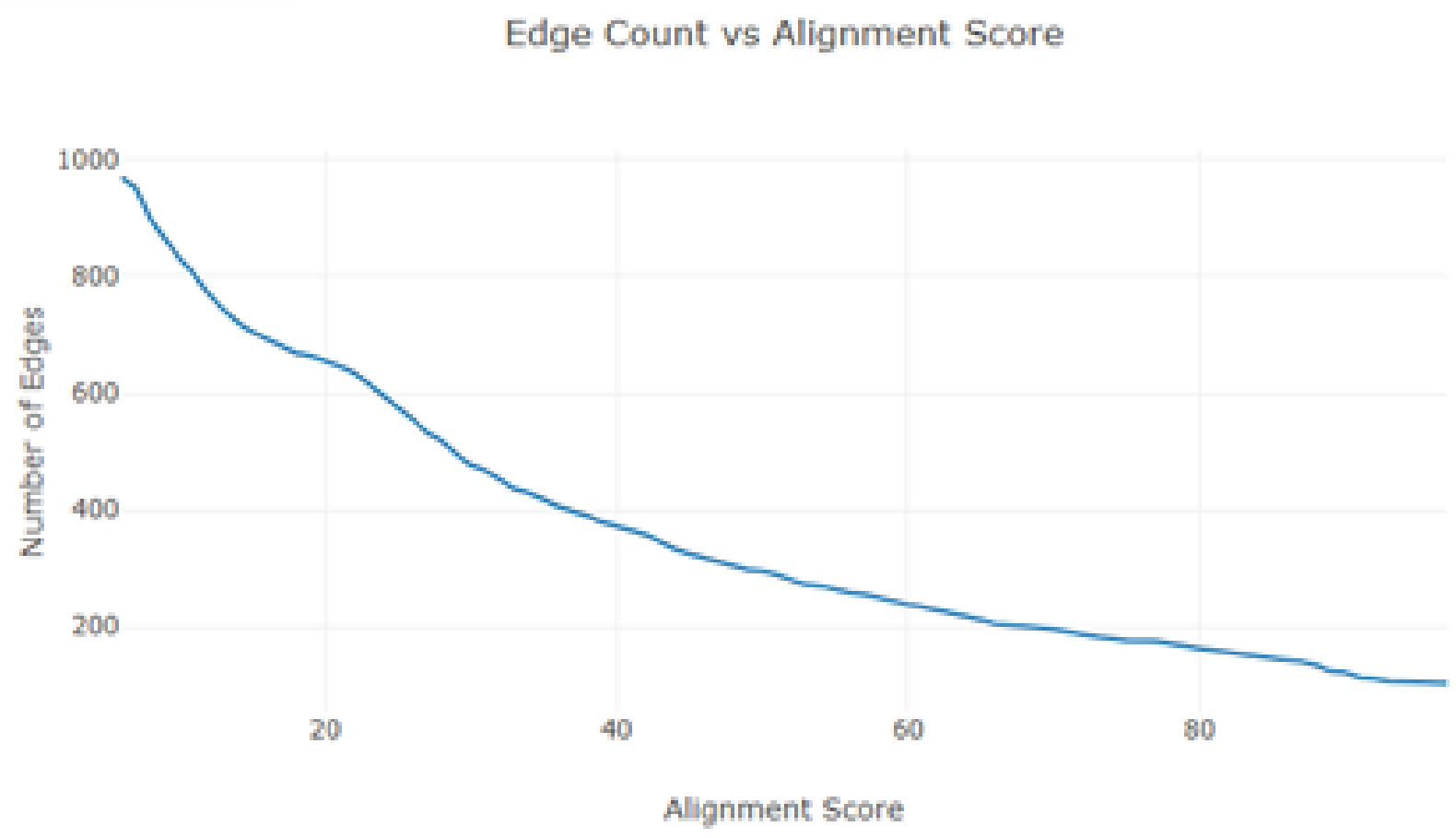

Edge Count vs Alignment Score
1000
800
600
400
200
Number of Edges
20
40
60
80
Alignment Score

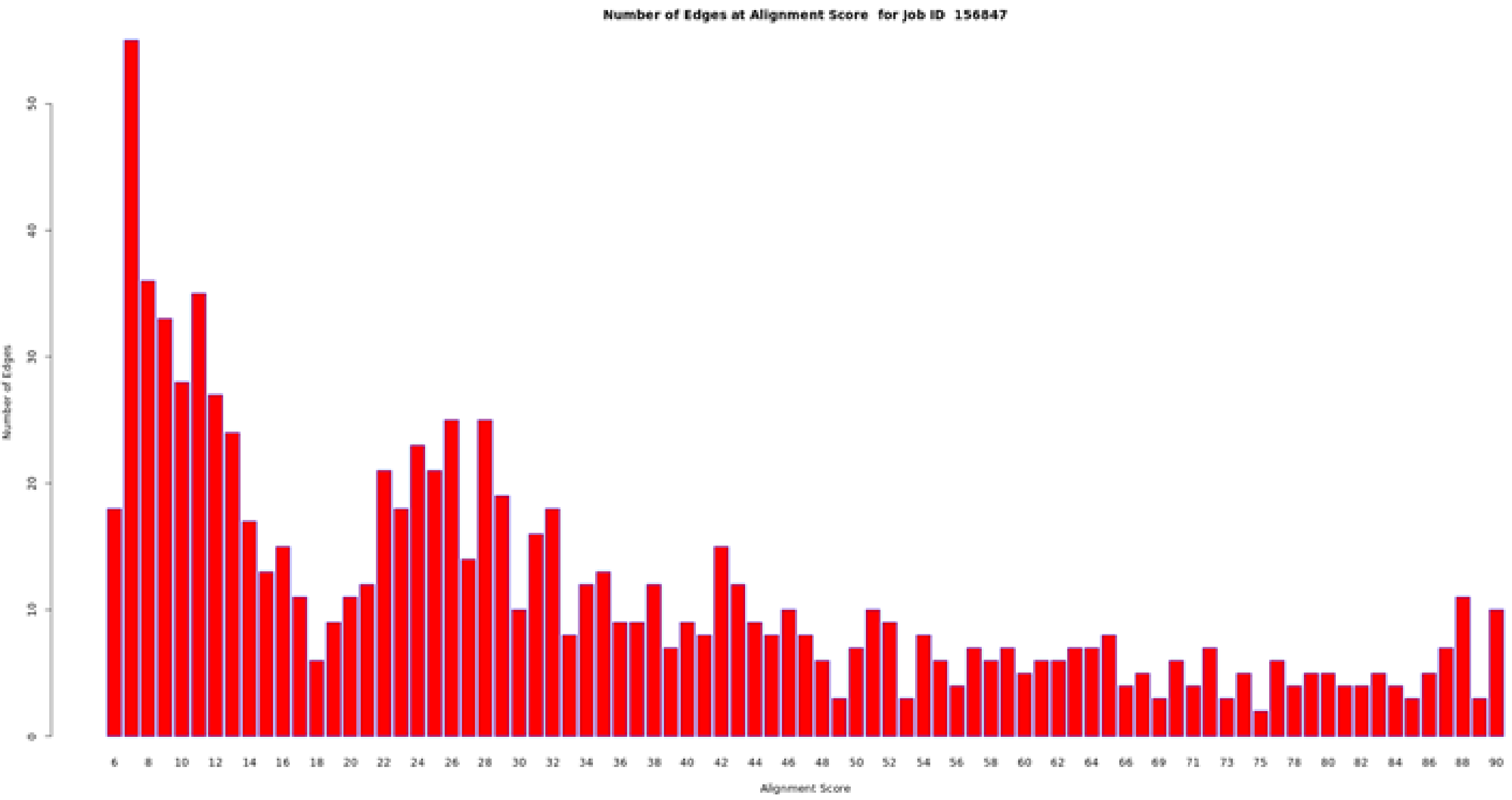
Number of Edges at Alignment Score for Job ID 156847
Number of Edges
Alignment Score

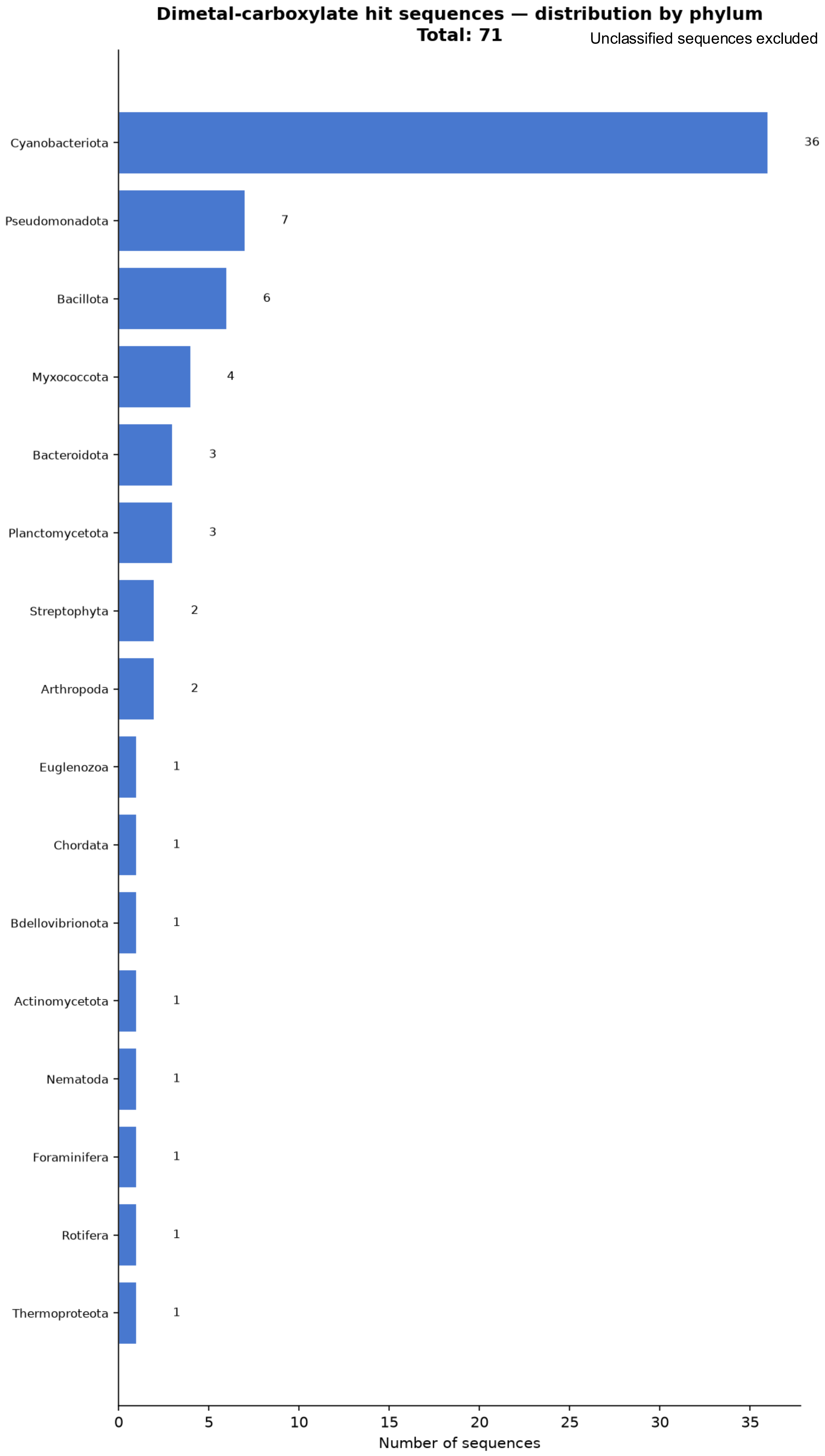

Dimetal-carboxylate hit sequences — distribution by phylum
Total: 71
Unclassified sequences excluded
Cyanobacteriota 36
Pseudomonadota 7
Bacillota 6
Myxococcota 4
Bacteroidota 3
Planctomycetota 3
Streptophyta 2
Arthropoda 2
Euglenozoa 1
Chordata 1
Bdellovibrionota 1
Actinomycetota 1
Nematoda 1
Foraminifera 1
Rotifera 1
Thermoproteota 1
0 5 10 15 20 25 30 35
Number of sequences

SAM-dependent

Job Number	156962
Time Started -- Finished	7/3 01:45 PM -- 7/3 01:49 PM
Database Version	UniProt: 2025-02 / InterPro: 105
Input Option	FASTA (Option C), no FASTA header reading
Job Name	sam-halogenases
E-Value for SSN Edge Calculation	5
Uploaded FASTA File	sam_ssn.fasta
Number of Sequences in Uploaded File	2,605
Exclude Fragments	No
Total Number of Sequences in Dataset	2,604
Total Number of Edges	2,343,179
Number of Unique Sequences	2,604
Convergence Ratio?	0.691

Alignment Score threshold: 60

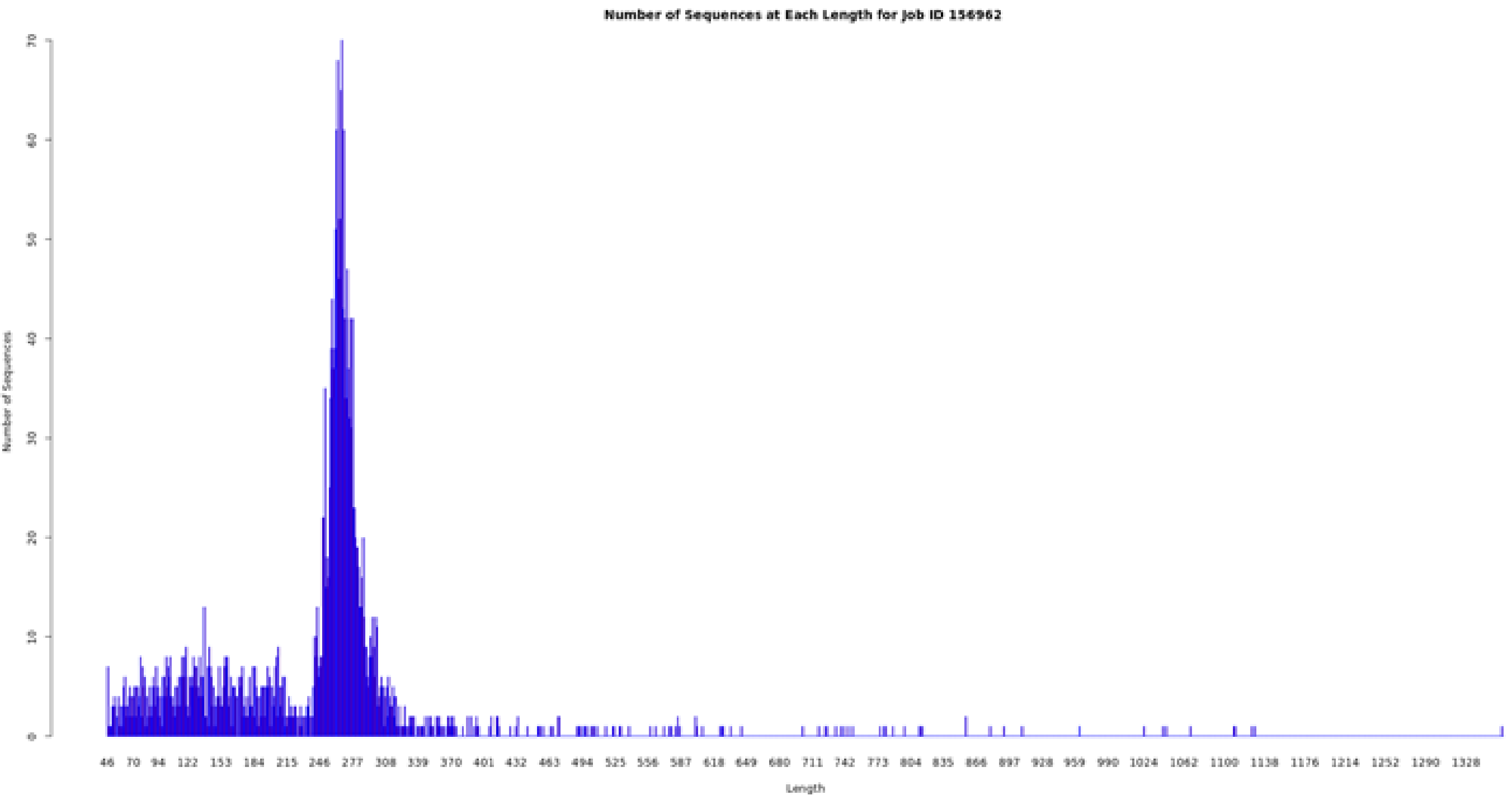

Number of Sequences at Each Length for Job ID 156962
Number of Sequences
Length

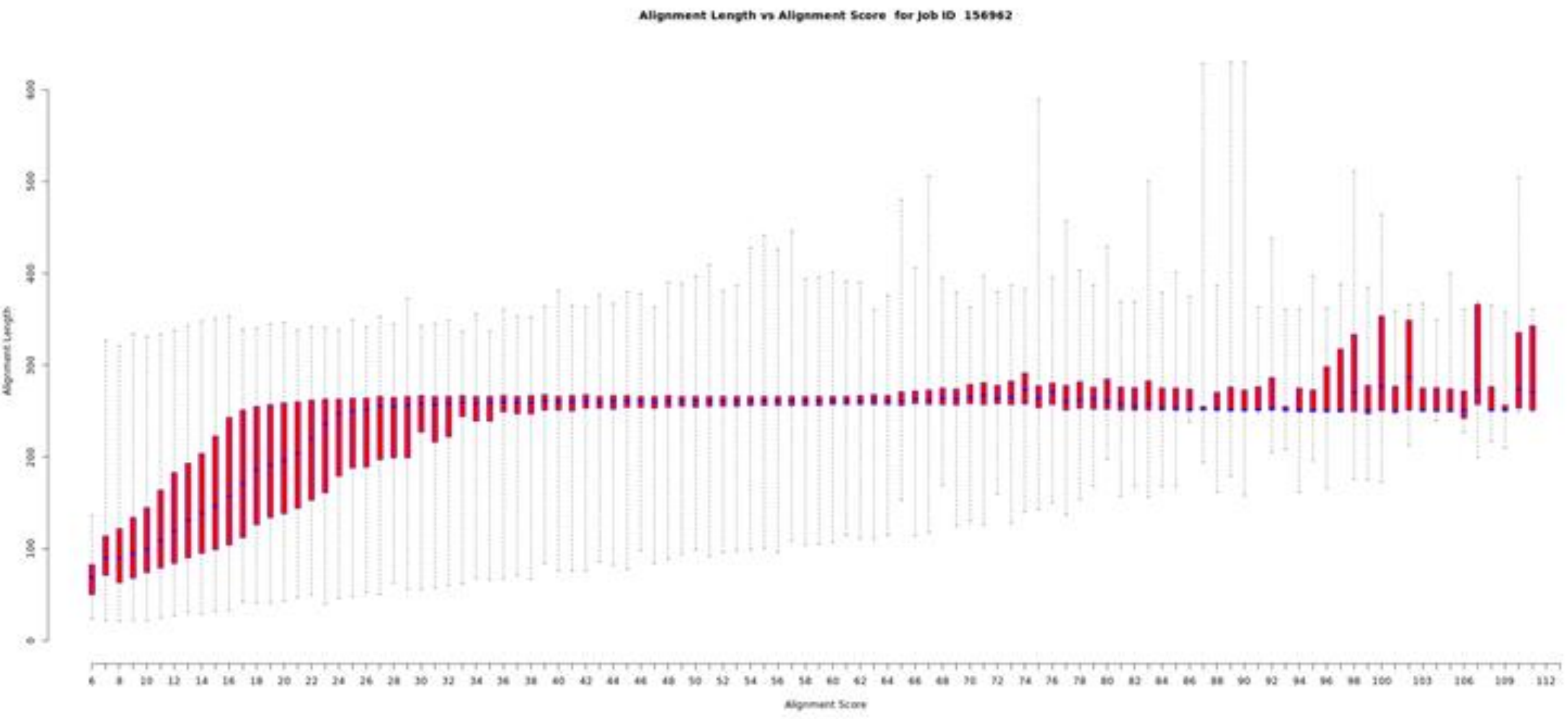

Alignment Length vs Alignment Score for Job ID 156962
Alignment Length
Alignment Score

Percent Identity vs Alignment Score for Job ID 156962

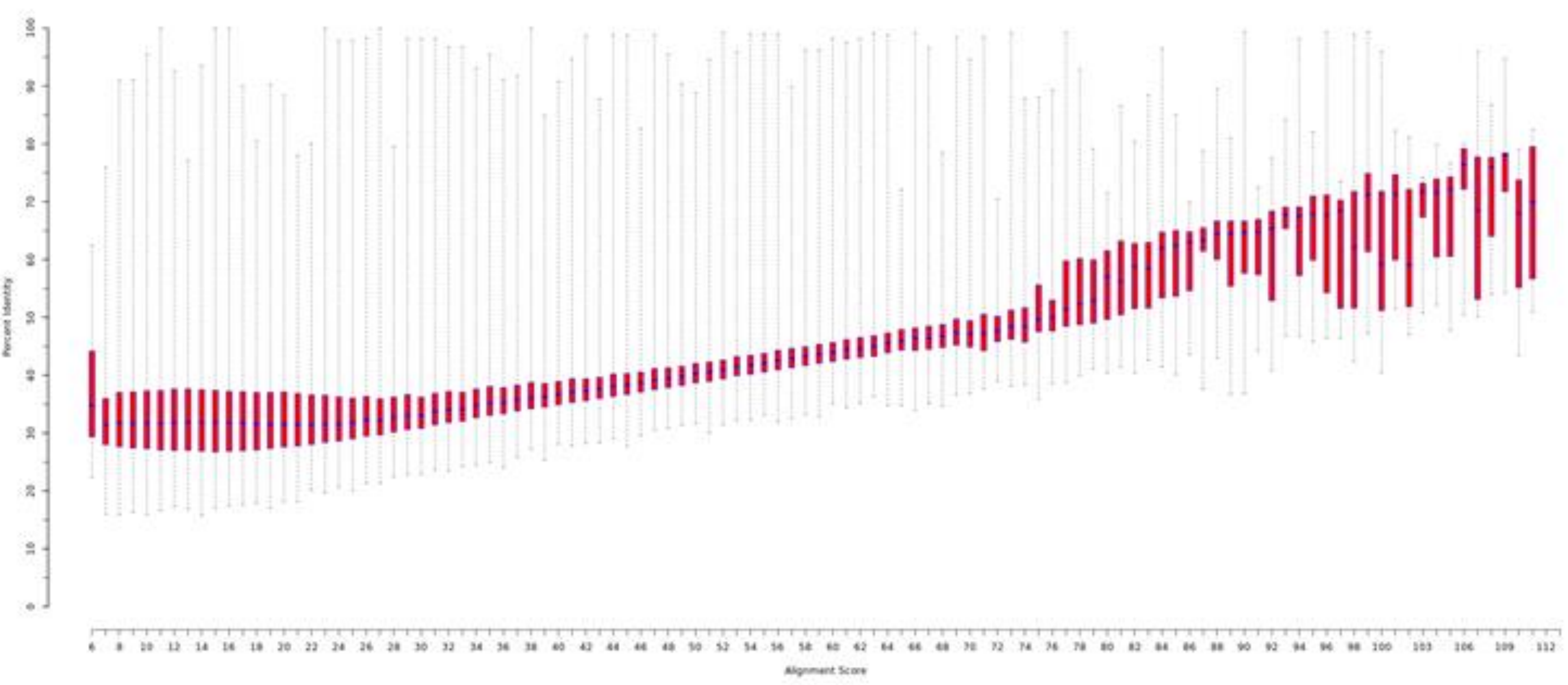

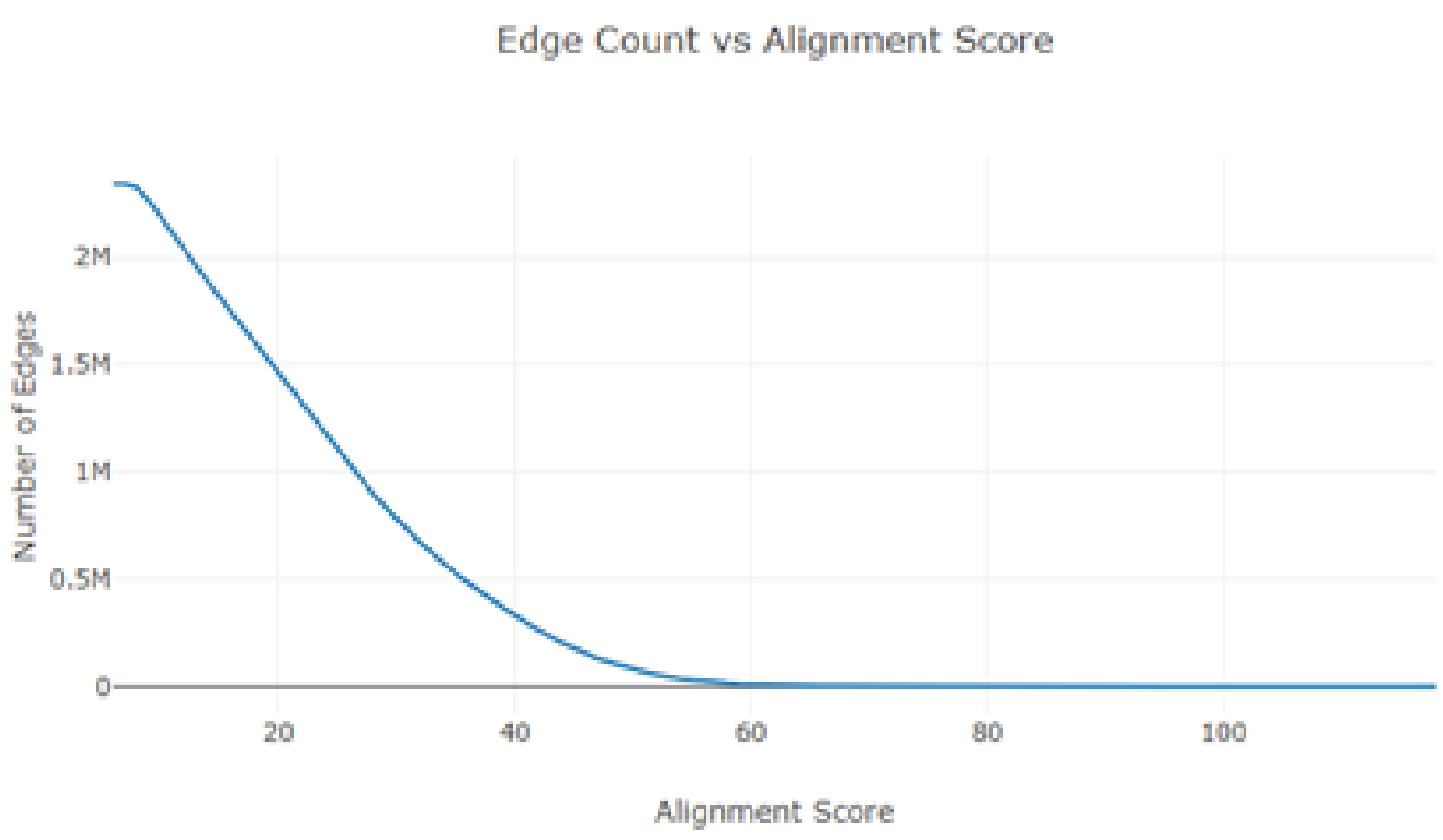
Edge Count vs Alignment Score
Number of Edges
2M
1.5M
1M
0.5M
0
20
40
60
80
100
Alignment Score

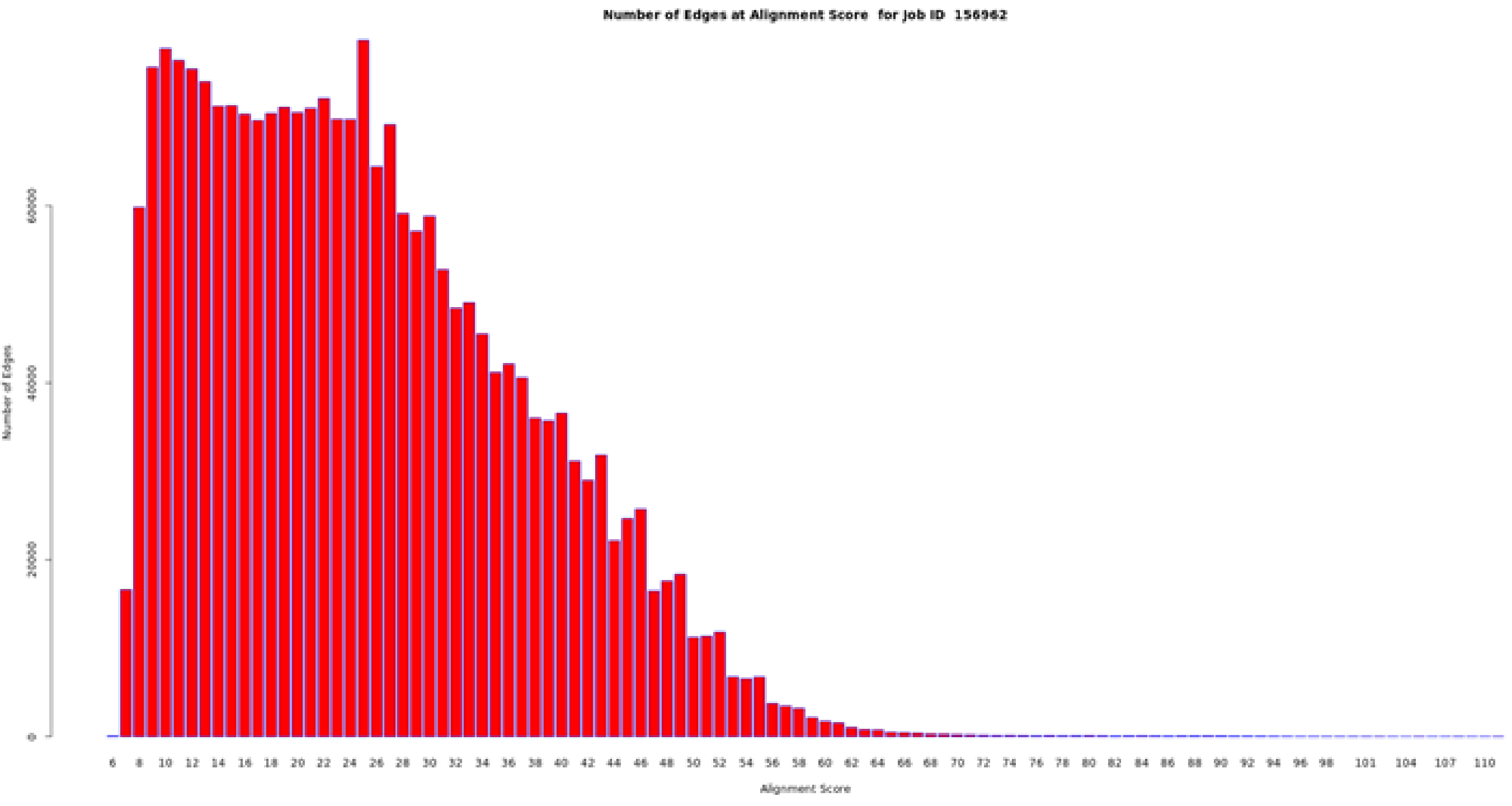

Number of Edges at Alignment Score for Job ID 156962
Number of Edges
0
20000
40000
60000
6
8
10
12
14
16
18
20
22
24
26
28
30
32
34
36
38
40
42
44
46
48
50
52
54
56
58
60
62
64
66
68
70
72
74
76
78
80
82
84
86
88
90
92
94
96
98
101
104
107
110
Alignment Score

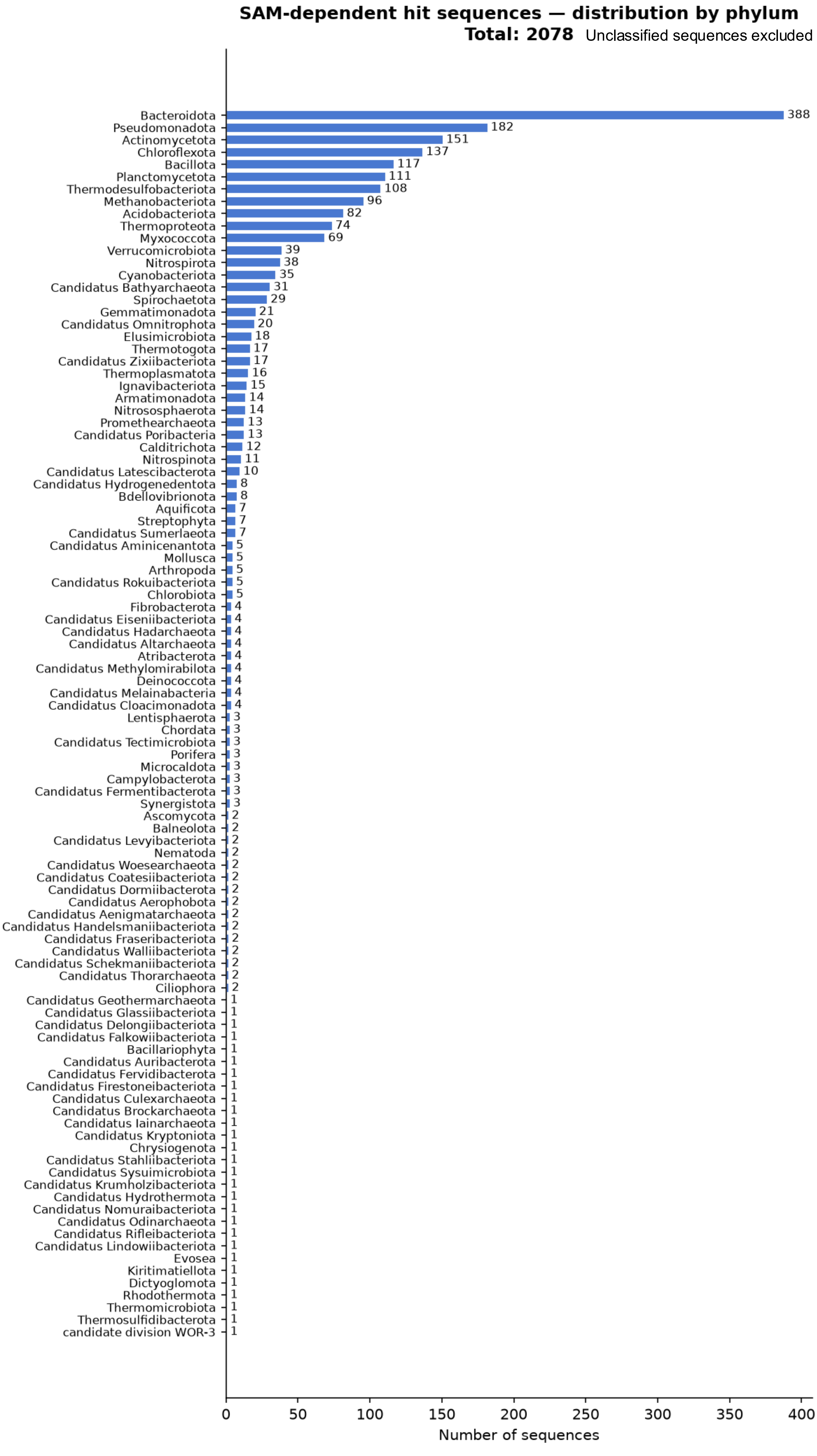

SAM-dependent hit sequences — distribution by phylum
Total: 2078 Unclassified sequences excluded
Bacteroidota 388
Pseudomonadota 182
Actinomycetota 151
Chloroflexota 137
Bacillota 117
Planctomycetota 111
Thermodesulfobacteriota 108
Methanobacteriota 96
Acidobacteriota 82
Thermoproteota 74
Myxococcota 69
Verrucomicrobiota 39
Nitrospirota 38
Cyanobacteriota 35
Candidatus Bathyarchaeota 31
Spirochaetota 29
Gemmatimonadota 21
Candidatus Omnitrophota 20
Elusimicrobiota 18
Thermotogota 17
Candidatus Zixiibacteriota 17
Thermoplasmatota 16
Ignavibacteriota 15
Armatimonadota 14
Nitrososphaerota 14
Promethearchaeota 13
Candidatus Poribacteria 13
Calditrichota 12
Nitrospinota 11
Candidatus Latescibacterota 10
Candidatus Hydrogenedentota 8
Bdellovibrionota 8
Aquificota 7
Streptophyta 7
Candidatus Sumerlaeota 7
Candidatus Aminicenantota 5
Mollusca 5
Arthropoda 5
Candidatus Rokuibacteriota 5
Chlorobiota 5
Fibrobacterota 4
Candidatus Eiseniibacteriota 4
Candidatus Hadarchaeota 4
Candidatus Altarchaeota 4
Atribacterota 4
Candidatus Methylomirabilota 4
Deinococcota 4
Candidatus Melainabacteria 4
Candidatus Cloacimonadota 4
Lentisphaerota 3
Chordata 3
Candidatus Tectimicrobiota 3
Porifera 3
Microcaldota 3
Campylobacterota 3
Candidatus Fermentibacterota 3
Synergistota 3
Ascomycota 2
Balneolota 2
Candidatus Levyibacteriota 2
Nematoda 2
Candidatus Woesearchaeota 2
Candidatus Coatesiibacteriota 2
Candidatus Dormiibacterota 2
Candidatus Aerophobota 2
Candidatus Aenigmatarchaeota 2
Candidatus Handelsmaniibacteriota 2
Candidatus Fraseribacteriota 2
Candidatus Walliibacteriota 2
Candidatus Schekmaniibacteriota 2
Candidatus Thorarchaeota 2
Ciliophora 2
Candidatus Geothermarchaeota 1
Candidatus Glassiibacteriota 1
Candidatus Delongiibacteriota 1
Candidatus Falkowiibacteriota 1
Bacillariophyta 1
Candidatus Auribacterota 1
Candidatus Fervidibacterota 1
Candidatus Firestoneibacteriota 1
Candidatus Culexarchaeota 1
Candidatus Brockarchaeota 1
Candidatus Iainarchaeota 1
Candidatus Kryptoniota 1
Chrysiogenota 1
Candidatus Stahliibacteriota 1
Candidatus Sysuimicrobiota 1
Candidatus Krumholzibacteriota 1
Candidatus Hydrothermota 1
Candidatus Nomuraibacteriota 1
Candidatus Odinarchaeota 1
Candidatus Rifleibacteriota 1
Candidatus Lindowiibacteriota 1
Evosea 1
Kiritimatiellota 1
Dictyoglomota 1
Rhodothermota 1
Thermomicrobiota 1
Thermosulfidibacterota 1
candidate division WOR-3 1
0
50
100
150
200
250
300
350
400
Number of sequences

Flavin-dependent (unconventional)

Job Number	157052
Time Started -- Finished	7/6 01:20 AM -- 7/6 01:23 AM
Database Version	UniProt: 2025-02 / InterPro: 105
Input Option	FASTA (Option C), no FASTA header reading
Job Name	FDH-unconventional
E-Value for SSN Edge Calculation	5
Uploaded FASTA File	fdh_unconventional.fasta
Number of Sequences in Uploaded File	193
Exclude Fragments	No
Total Number of Sequences in Dataset	193
Total Number of Edges	3,530
Number of Unique Sequences	193
Convergence Ratio?	0.191

Alignment Score threshold: 45

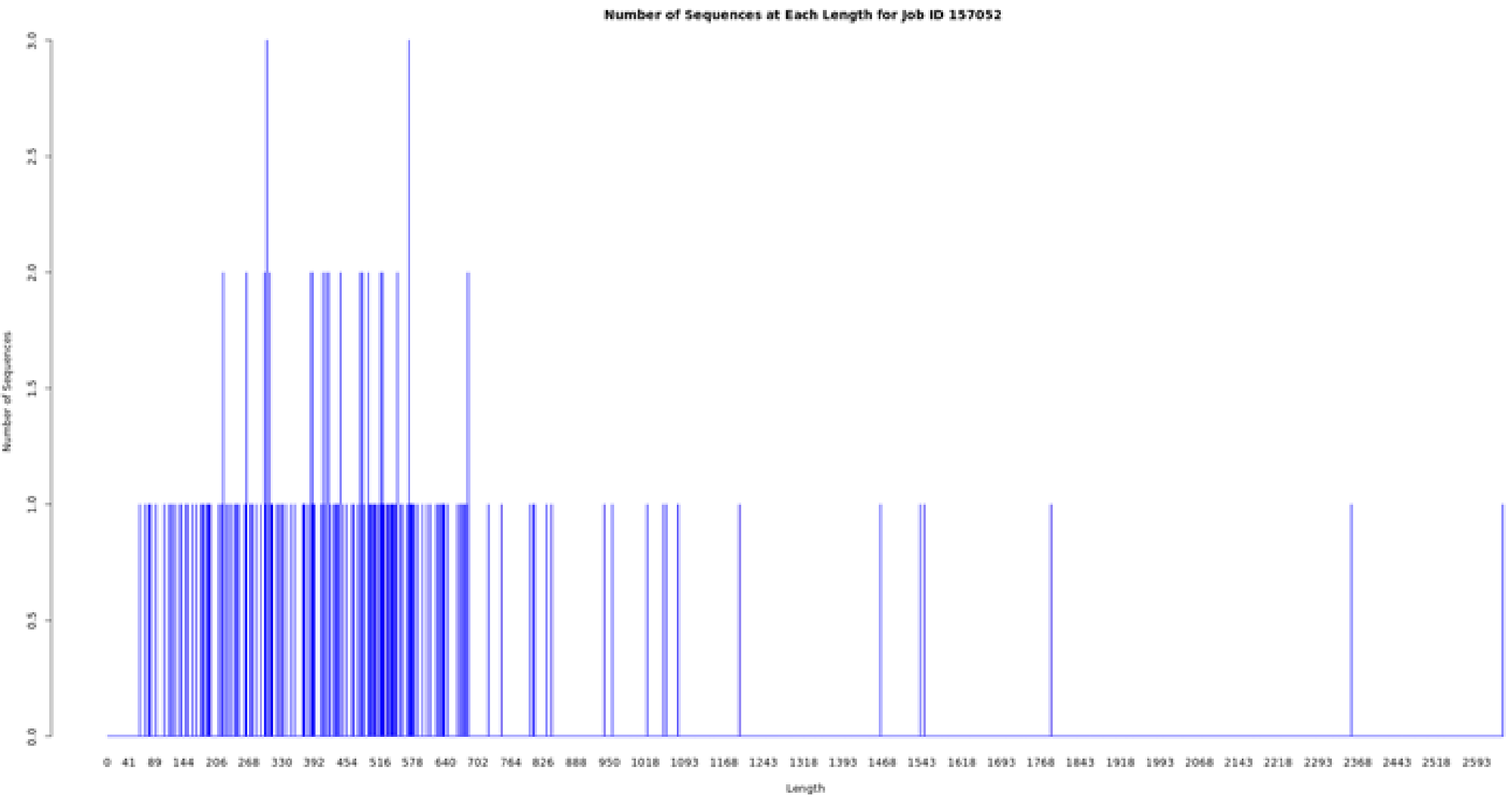

Number of Sequences at Each Length for Job ID 157052
Number of Sequences
Length

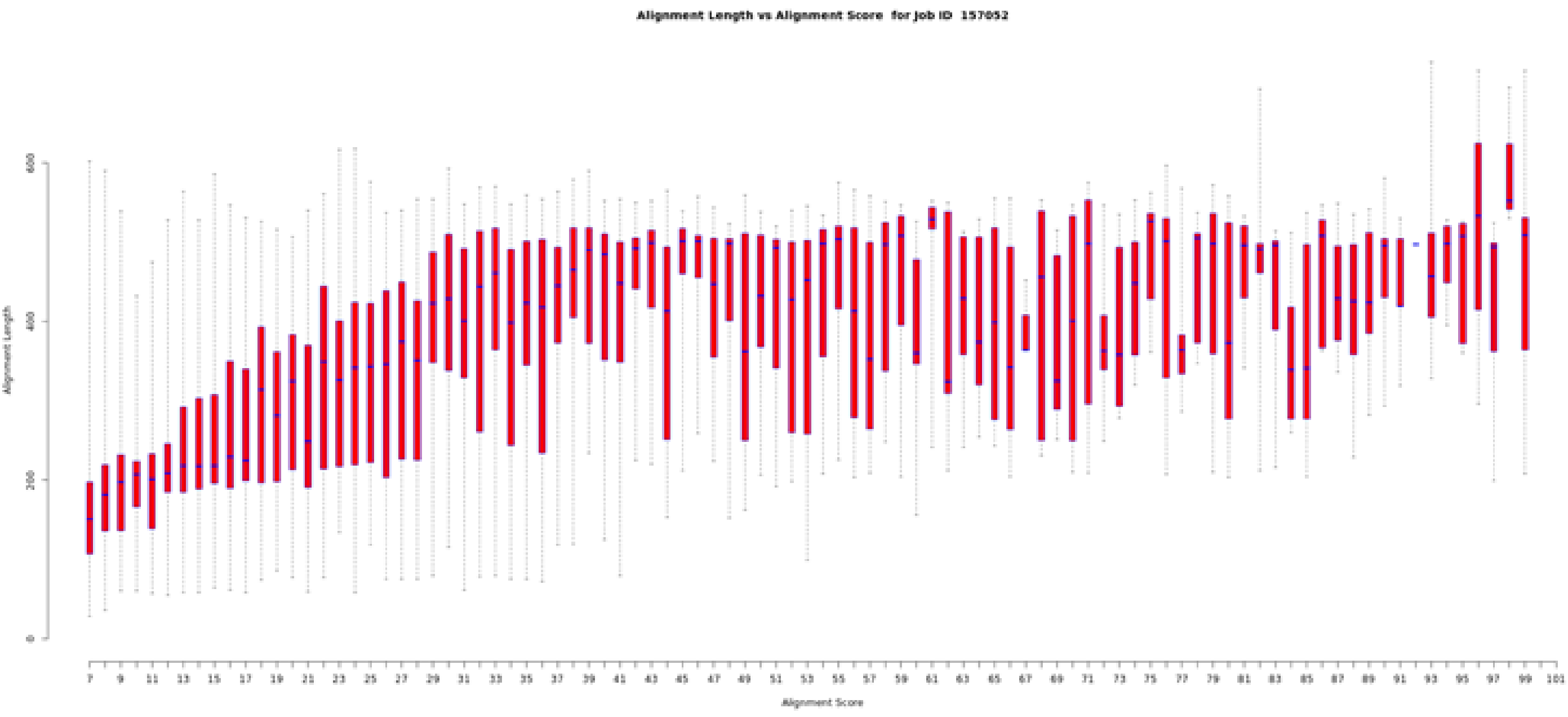

Alignment Length vs Alignment Score for Job ID 157052
Alignment Length
Alignment Score

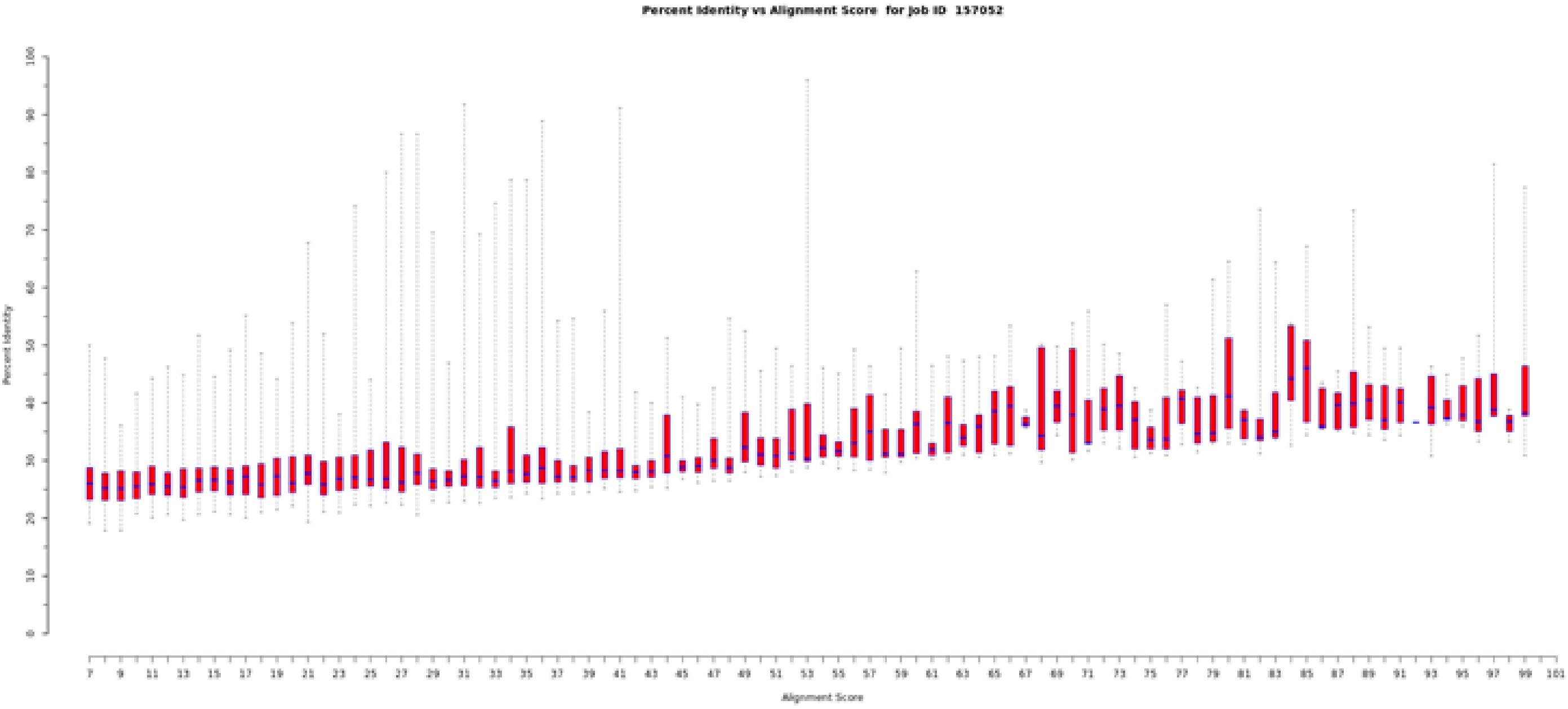
Percent Identity vs Alignment Score for Job ID 157052
Percent Identity
Alignment Score

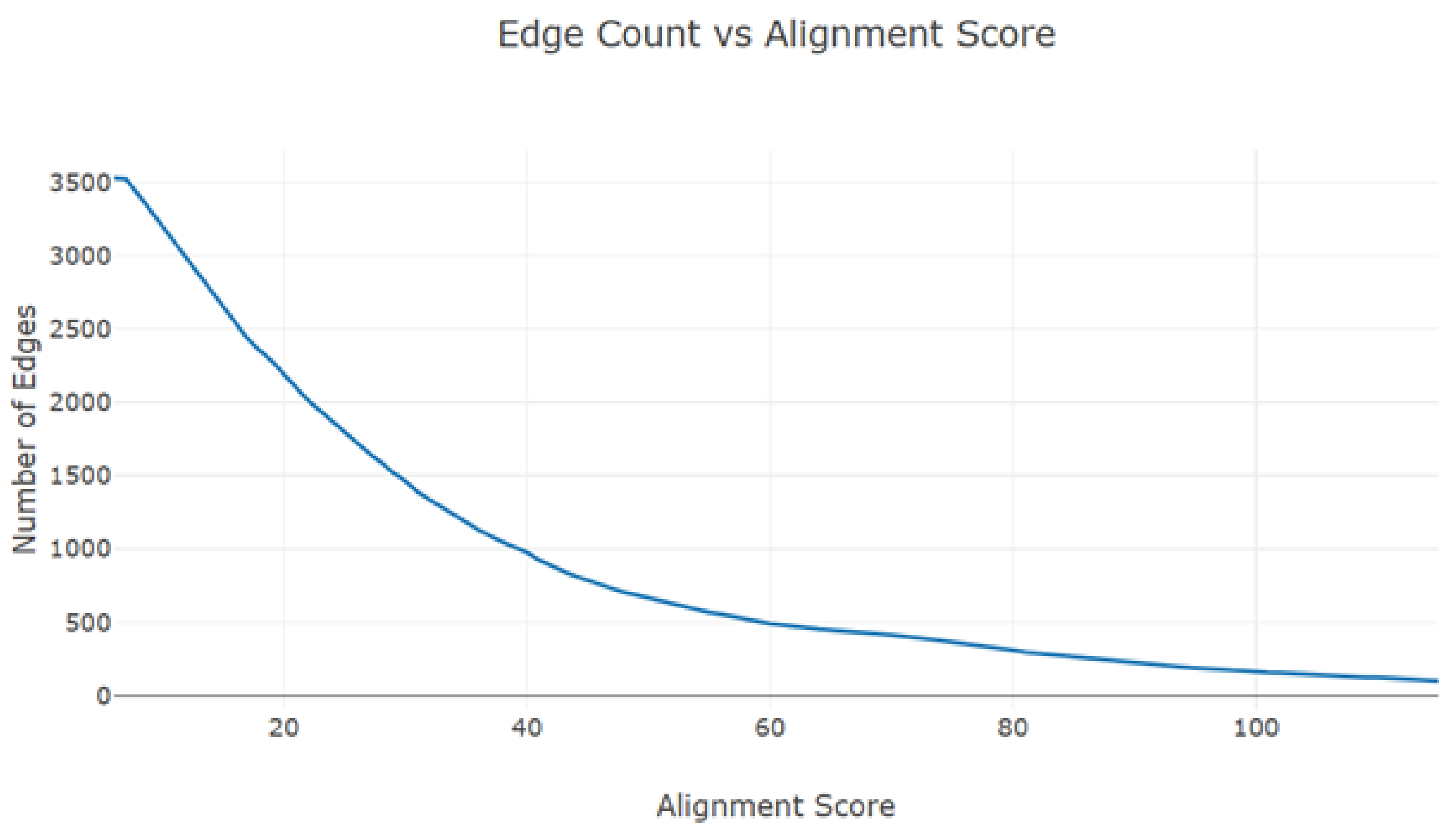

Edge Count vs Alignment Score
3500
3000
2500
2000
1500
1000
500
0
Number of Edges
20
40
60
80
100
Alignment Score

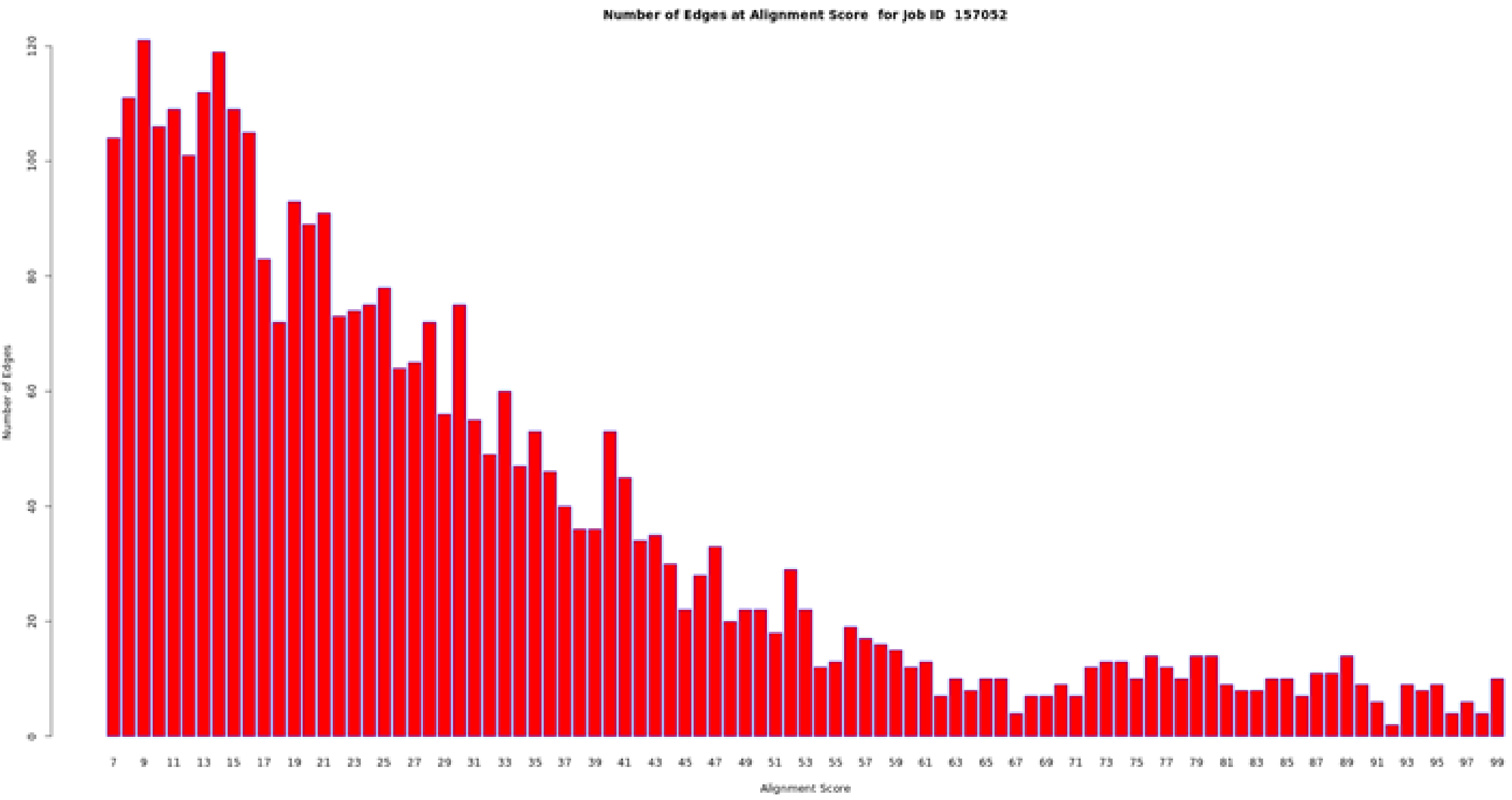
Number of Edges at Alignment Score for Job ID 157052
Number of Edges
Alignment Score
0
20
40
60
80
100
120
7 9 11 13 15 17 19 21 23 25 27 29 31 33 35 37 39 41 43 45 47 49 51 53 55 57 59 61 63 65 67 69 71 73 75 77 79 81 83 85 87 89 91 93 95 97 99

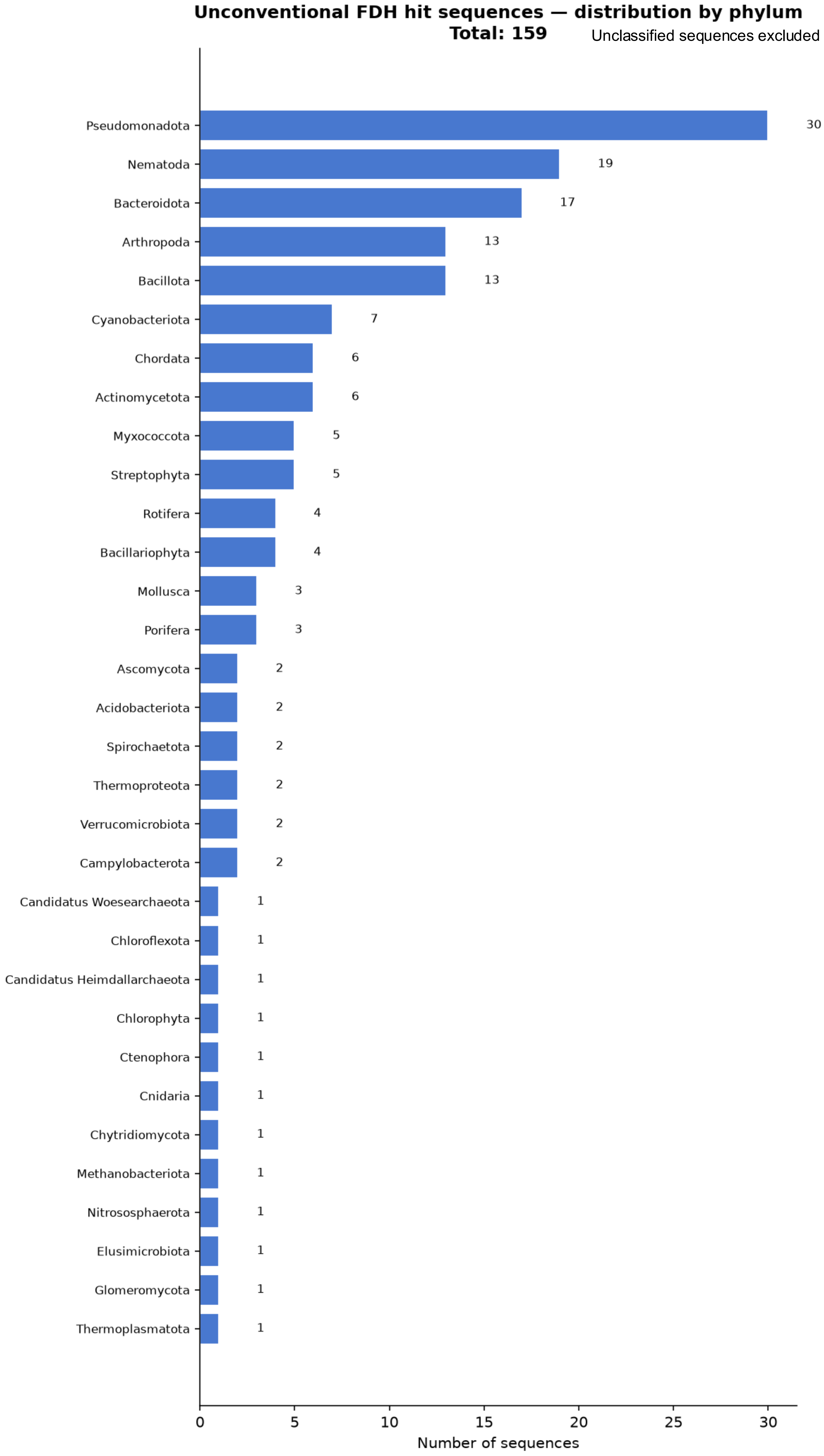
Unconventional FDH hit sequences — distribution by phylum
Total: 159
Unclassified sequences excluded
Pseudomonadota 30
Nematoda 19
Bacteroidota 17
Arthropoda 13
Bacillota 13
Cyanobacteriota 7
Chordata 6
Actinomycetota 6
Myxococcota 5
Streptophyta 5
Rotifera 4
Bacillariophyta 4
Mollusca 3
Porifera 3
Ascomycota 2
Acidobacteriota 2
Spirochaetota 2
Thermoproteota 2
Verrucomicrobiota 2
Campylobacterota 2
Candidatus Woesearchaeota 1
Chloroflexota 1
Candidatus Heimdallarchaeota 1
Chlorophyta 1
Ctenophora 1
Cnidaria 1
Chytridiomycota 1
Methanobacteriota 1
Nitrososphaerota 1
Elusimicrobiota 1
Glomeromycota 1
Thermoplasmatota 1
0
5
10
15
20
25
30
Number of sequences

Flavin-dependent (conventional)

Job Number	157098
Time Started -- Finished	7/7 03:29 AM -- 7/7 03:33 AM
Database Version	UniProt: 2025-02 / InterPro: 105
Input Option	FASTA (Option C), no FASTA header reading
Job Name	FDH_conventional
E-Value for SSN Edge Calculation	5
Uploaded FASTA File	fdh_conventional_ssn.fasta
Number of Sequences in Uploaded File	1,960
Exclude Fragments	No
Total Number of Sequences in Dataset	1,960
Total Number of Edges	751,781
Number of Unique Sequences	1,960
Convergence Ratio?	0.392

Alignment Score threshold: 100

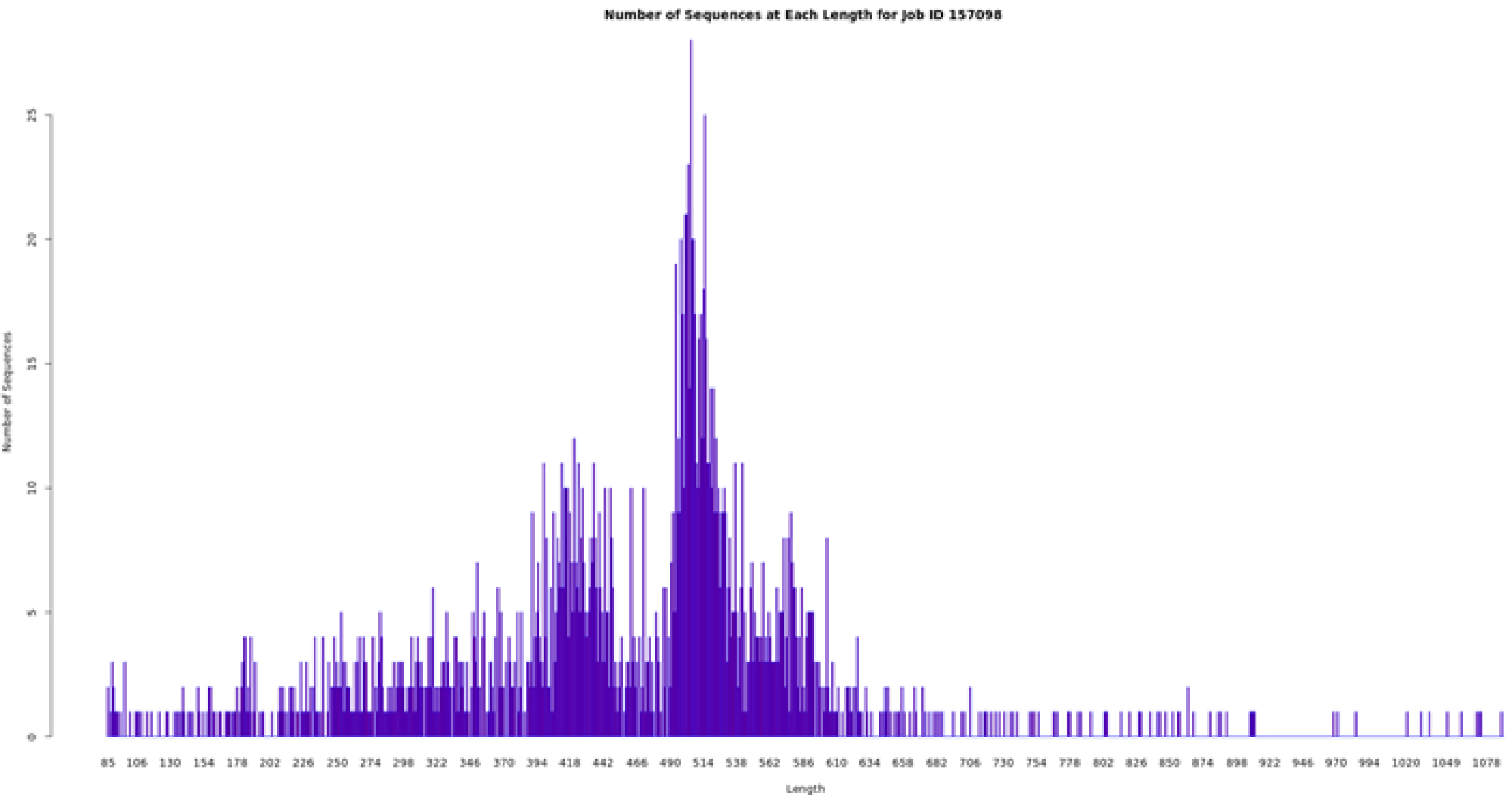
Number of Sequences at Each Length for Job ID 157098
Number of Sequences
Length

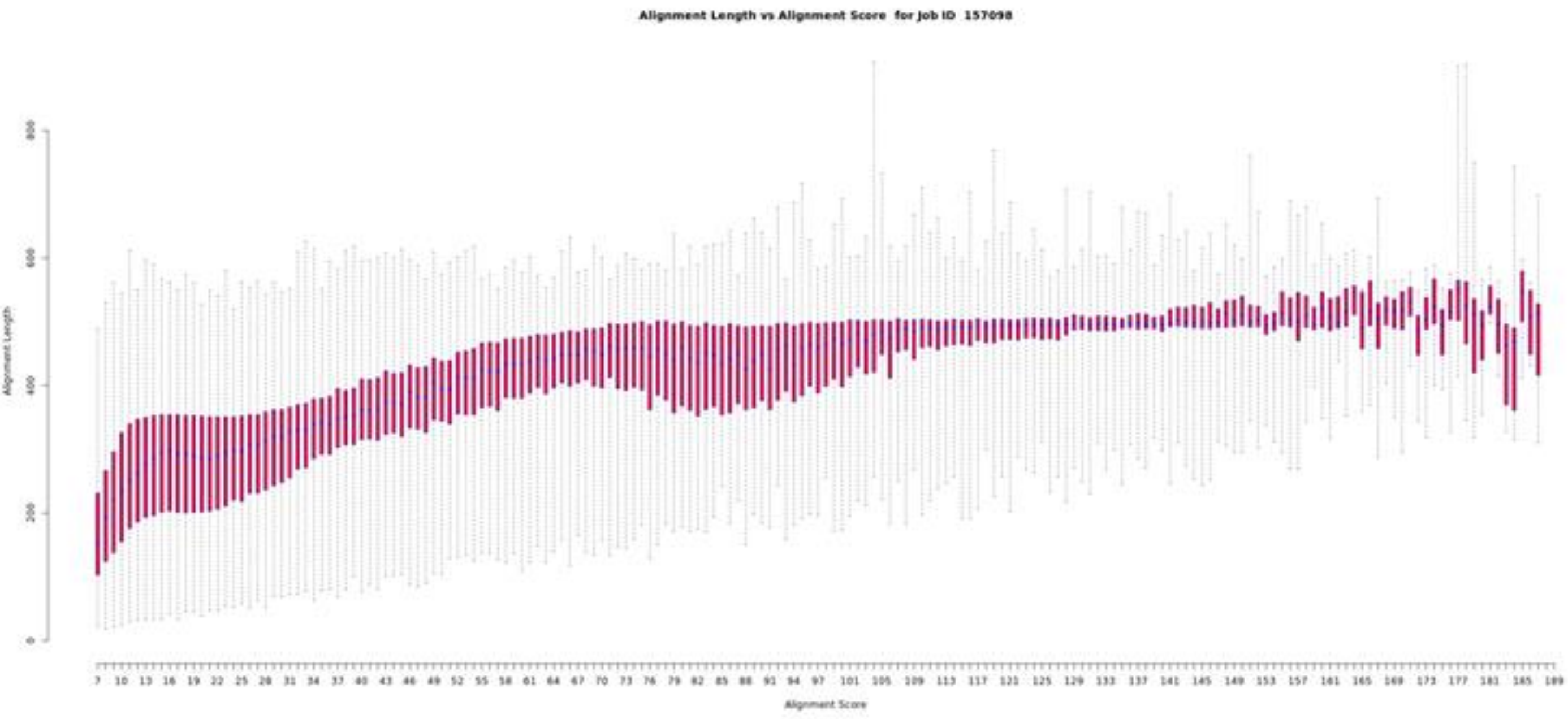

Alignment Length vs Alignment Score for Job ID 157098
Alignment Length
0
200
400
600
800
Alignment Score

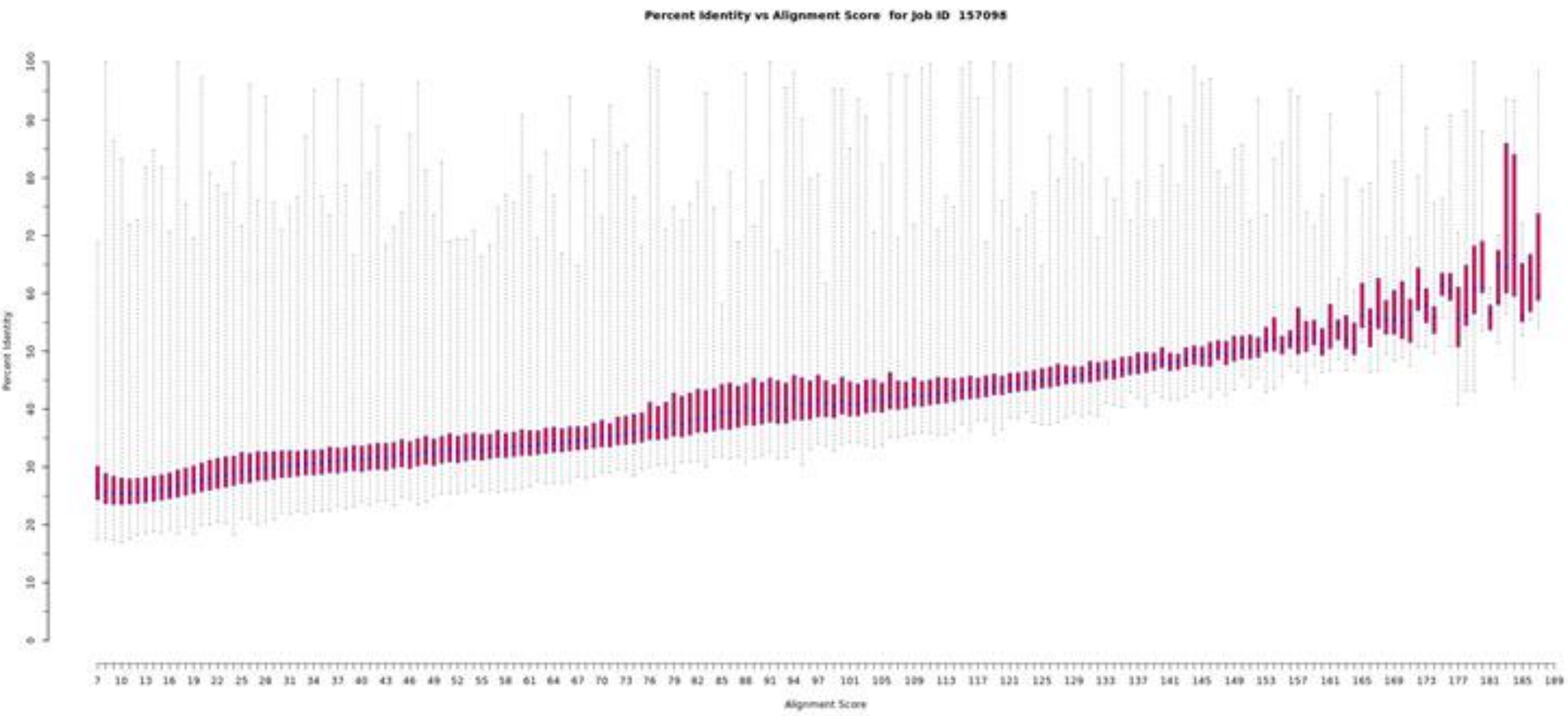
Percent Identity vs Alignment Score for Job ID 157098
Percent Identity
0
10
20
30
40
50
60
70
80
90
100
Alignment Score
7 10 13 16 19 22 25 28 31 34 37 40 43 46 49 52 55 58 61 64 67 70 73 76 79 82 85 88 91 94 97 101 105 109 113 117 121 125 129 133 137 141 145 149 153 157 161 165 169 173 177 181 185 189

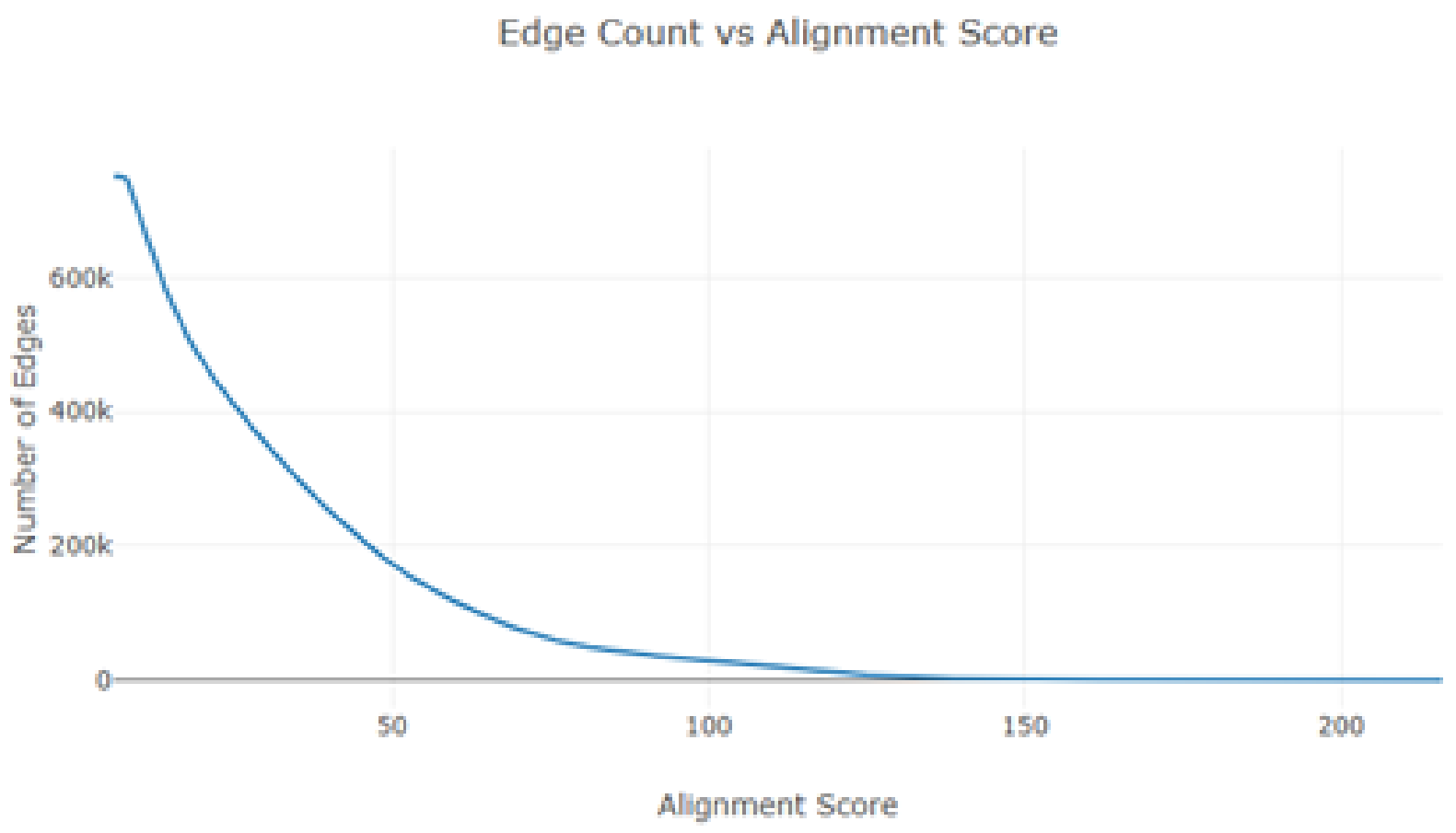
Edge Count vs Alignment Score
Number of Edges
600k
400k
200k
0
50
100
150
200
Alignment Score

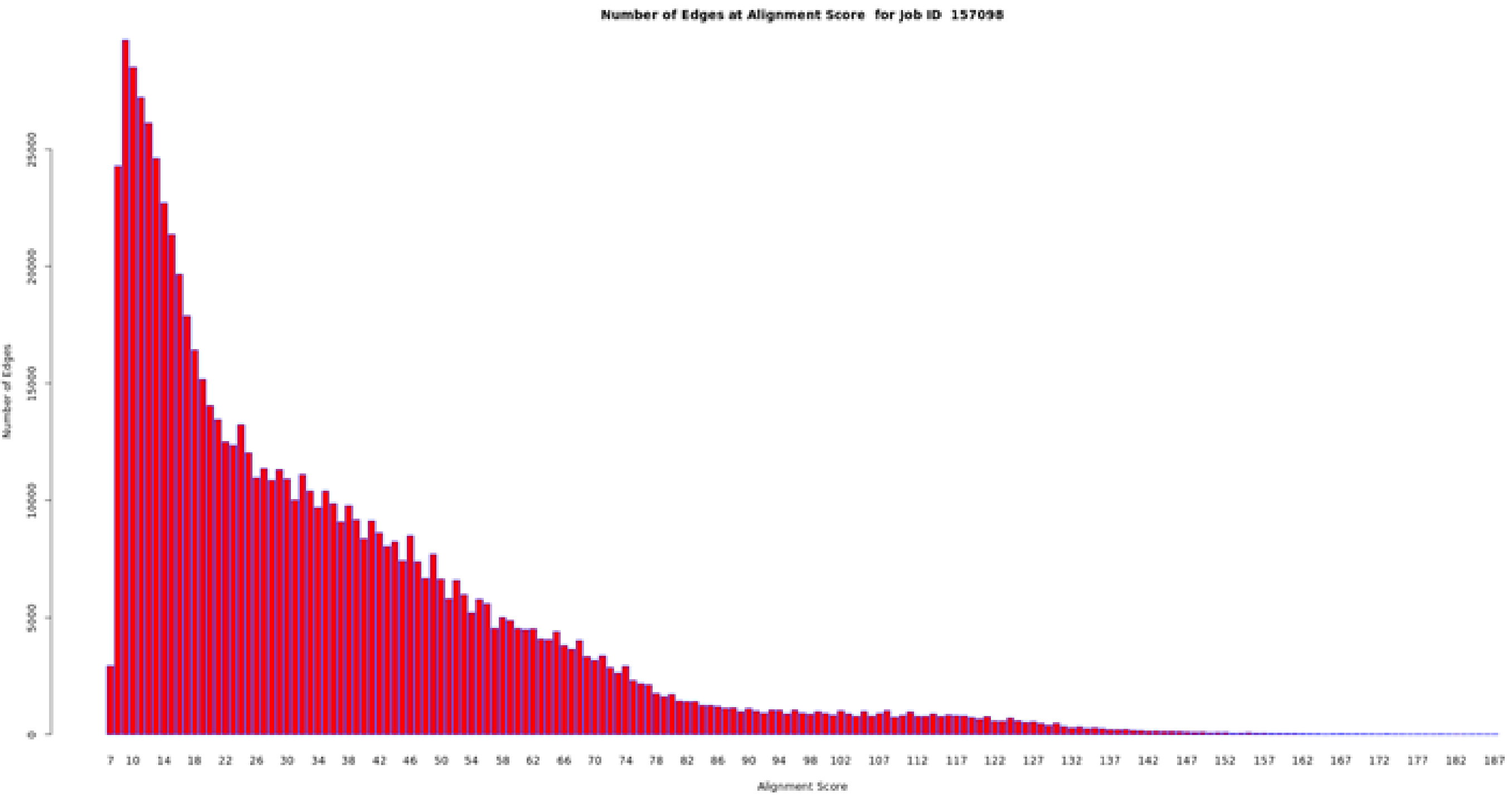

Number of Edges at Alignment Score for Job ID 157098
Number of Edges
0
5000
10000
15000
20000
25000
7 10 14 18 22 26 30 34 38 42 46 50 54 58 62 66 70 74 78 82 86 90 94 98 102 107 112 117 122 127 132 137 142 147 152 157 162 167 172 177 182 187
Alignment Score

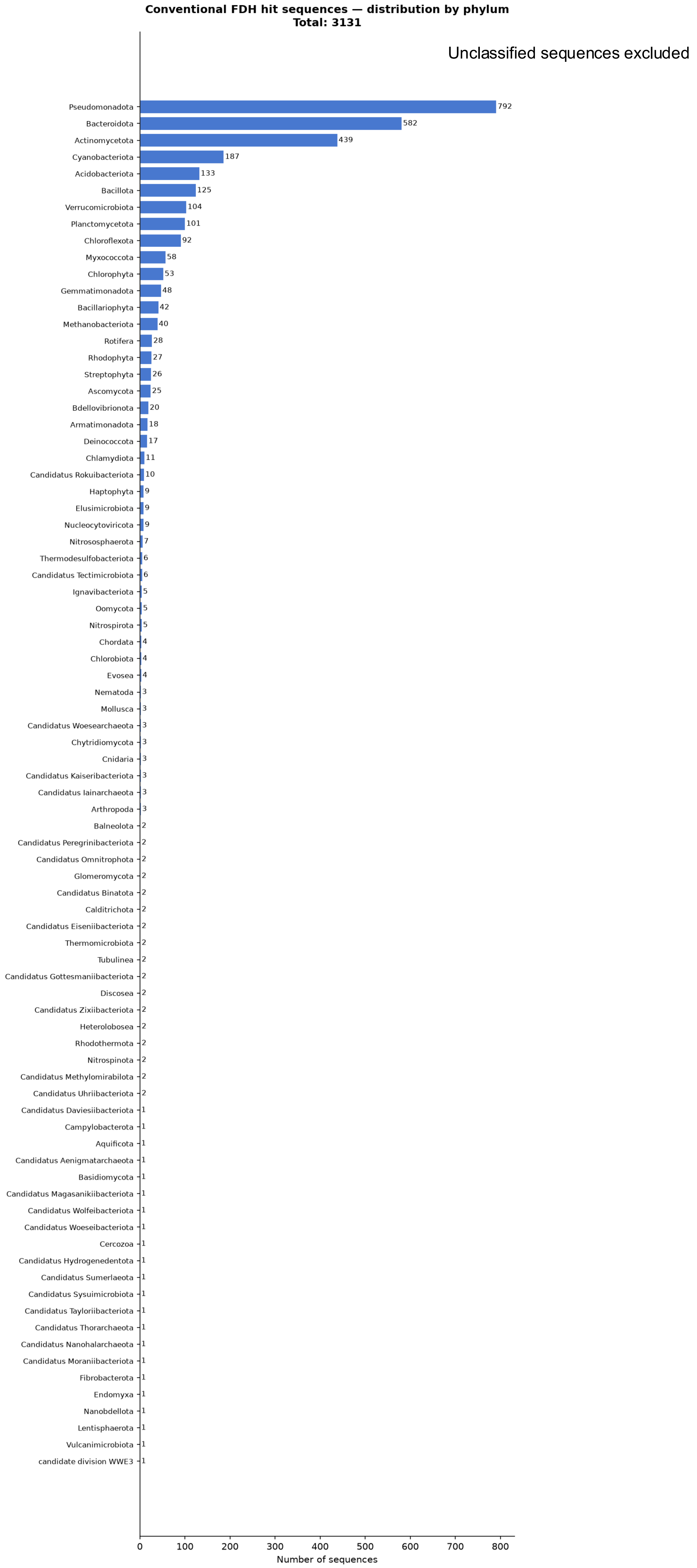
Conventional FDH hit sequences — distribution by phylum
Total: 3131
Unclassified sequences excluded
Pseudomonadota 792
Bacteroidota 582
Actinomycetota 439
Cyanobacteriota 187
Acidobacteriota 133
Bacillota 125
Verrucomicrobiota 104
Planctomycetota 101
Chloroflexota 92
Myxococcota 58
Chlorophyta 53
Gemmatimonadota 48
Bacillariophyta 42
Methanobacteriota 40
Rotifera 28
Rhodophyta 27
Streptophyta 26
Ascomycota 25
Bdellovibrionota 20
Armatimonadota 18
Deinococcota 17
Chlamydiota 11
Candidatus Rokuibacteriota 10
Haptophyta 9
Elusimicrobiota 9
Nucleocytoviricota 9
Nitrososphaerota 7
Thermodesulfobacteriota 6
Candidatus Tectimicrobiota 6
Ignavibacteriota 5
Oomycota 5
Nitrospirota 5
Chordata 4
Chlorobiota 4
Evosea 4
Nematoda 3
Mollusca 3
Candidatus Woesearchaeota 3
Chytridiomycota 3
Cnidaria 3
Candidatus Kaiseribacteriota 3
Candidatus Iainarchaeota 3
Arthropoda 3
Balneolota 2
Candidatus Peregrinibacteriota 2
Candidatus Omnitrophota 2
Glomeromycota 2
Candidatus Binatota 2
Calditrichota 2
Candidatus Eiseniibacteriota 2
Thermomicrobiota 2
Tubulinea 2
Candidatus Gottesmaniibacteriota 2
Discosea 2
Candidatus Zixiibacteriota 2
Heterolobosea 2
Rhodothermota 2
Nitrospinota 2
Candidatus Methylomirabilota 2
Candidatus Uhriibacteriota 2
Candidatus Daviesiibacteriota 1
Campylobacterota 1
Aquificota 1
Candidatus Aenigmatarchaeota 1
Basidiomycota 1
Candidatus Magasanikiibacteriota 1
Candidatus Wolfeibacteriota 1
Candidatus Woeseibacteriota 1
Cercozoa 1
Candidatus Hydrogenedentota 1
Candidatus Sumerlaeota 1
Candidatus Sysuimicrobiota 1
Candidatus Tayloriibacteriota 1
Candidatus Thorarchaeota 1
Candidatus Nanohalarchaeota 1
Candidatus Moraniibacteriota 1
Fibrobacterota 1
Endomyxa 1
Nanobdellota 1
Lentisphaerota 1
Vulcanimicrobiota 1
candidate division WWE3 1
0
100
200
300
400
500
600
700
800
Number of sequences

Non-heme iron-dependent

Job Number	157360
Time Started -- Finished	7/11 12:05 AM -- 7/11 12:08 AM
Database Version	UniProt: 2025-03 / InterPro: 106
Input Option	FASTA (Option C), no FASTA header reading
Job Name	NHFe
E-Value for SSN Edge Calculation	5
Uploaded FASTA File	nhfe_final_ssn.fasta
Number of Sequences in Uploaded File	1,134
Exclude Fragments	No
Total Number of Sequences in Dataset	1,134
Total Number of Edges	177,036
Number of Unique Sequences	1,134
Convergence Ratio?	0.276

Alignment Score threshold: 45

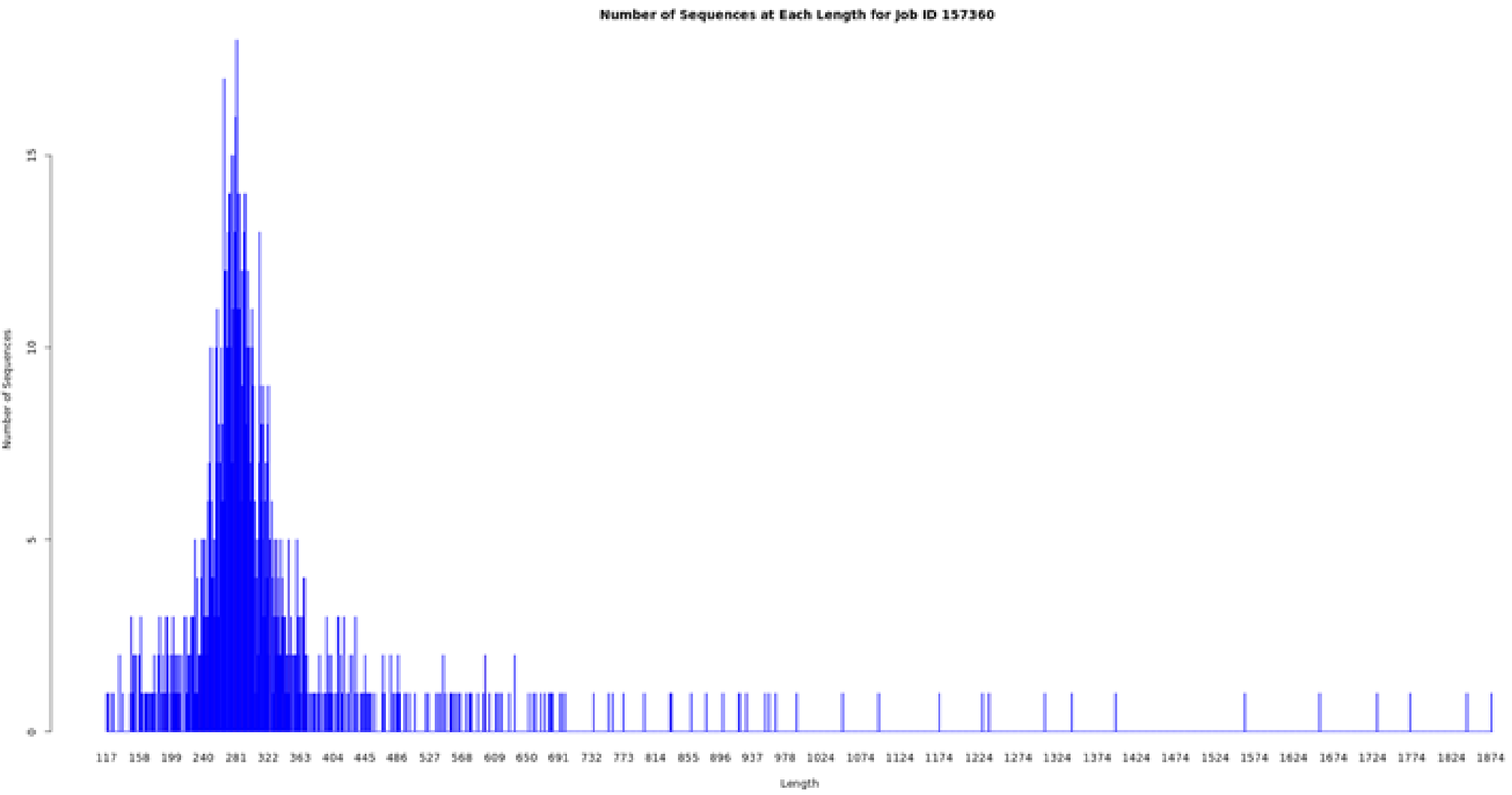

Number of Sequences at Each Length for Job ID 157360
Number of Sequences
0
5
10
15
117
158
199
240
281
322
363
404
445
486
527
568
609
650
691
732
773
814
855
896
937
978
1024
1074
1124
1174
1224
1274
1324
1374
1424
1474
1524
1574
1624
1674
1724
1774
1824
1874
Length

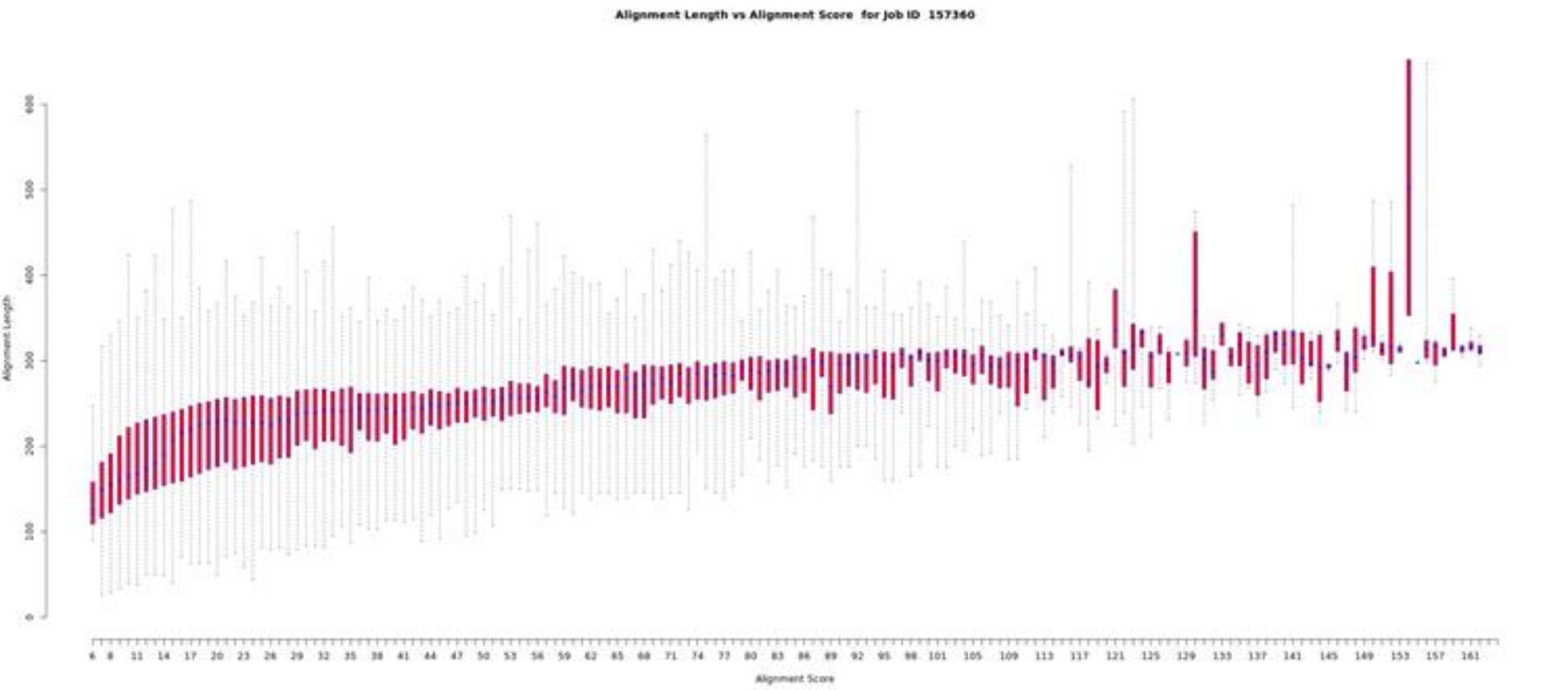

Alignment Length vs Alignment Score for Job ID 157360
Alignment Length
0
100
200
300
400
500
600
Alignment Score
6 8 11 14 17 20 23 26 29 32 35 38 41 44 47 50 53 56 59 62 65 68 71 74 77 80 83 86 89 92 95 98 101 105 109 113 117 121 125 129 133 137 141 145 149 153 157 161

Percent Identity vs Alignment Score for Job ID 157360

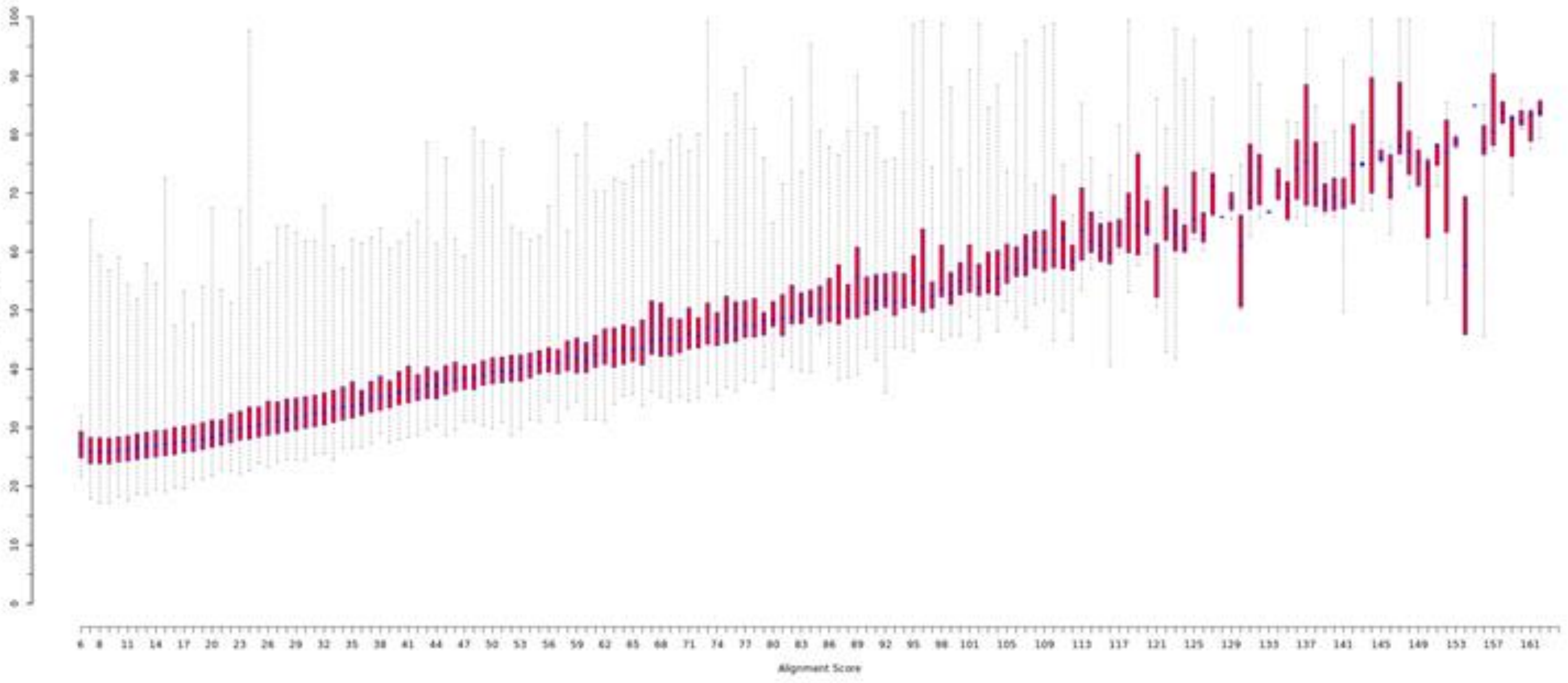

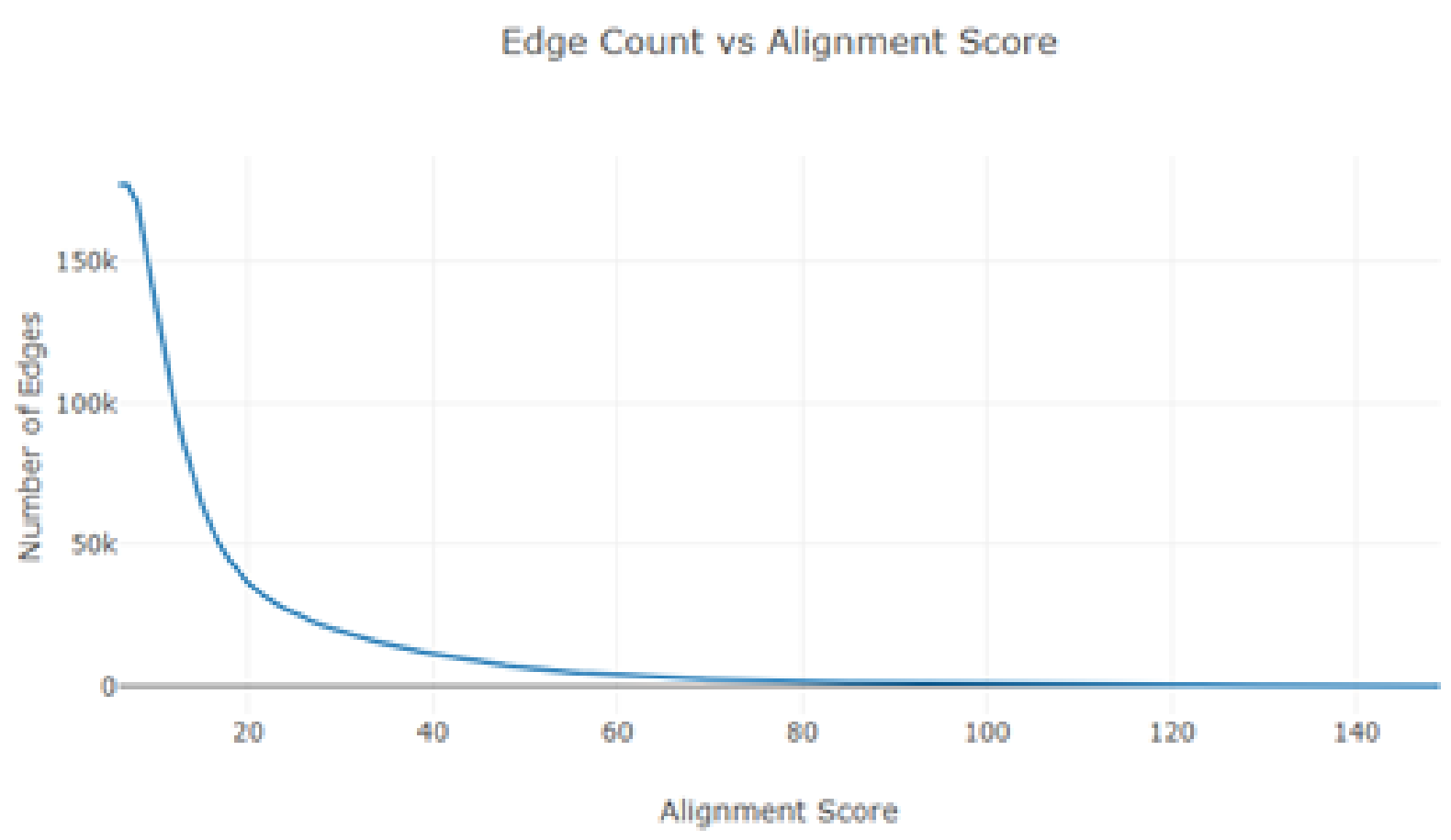
Edge Count vs Alignment Score
Number of Edges
150k
100k
50k
0
20
40
60
80
100
120
140
Alignment Score

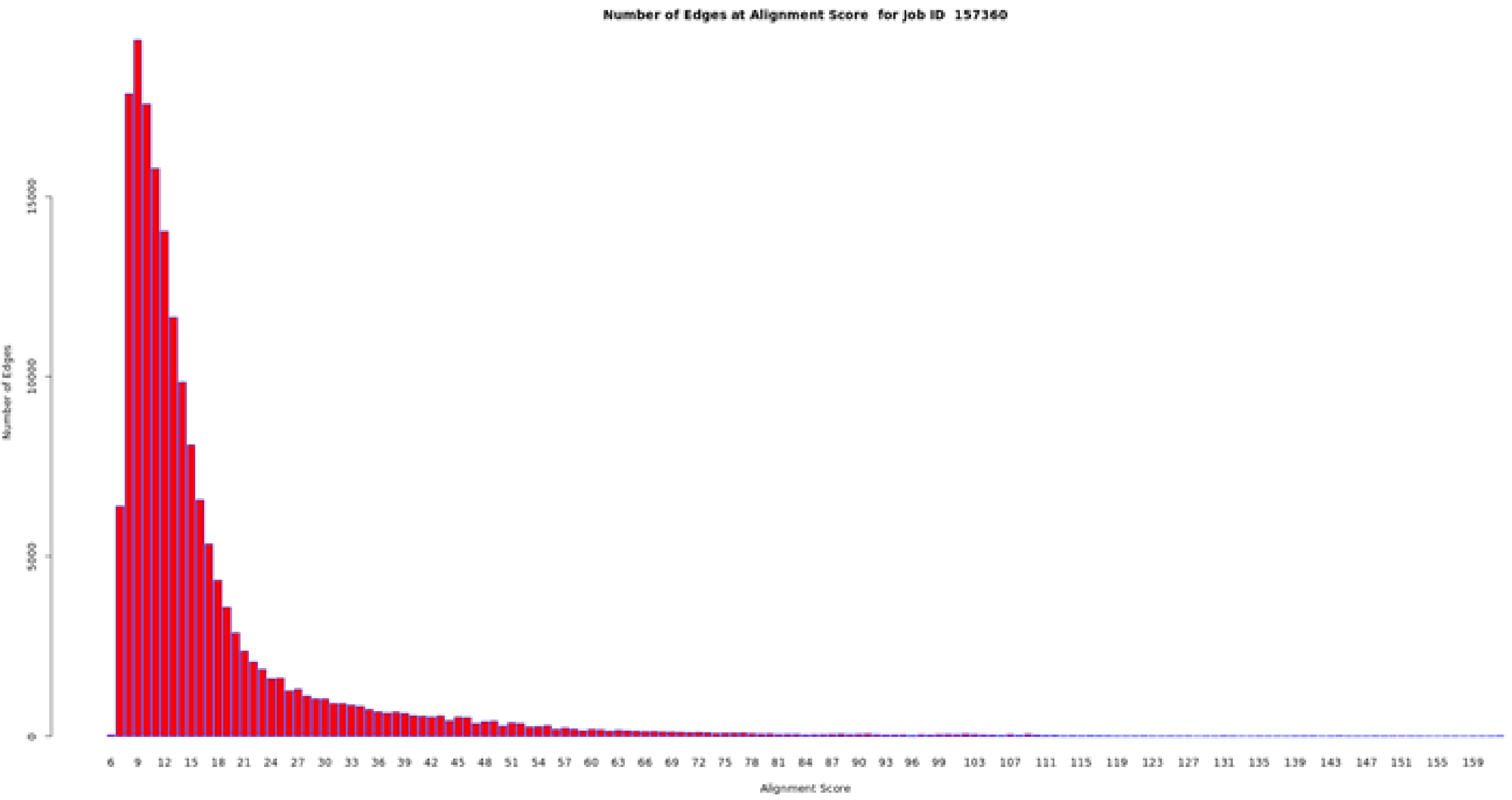

Number of Edges at Alignment Score for Job ID 157360
Number of Edges
0
5000
10000
15000
Alignment Score
6 9 12 15 18 21 24 27 30 33 36 39 42 45 48 51 54 57 60 63 66 69 72 75 78 81 84 87 90 93 96 99 103 107 111 115 119 123 127 131 135 139 143 147 151 155 159

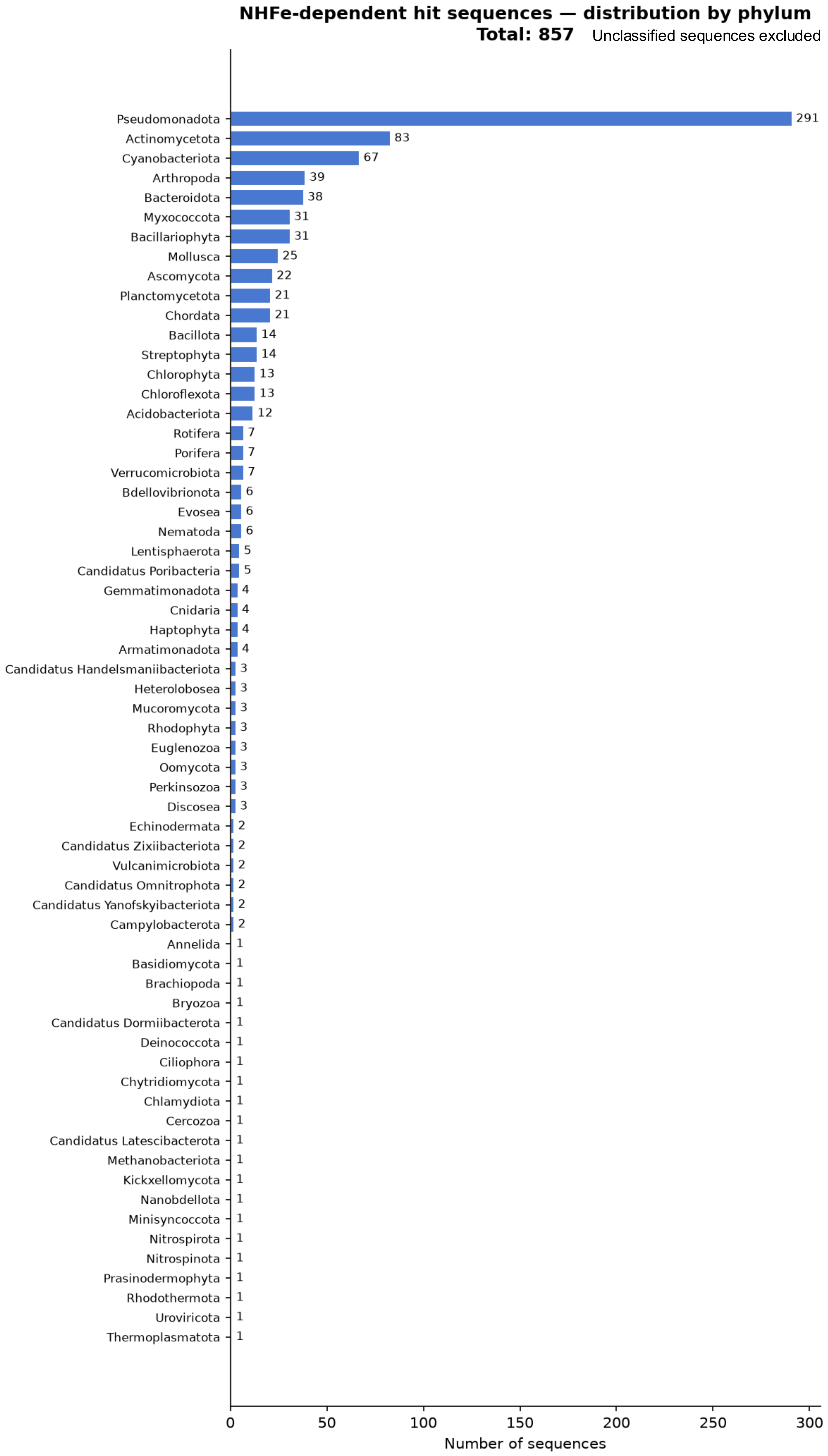

NHFe-dependent hit sequences — distribution by phylum
Total: 857
Unclassified sequences excluded
Pseudomonadota 291
Actinomycetota 83
Cyanobacteriota 67
Arthropoda 39
Bacteroidota 38
Myxococcota 31
Bacillariophyta 31
Mollusca 25
Ascomycota 22
Planctomycetota 21
Chordata 21
Bacillota 14
Streptophyta 14
Chlorophyta 13
Chloroflexota 13
Acidobacteriota 12
Rotifera 7
Porifera 7
Verrucomicrobiota 7
Bdellovibrionota 6
Evosea 6
Nematoda 6
Lentisphaerota 5
Candidatus Poribacteria 5
Gemmatimonadota 4
Cnidaria 4
Haptophyta 4
Armatimonadota 4
Candidatus Handelsmaniibacteriota 3
Heterolobosea 3
Mucoromycota 3
Rhodophyta 3
Euglenozoa 3
Oomycota 3
Perkinsozoa 3
Discosea 3
Echinodermata 2
Candidatus Zixiibacteriota 2
Vulcanimicrobiota 2
Candidatus Omnitrophota 2
Candidatus Yanofskyibacteriota 2
Campylobacterota 2
Annelida 1
Basidiomycota 1
Brachiopoda 1
Bryozoa 1
Candidatus Dormiibacterota 1
Deinococcota 1
Ciliophora 1
Chytridiomycota 1
Chlamydiota 1
Cercozoa 1
Candidatus Latescibacterota 1
Methanobacteriota 1
Kickxellomycota 1
Nanobdellota 1
Minisyncoccota 1
Nitrospirota 1
Nitrospinota 1
Prasinodermophyta 1
Rhodothermota 1
Uroviricota 1
Thermoplasmatota 1
0
50
100
150
200
250
300
Number of sequences

Copper-dependent

Job Number	168369
Time Started -- Finished	12/22 09:34 AM -- 12/22 09:42 AM
Database Version	UniProt: 2025-03 / InterPro: 106
Input Option	FASTA (Option C), no FASTA header reading
Job Name	copper-dependent
E-Value for SSN Edge Calculation	5
Uploaded FASTA File	copper-dependent_hits.fasta
Number of Sequences in Uploaded File	5,299
Exclude Fragments	No
Total Number of Sequences in Dataset	5,299
Total Number of Edges	6,040,909
Number of Unique Sequences	5,299
Convergence Ratio?	0.430

Alignment Score threshold: 50

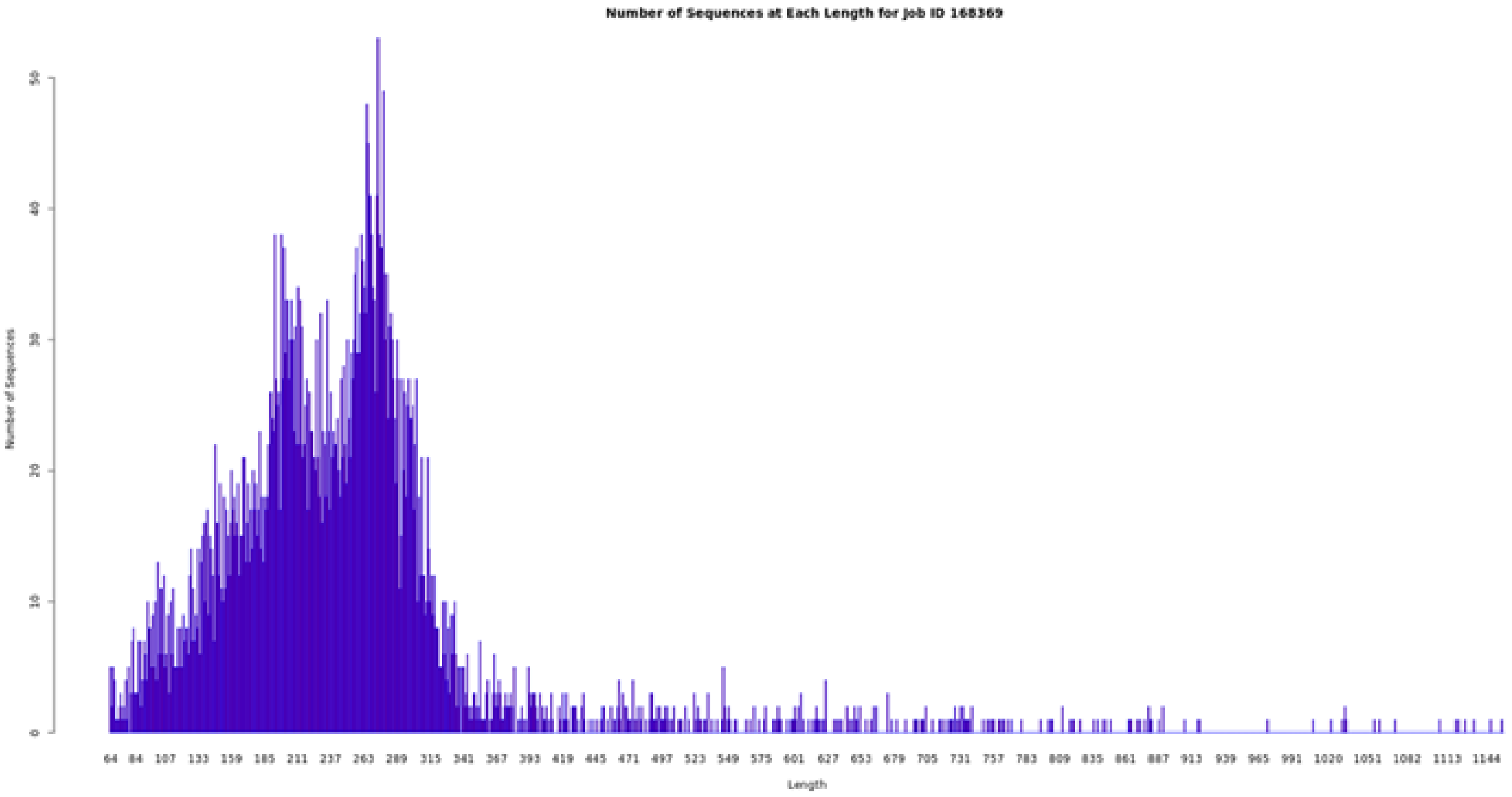

Number of Sequences at Each Length for Job ID 168369
Number of Sequences
Length

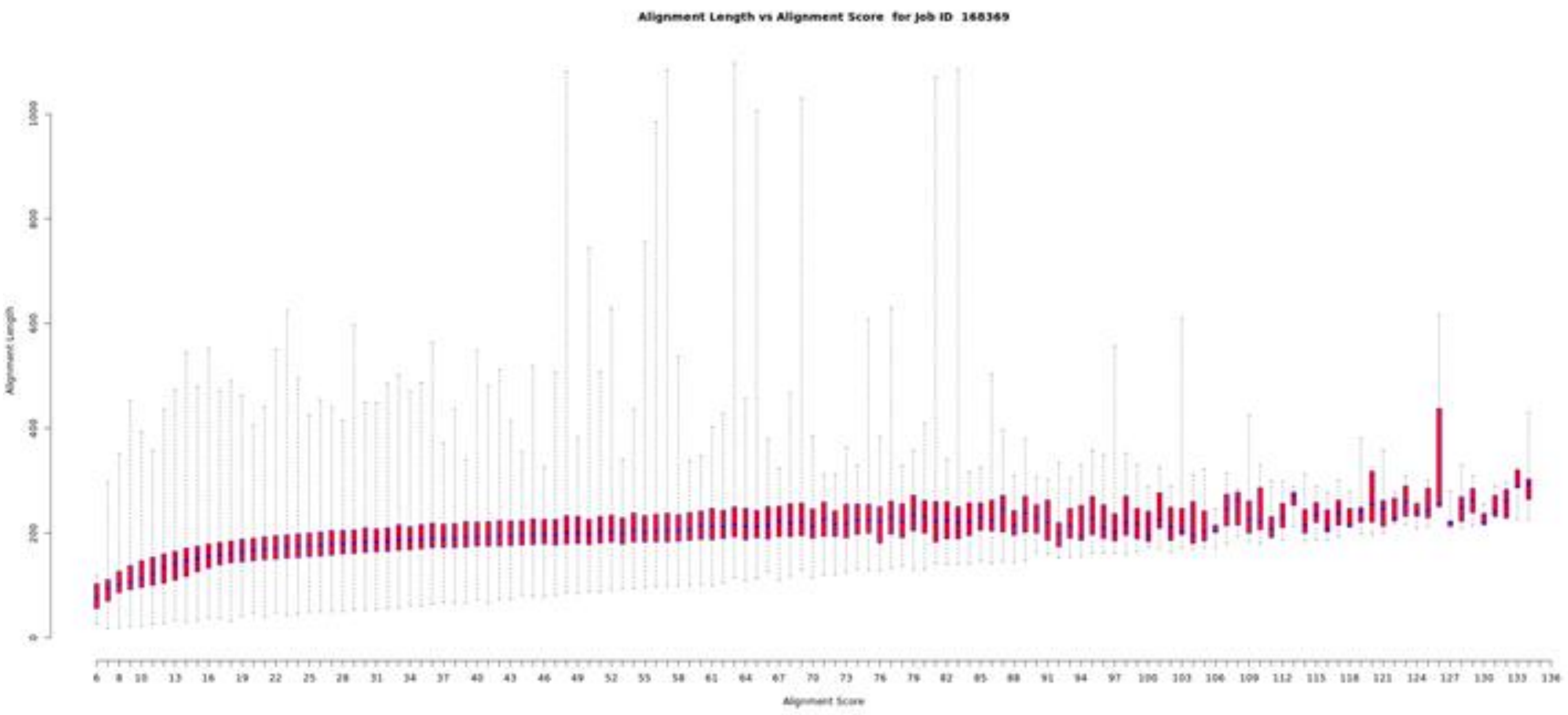
Alignment Length vs Alignment Score for Job ID 168369
Alignment Length
0
200
400
600
800
1000
Alignment Score
6 8 10 13 16 19 22 25 28 31 34 37 40 43 46 49 52 55 58 61 64 67 70 73 76 79 82 85 88 91 94 97 100 103 106 109 112 115 118 121 124 127 130 133 136

Percent Identity vs Alignment Score for Job ID 168369

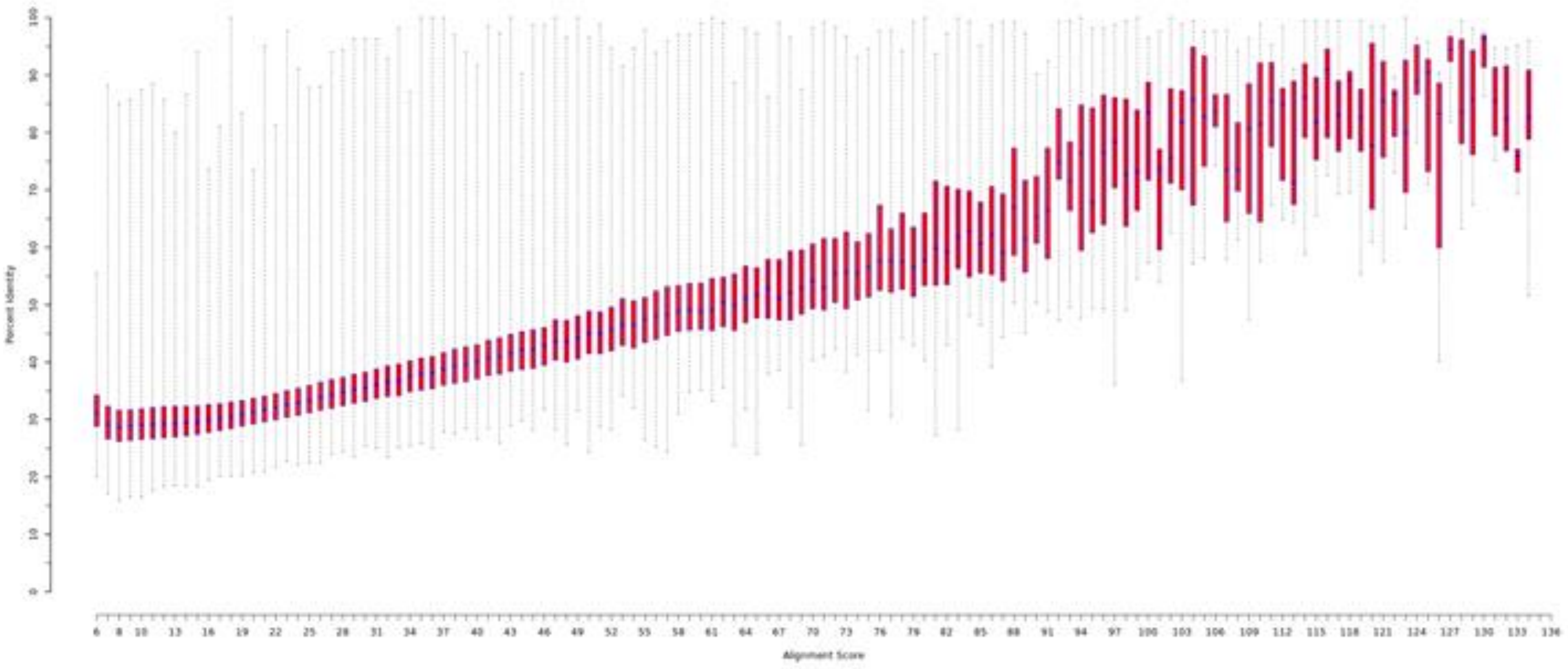

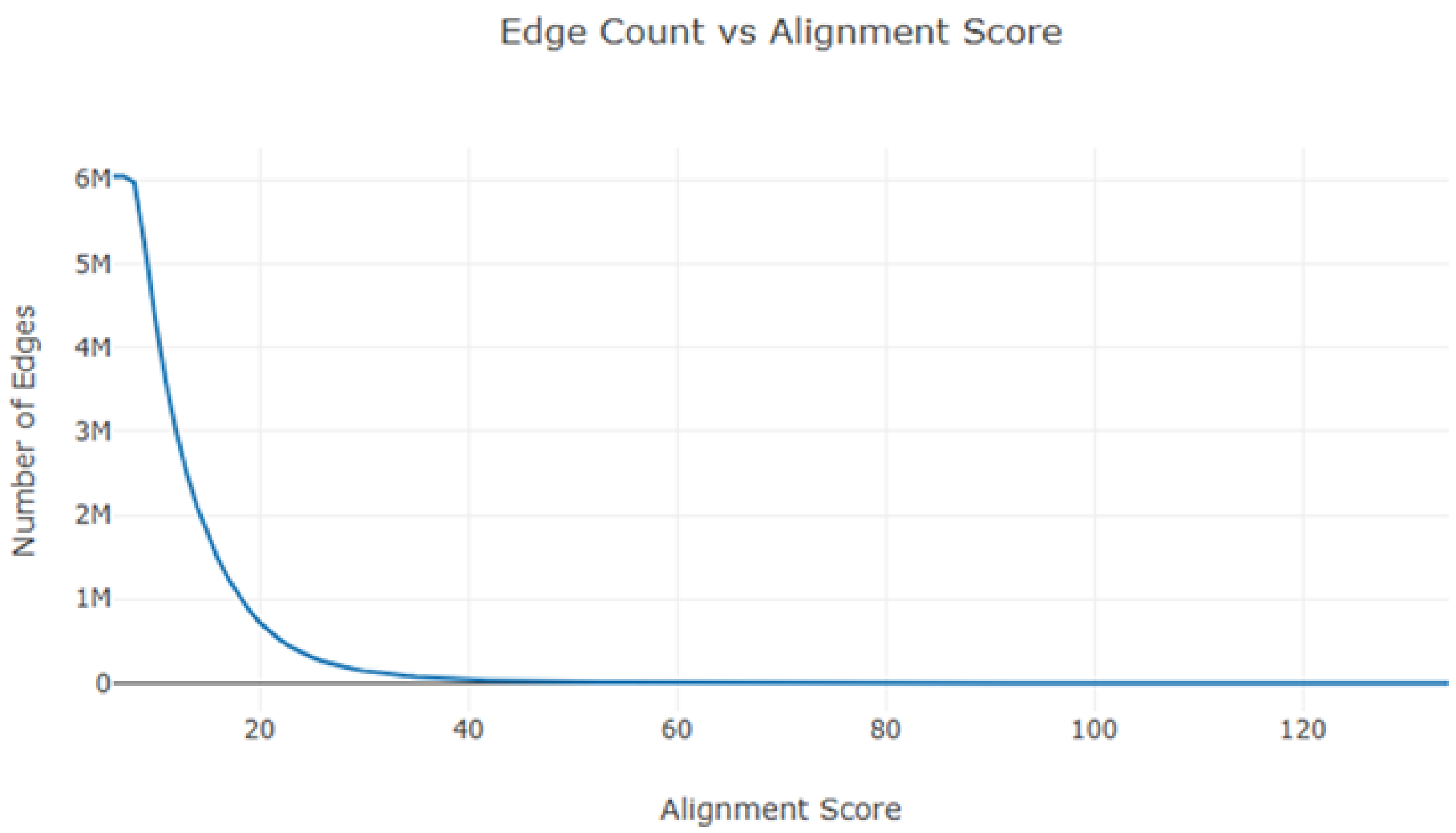

Edge Count vs Alignment Score
Number of Edges
6M
5M
4M
3M
2M
1M
0
20
40
60
80
100
120
Alignment Score

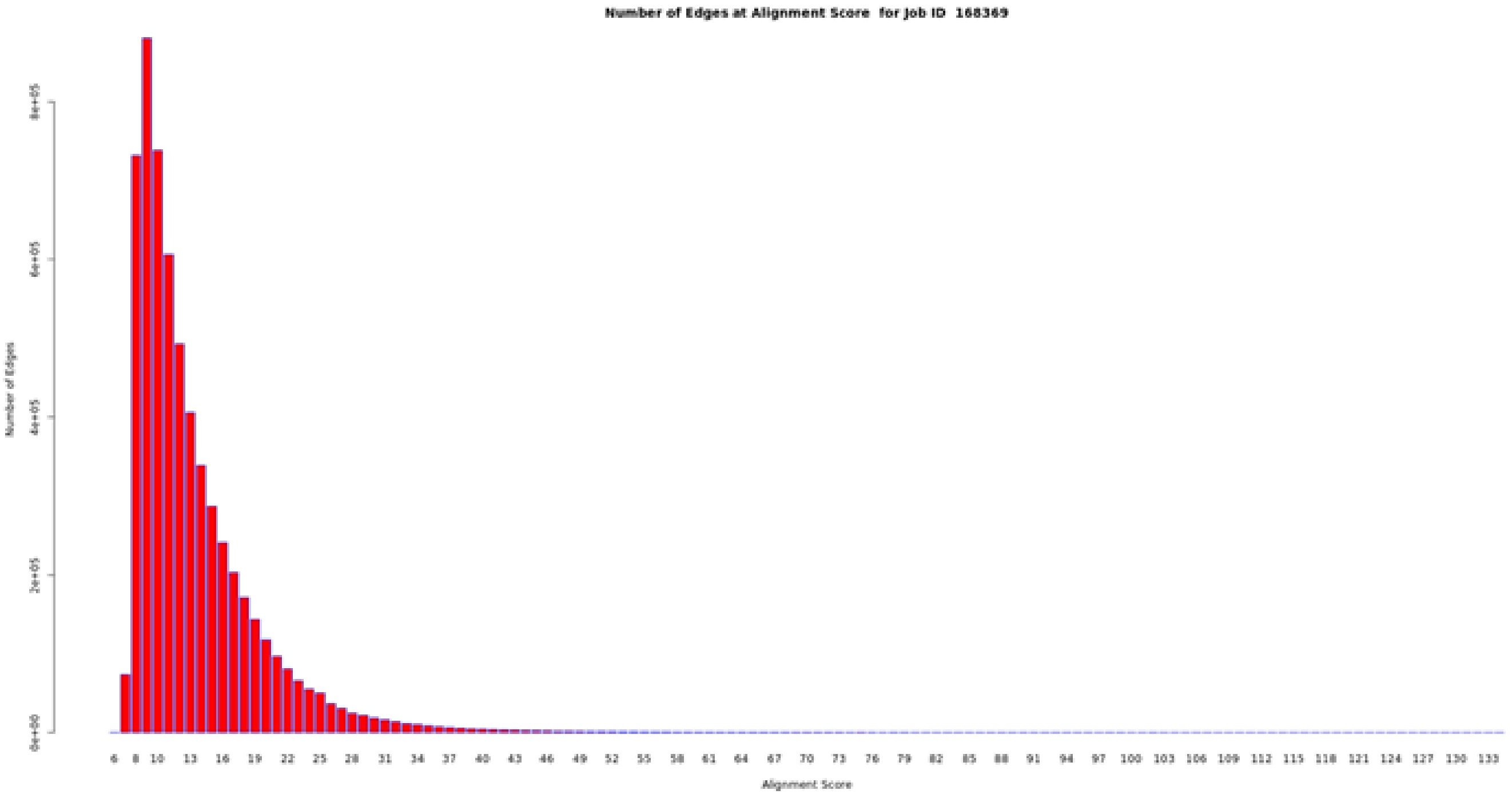

Number of Edges at Alignment Score for Job ID 168369
Number of Edges
0e+00
2e+05
4e+05
6e+05
8e+05
Alignment Score
6 8 10 13 16 19 22 25 28 31 34 37 40 43 46 49 52 55 58 61 64 67 70 73 76 79 82 85 88 91 94 97 100 103 106 109 112 115 118 121 124 127 130 133

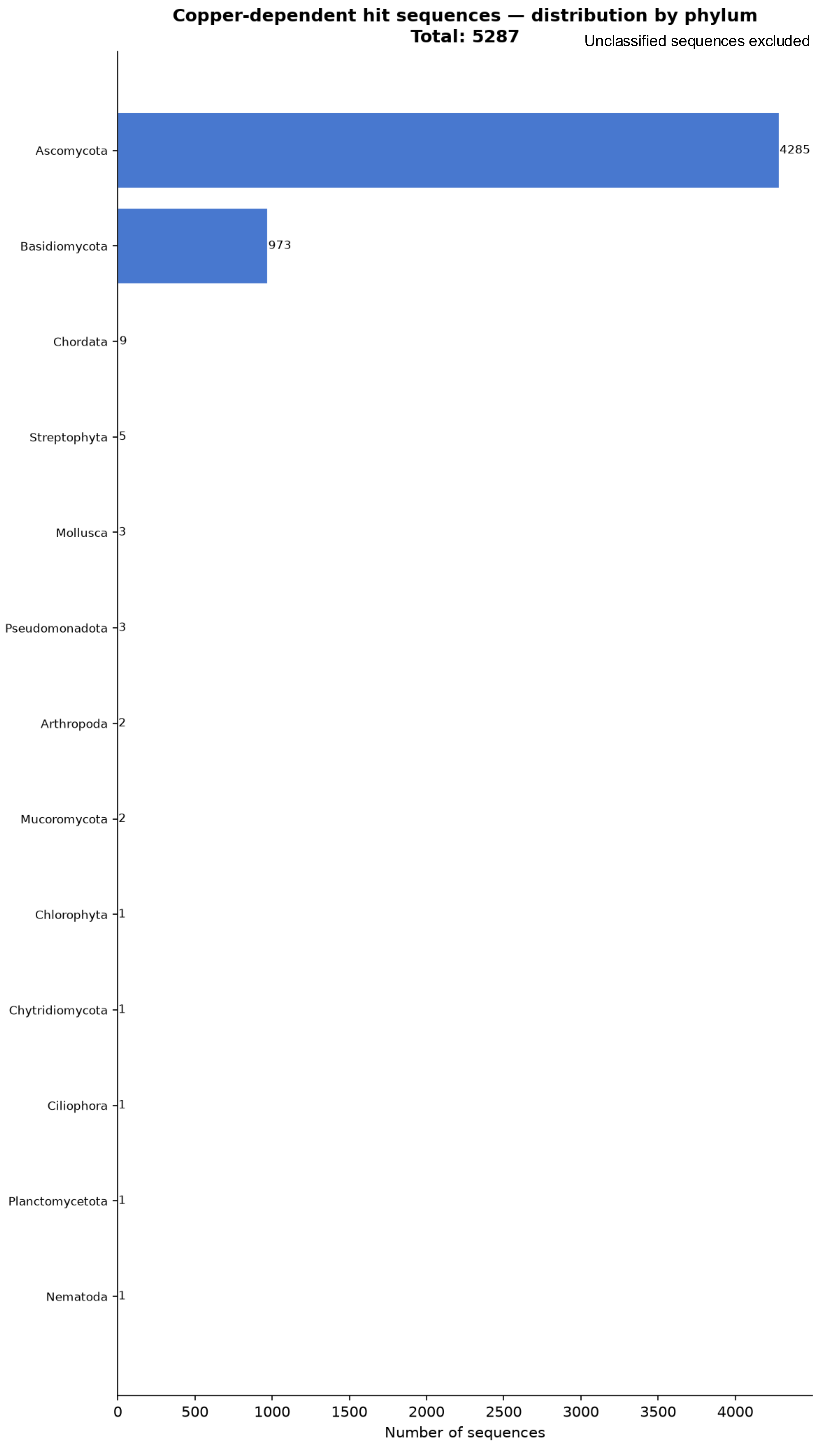
Copper-dependent hit sequences — distribution by phylum
Total: 5287
Unclassified sequences excluded
Ascomycota
4285
Basidiomycota
973
Chordata
9
Streptophyta
5
Mollusca
3
Pseudomonadota
3
Arthropoda
2
Mucoromycota
2
Chlorophyta
1
Chytridiomycota
1
Ciliophora
1
Planctomycetota
1
Nematoda
1
0
500
1000
1500
2000
2500
3000
3500
4000
Number of sequences
